# Um Marco na Internacionalização da Ressonância Magnética Cardiovascular e um Legado para o Brasil e a América Latina

**DOI:** 10.36660/abc.20260568

**Published:** 2026-09-21

**Authors:** Carlos Eduardo Rochitte, Clério Azevedo, Juliano Lara Fernandes

**Affiliations:** 1 Hospital das Clínicas Faculdade de Medicina Universidade de São Paulo São Paulo SP Brasil Instituto do Coração do Hospital das Clínicas da Faculdade de Medicina da Universidade de São Paulo, São Paulo, SP – Brasil; 2 Hcor Associação Beneficente Síria São Paulo SP Brasil Hospital do Coração (Hcor), Associação Beneficente Síria, São Paulo, SP – Brasil; 3 Alta/DASA Medicina Diagnóstica São Paulo SP Brasil Alta/DASA Medicina Diagnóstica, São Paulo, SP – Brasil; 4 Blume Medicina Diagnóstica São Paulo SP Brasil Blume Medicina Diagnóstica, São Paulo, SP – Brasil; 5 Duke University Durham EUA Duke University, Durham – EUA; 6 RDC Medicina Diagnóstica Campinas SP Brasil RDC Medicina Diagnóstica, Campinas, SP – Brasil

**Keywords:** Cardiologia, Radiologia, Congressos como Assunto, Cooperação Internacional

## Abstract

**Figura Central:**
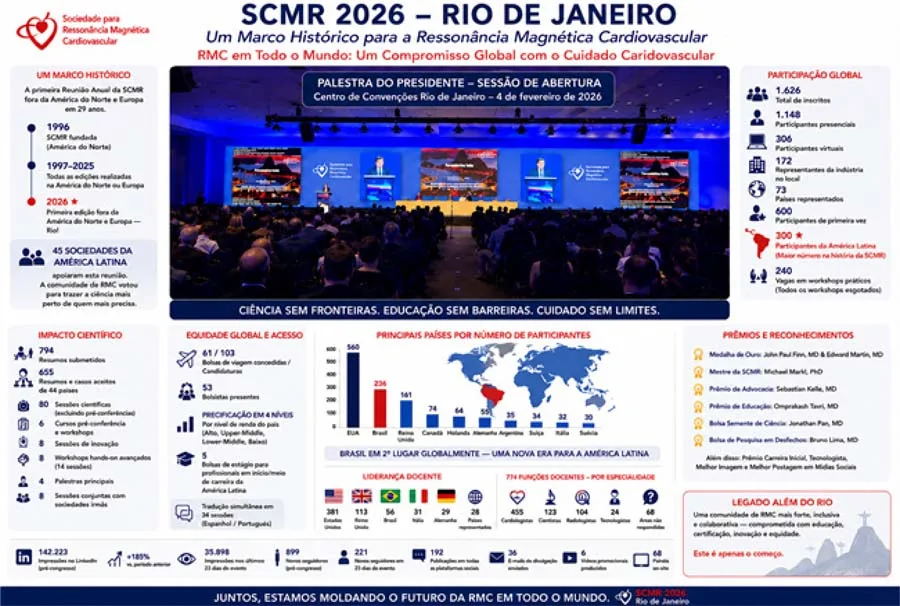
: Um Marco na Internacionalização da Ressonância Magnética Cardiovascular e um Legado para o Brasil e a América Latina

**Figura Central:** : Um Marco na Internacionalização da Ressonância Magnética Cardiovascular e um Legado para o Brasil e a América Latina

O SCMR Rio 2026 representou a passagem da internacionalização geográfica para a construção de capacidade científica regional. O encontro integrou participação global, liderança latino-americana, educação, certificação e colaboração multidisciplinar como pilares para expandir o acesso à RMC de qualidade.

Há momentos na trajetória de uma sociedade científica que transcendem a realização de um congresso. Tornam-se marcos que redefinem sua identidade, ampliam sua missão e deixam um legado duradouro para a especialidade. A realização da 29ª Reunião Científica Anual da *Society for Cardiovascular Magnetic Resonance* (SCMR), no Rio de Janeiro, de 4 a 7 de fevereiro de 2026, representa um desses momentos. Pela primeira vez em sua história, a principal sociedade internacional dedicada à ressonância magnética cardiovascular (RMC) realizou seu congresso anual fora da América do Norte e da Europa. O tema escolhido — *CMR Worldwide: A Global Commitment to Cardiovascular Care* — deixou de ser apenas um lema de congresso para se tornar uma declaração institucional sobre o futuro da especialidade.

O significado do SCMR Rio 2026 não reside apenas no fato de ter ocorrido no Brasil. Seu verdadeiro significado está no que ele demonstrou: que excelência científica, inclusão geográfica, formação de recursos humanos e colaboração multidisciplinar não são objetivos concorrentes, mas dimensões complementares de uma mesma missão. Ao levar o congresso para a América Latina, a SCMR não apenas deslocou sua reunião anual; aproximou educação, ciência e liderança de uma região com enorme carga de doença cardiovascular, grande capacidade científica e persistentes desigualdades de acesso à imagem cardiovascular avançada.

Os números finais do encontro documentam essa mudança (Material suplementar e Figura Central). O SCMR 2026 reuniu mais de 1.600 participantes, com representação de 73 países e cerca de 600 participantes de primeira vez. O Brasil foi o segundo país em número de participantes, atrás apenas dos Estados Unidos, e a América Latina atingiu sua maior representação histórica em uma reunião anual da SCMR. O programa científico recebeu 794 submissões, das quais 655 abstracts e casos foram aceitos, provenientes de 44 países. Foram realizadas 80 sessões científicas, seis cursos ou workshops pré-congresso, oito sessões de inovação, oito workshops práticos avançados realizados em 14 sessões, todos com lotação esgotada, além de 34 sessões com tradução simultânea para português ou espanhol. Esses números não são meros indicadores administrativos; eles revelam o efeito concreto da redução de barreiras geográficas, linguísticas e econômicas (Material suplementar).

A presença de líderes mundiais no SCMR 2026, no Rio de Janeiro, foi um dos grandes destaques do congresso. O professor João Lima, da Johns Hopkins University, abriu o evento com uma palestra inesquecível sobre como a RMC e a SCMR caminharam juntas ao longo dos anos em uma trajetória extraordinária de ciência, descoberta e impacto global: “*CMR and SCMR Journey: Science, Discovery, and Global*”.

Ainda na sessão de abertura, o Dr. Steve Levine, um dos maiores especialistas mundiais em tecnologia de gêmeos digitais, apresentou uma palestra emocionante, entrelaçando uma história pessoal envolvendo sua filha com a evolução dessa tecnologia, em “*The Living Heart: How Digital Twins Are Transforming Cardiovascular Medicine*”.

Na sessão de encerramento, o professor Raymond Kim, da Duke University, brindou os participantes com uma reflexão brilhante sobre qual será a próxima grande quebra de paradigma na RMC. Naturalmente, a inteligência artificial ocupou papel central, mas, como sempre em suas apresentações marcantes, houve também uma visão mais ampla e inspiradora sobre o futuro da área: “*What is the Next Paradigm Shift for CMR*”.

Por fim, o professor Christopher Kramer, então Presidente do *American College of Cardiology*, encerrou o SCMR 2026 com chave de ouro, abordando o significado da RMC e da SCMR para a saúde cardiovascular global em sua palestra “*CMR and the Future of Global Cardiovascular Health*”.

Um dado particularmente expressivo foi a presença latino-americana. O relatório final do congresso registrou aproximadamente 300 participantes da América Latina, o maior número já alcançado pela região na história da SCMR^1^ (Material suplementar). Além disso, 45 sociedades latino-americanas apoiaram formalmente a realização do encontro, e o pré-congresso dedicado à América Latina reuniu participantes de múltiplos países da região. Esses elementos indicam que o Rio 2026 não foi uma visita pontual da SCMR à América Latina, mas o resultado de uma construção coletiva e de uma base regional previamente existente.

O Brasil ocupa posição singular nessa história. Nas últimas duas décadas, a RMC brasileira evoluiu de forma consistente: inicialmente incorporando conhecimento internacional, depois formando especialistas, produzindo diretrizes lideradas pela SBC,^2^ participando de estudos multicêntricos e, finalmente, contribuindo para a formação de profissionais de outros países. O desenvolvimento da RMC no país ocorreu em um ambiente peculiarmente multidisciplinar, no qual cardiologistas e radiologistas participaram conjuntamente da consolidação da modalidade. A Sociedade Brasileira de Cardiologia, o Colégio Brasileiro de Radiologia e Diagnóstico por Imagem, a Sociedade Paulista de Radiologia e sociedades regionais contribuíram, em diferentes momentos, para diretrizes, cursos, reuniões científicas e discussões de qualidade em imagem cardiovascular.

Dados recentes apresentados por lideranças brasileiras da área reforçam esse panorama.^3^ O Brasil tem uma população superior a 200 milhões de habitantes, 27 unidades federativas e aproximadamente 40 centros realizando RMC. Em 2024, estimou-se a realização de cerca de 15 mil exames de RMC no sistema público e aproximadamente 44 mil no setor privado, revelando tanto a expansão da modalidade quanto a desigualdade de acesso entre sistemas de saúde. A distribuição de profissionais também permanece heterogênea, com maior concentração nas regiões Sul e Sudeste, associada à maior disponibilidade de serviços privados e infraestrutura tecnológica. O desafio brasileiro, portanto, não é apenas aumentar a produção de exames, mas ampliar a qualidade, a distribuição e a equidade do acesso.

Nesse sentido, a realização do SCMR no Rio teve valor simbólico e prático. Para muitos jovens cardiologistas, radiologistas, físicos, tecnólogos e pesquisadores latino-americanos, participar de uma reunião internacional desse porte tornou-se possível pela primeira vez. A presença de 600 participantes estreantes é talvez uma das evidências mais importantes do impacto do local. A ciência não se expande apenas pela publicação de artigos ou pela criação de diretrizes; ela também se expande quando novos profissionais entram em contato direto com uma comunidade científica, encontram mentores, apresentam seus primeiros trabalhos e passam a se enxergar como parte de uma rede internacional.

A estrutura do SCMR 2026 foi desenhada com essa intenção. As categorias diferenciadas de inscrição por renda dos países, a tradução simultânea, os grants de viagem, as sessões conjuntas com sociedades-irmãs, o espaço para sociedades latino-americanas e os workshops práticos compuseram uma estratégia de inclusão ativa. Ao todo, 61 bolsas de viagem foram concedidas, e 53 bolsistas efetivamente participaram do encontro. A presença de 774 funções de palestrantes, moderadores e painelistas, distribuídas em 28 países, incluindo 56 funções de brasileiros, mostra que a região não esteve presente apenas como audiência, mas também como liderança científica.

Outro aspecto essencial do legado do Rio 2026 é a educação. Congressos inspiram; programas de treinamento e certificação sustentam excelência. Desde seus primeiros anos, a SCMR reconheceu que a expansão segura da RMC exige critérios claros de competência. Em 2000, a Sociedade publicou suas primeiras diretrizes de credenciamento em RMC, definindo níveis de treinamento e experiência. Documentos subsequentes, incluindo as diretrizes de treinamento de 2007 e 2018 e o currículo baseado em competências publicado em 2024, consolidaram uma estrutura internacionalmente reconhecida para formar profissionais capazes de executar, interpretar e reportar exames de RMC com qualidade.

Essa evolução é particularmente relevante para regiões em desenvolvimento. A certificação não deve ser entendida como barreira corporativa, mas como instrumento de democratização da qualidade. Em países com grande heterogeneidade de infraestrutura e treinamento, programas de certificação oferecem referência objetiva para médicos, serviços, gestores e pacientes. Ao estabelecer competências mínimas para prática independente, incluindo conhecimento clínico, protocolos de aquisição, interpretação cardiovascular, segurança, farmacologia do estresse, reconhecimento de achados extracardíacos relevantes e participação em programas de melhoria de qualidade, a certificação ajuda a transformar entusiasmo em prática sustentável.^4-7^

O documento de competência da SCMR de 2024 é especialmente importante porque foi concebido para ser globalmente relevante e aplicável a profissionais oriundos tanto da cardiologia quanto da radiologia.^8^ Essa visão é central para a RMC. A modalidade nasceu e amadureceu na interseção entre disciplinas. Cardiologistas trazem conhecimento profundo da fisiologia, da clínica cardiovascular, da estratificação de risco e da tomada de decisão terapêutica. Radiologistas trazem expertise em física da imagem, segurança, interpretação anatômica ampla e reconhecimento de achados extracardíacos. Físicos, engenheiros, tecnólogos, cientistas de dados e pesquisadores translacionais expandem os limites técnicos e científicos da área. A RMC é mais forte quando todas essas competências trabalham juntas.^9-11^

Esse princípio foi recentemente reafirmado em carta institucional da SCMR dirigida à comunidade internacional de imagem cardiovascular. A Sociedade declarou apoiar tanto cardiologistas quanto radiologistas que tenham alcançado competência adequada para reportar e assinar exames clínicos de RMC de forma independente. O documento enfatiza que o critério essencial deve ser treinamento, competência, experiência e julgamento clínico, e não a fronteira formal entre especialidades. A carta também destaca que o modelo ideal de serviço de RMC inclui colaboração entre cardiologistas e radiologistas, com disponibilidade para segundas opiniões em casos cardiovasculares ou extracardíacos mais complexos. Essa visão é particularmente importante para o Brasil e a América Latina, onde a expansão da RMC exige somar competências, não criar barreiras.

A colaboração multidisciplinar também deve orientar o relacionamento entre sociedades científicas. O material apresentado por lideranças brasileiras durante o SCMR 2026 mostrou que, no Brasil, diretrizes, cursos e eventos em imagem cardiovascular têm sido frequentemente construídos por meio de interação entre a Sociedade Brasileira de Cardiologia, o Colégio Brasileiro de Radiologia e a Sociedade Paulista de Radiologia. Essa experiência local pode servir como exemplo para outros países. Em vez de reproduzir disputas por território profissional, a América Latina tem a oportunidade de desenvolver um modelo colaborativo centrado em qualidade, treinamento e acesso do paciente.

O impacto para a cardiologia brasileira deve ser visto nesse contexto mais amplo. A RMC é hoje ferramenta essencial no diagnóstico e manejo de cardiomiopatias, miocardites, cardiopatias congênitas, doença arterial coronariana, valvopatias, massas cardíacas, doenças sistêmicas com acometimento cardíaco e avaliação de viabilidade miocárdica. Na América Latina, sua relevância é ampliada por doenças com forte expressão regional, como a cardiomiopatia chagásica e a cardiopatia reumática, além da carga crescente de insuficiência cardíaca, doença coronariana e cardio-oncologia. Expandir o acesso à RMC de qualidade não é um luxo tecnológico; é uma estratégia para melhorar diagnóstico, prognóstico e decisões terapêuticas em populações de alto risco.

O SCMR Rio 2026 também ocorreu em um momento de transformação institucional da própria Sociedade. No mesmo ciclo, a SCMR avançou para um modelo de gestão autônoma, reduzindo sua dependência de uma empresa externa de gerenciamento e construindo sua própria equipe operacional. Essa mudança, menos visível ao público do que a realização de um congresso histórico, é igualmente relevante. Uma sociedade autogerida tem maior capacidade de alinhar estratégia, educação, certificação, defesa de interesses, comunicação e sustentabilidade financeira. Em conjunto, a primeira reunião fora da América do Norte e Europa e a transição para autogestão representam dois marcos complementares: a SCMR mudou onde se encontra e como se organiza.

O legado do Rio deve, portanto, ser medido nos próximos anos. Ele será percebido em novos centros de RMC, no aumento de profissionais certificados, em projetos multicêntricos latino-americanos, em maior participação de jovens pesquisadores em reuniões internacionais, em diretrizes regionais, em programas de treinamento mais estruturados e em maior integração entre cardiologistas e radiologistas. O sucesso de um congresso dura alguns dias; o sucesso de um movimento científico dura décadas.

Os Arquivos Brasileiros de Cardiologia têm papel fundamental nesse processo. Como principal periódico cardiovascular do país e uma das vozes científicas mais relevantes da América Latina, os ABC Cardiol podem contribuir para disseminar evidências de RMC geradas na região, estimular debates sobre acesso e qualidade, promover diretrizes baseadas em realidade local e aproximar a comunidade clínica das possibilidades oferecidas pela imagem cardiovascular avançada. A publicação de um editorial sobre o SCMR Rio 2026 neste periódico não deve ser entendida como celebração institucional, mas como registro de um novo capítulo da cardiologia brasileira.

O Rio de Janeiro demonstrou que excelência científica não é determinada por geografia. Quando educação, certificação, colaboração multidisciplinar e inclusão global são combinadas de forma deliberada, a ciência se expande sem perder rigor. A RMC não pertence a um país, a uma especialidade ou a um grupo profissional. Pertence aos pacientes que dela se beneficiam. O SCMR Rio 2026 foi muito além de um congresso: foi a demonstração de que a comunidade internacional de RMC está pronta para uma fase verdadeiramente global, na qual o Brasil e a América Latina deixam de ocupar a periferia e passam a ocupar papel central na construção do futuro da imagem cardiovascular.

O verdadeiro legado do SCMR Rio 2026 não será medido apenas pelo número de participantes, sessões ou trabalhos apresentados. Será medido pelos profissionais que foram inspirados, pelos serviços que serão aprimorados, pelos centros que serão criados, pelas pesquisas que nascerão de novas colaborações e, sobretudo, pelos pacientes que terão acesso a uma RMC de melhor qualidade graças ao conhecimento, à confiança e à rede construída durante aqueles quatro dias no Rio de Janeiro.

## Material suplementar

Para informação adicional, por favor, clique aqui.

Material suplementar
