# Supplementary material for: Um Marco na Internacionalização da Ressonância Magnética Cardiovascular e um Legado para o Brasil e a América Latina
Source: Arq Bras Cardiol. 2026 Sep 21;123(9):e20260568. [Article in Portuguese] doi: 10.36660/abc.20260568 (PMC13649070; doi:10.36660/abc.20260568)
Supplement: Supplementary file 2 [file 0066-782x-abc-123-09-e20260568-suppl01.pdf]

SCMR 2026 REPORT

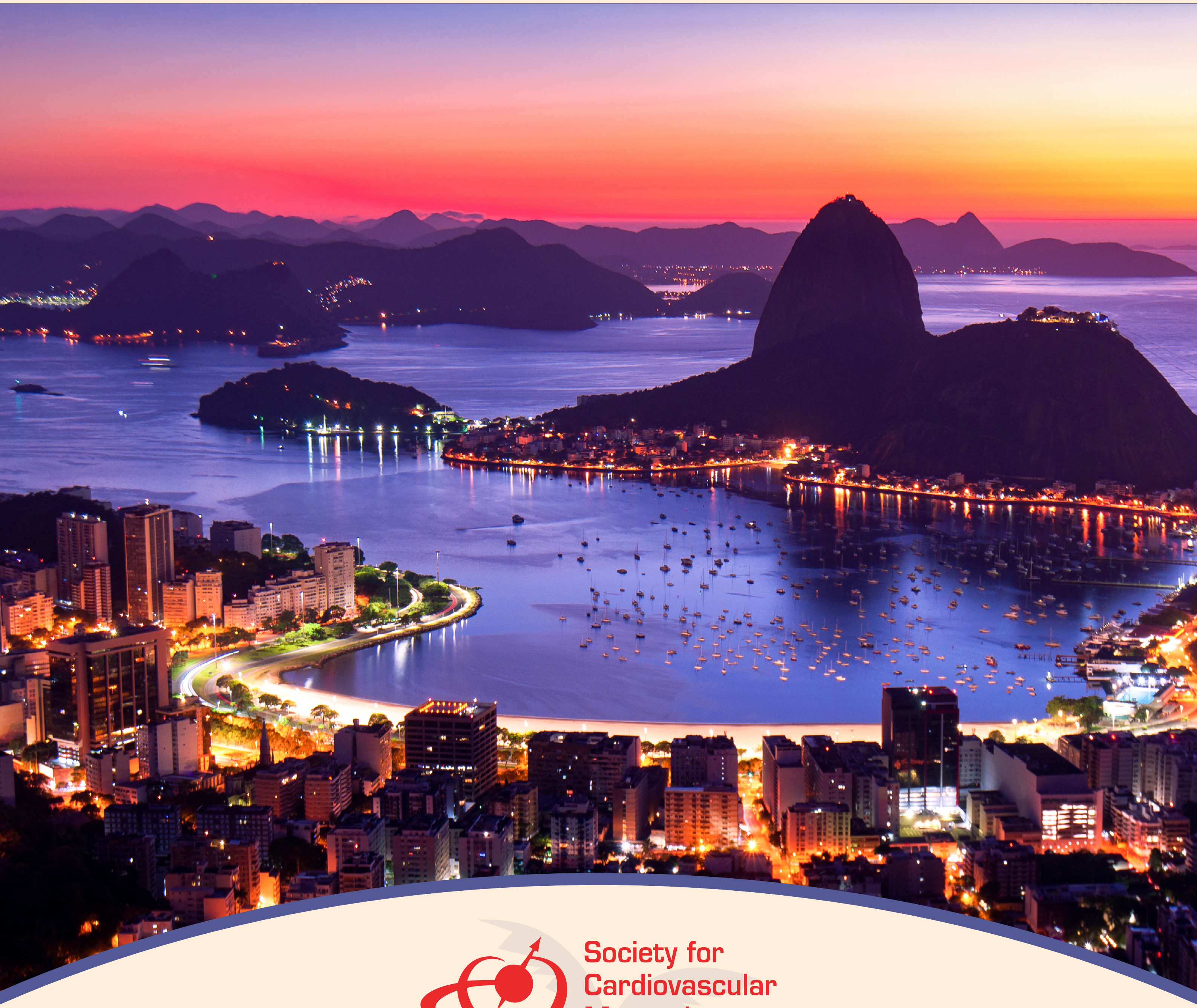

Society for Cardiovascular Magnetic Resonance

SCMR 29TH ANNUAL SCIENTIFIC SESSIONS

4-7 February, 2026

Rio de Janeiro, Brazil

CMR Worldwide: A Global Commitment to Cardiovascular Care

TABLE OF CONTENTS

SCMR 2026 Leadership & Program Committee .... Page 3
Registration Overview .... Page 4
Attendees by Country .... Page 6
Abstracts & Cases .... Page 7
Travel Grant Awards .... Page 8
Invited Faculty Members .... Page 9
SCMR 2026 Highlights .... Page 10
Joint Sessions .... Page 10
Symposium .... Page 11
Sponsored Hands-on Sessions .... Page 13
Coffee Break Sponsorship .... Page 14
Industry Partners .... Page 14
Keynote Speakers .... Page 15
2025 Gold Medal Talks .... Page 15
SCMR Awards .... Page 16
Early Career Awards .... Page 17
Technologist Awards .... Page 17
Additional SCMR 2026 Awards .... Page 18
2026 SCMR Grants .... Page 19
SCMR 2026 - Community Building & Networking .... Page 20
Marketing & Communication - Online .... Page 22
Marketing & Communication - On-site .... Page 25
Acknowledgements .... Page 30
SCMR 2027 .... Page 31

SCMR 2026 LEADERSHIP & PROGRAM COMMITTEE

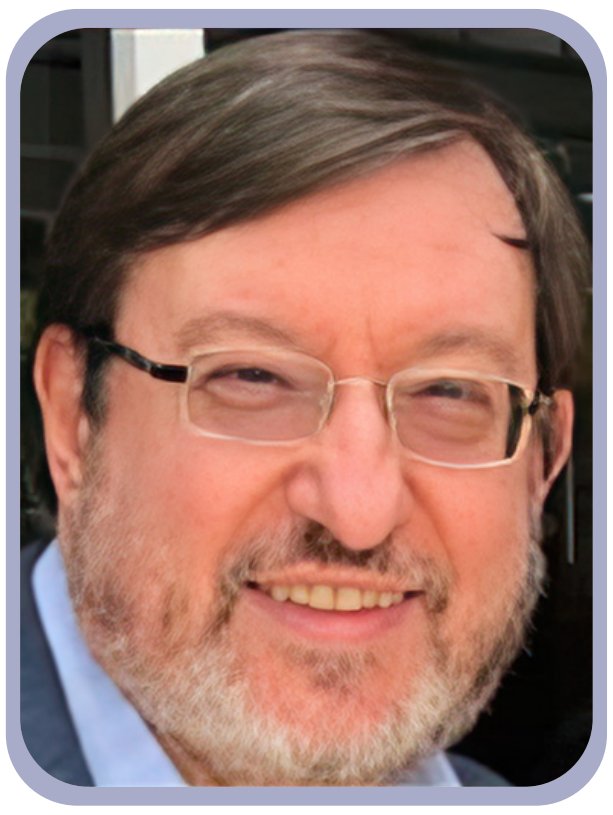

SCMR President
Carlos Rochitte, MD, PhD, FSCMR

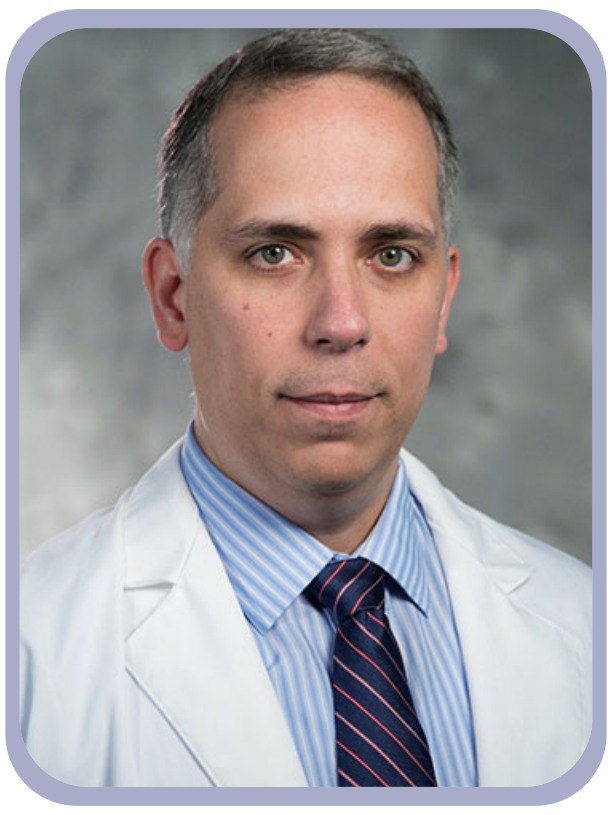

Program Chair
Clerio De Azevedo Filho, MD, FSCMR

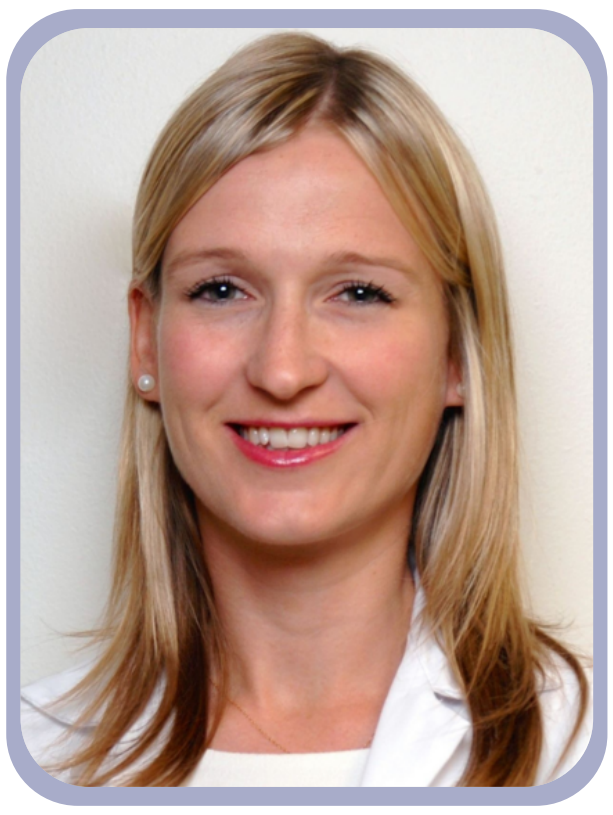

Clinical Co-Chair
Kate Hanneman, MD, MPH

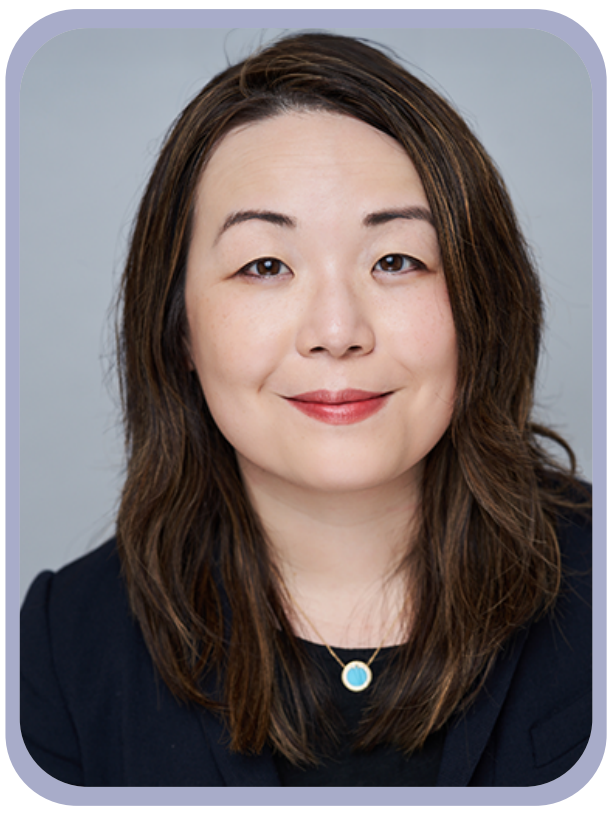

Abstract Chair
Kanae Mukai, MD

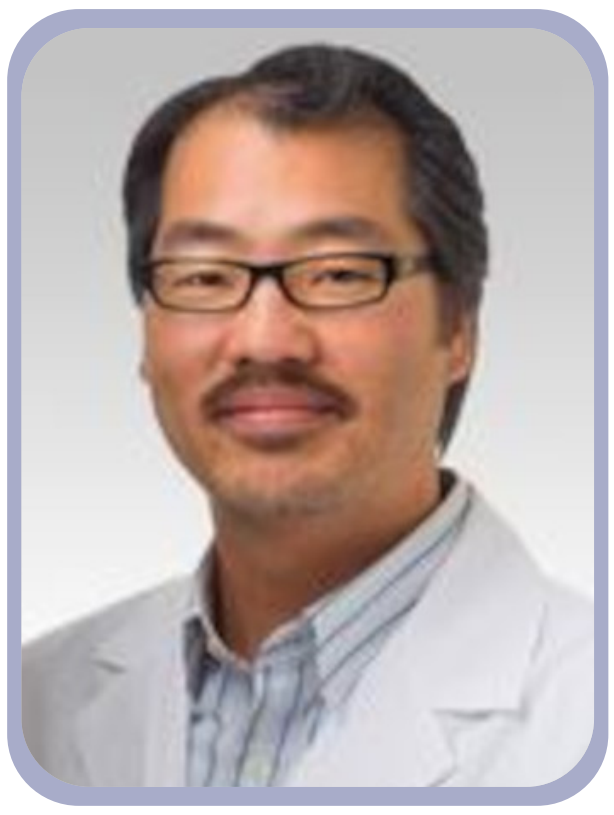

Science Chair
Daniel Kim, PhD, FSCMR

Technologist Section Representatives

Rebecca Mills
Filippo Bosio

Interventionist Section Representatives

Adrienne Campbell

Peds/CHD Section Representatives

Burkhardt Barbara
Malenka Bissel
Morin Cara
Olivieri Laura
Rahul Rathod
Joshua Robinson
Balasubramanian Sowmya

Women in CMR Section Representative

Patricia Bandettini

Karima Addetia
Mehmet Akcakaya
Bradley Allen
Jorge Andion Torreão
Mona Bhatia
Gabriella Captur
Andrea Cardona
Anthony Christodolou
Daniel Ennis
Juliano Fernandes
Hildo Lamb
Gabriela Liberato
Bruno Lima
Harold Litt
Robert Manka
Hector Medina
Martha Morelos
Luigi Natale
Ming-Yen Ng
Robin Nijveldt
Cesar Nomura
Sabrina Nordin
Vineeta Ojha
Ellen Ostenfeld
Purvi Parwani
Amit Patel
Dana Peters
Claudia Prieto
Prabhakar Rajiah
Otavio Rizzi Coelho-Filho
Felicia Seemann
Nicole Seiberlich
Henrique Trad
Marly Uellendahl
Akos Varga-Szemes
Sebastian Weingärtner

REGISTRATION OVERVIEW

High Income Country	Early Bird	Standard	TOTAL
Member	373	76	**449**
Non-Member	41	30	**71**
Member Techs/Allied Health (same rate)	-	32	**32**
Non-Member Techs/Allied Health (same rate)	-	18	**18**
Member Trainee	167	22	**189**
Non-Member Trainee	49	5	**54**
**TOTAL**	**630**	**183**	**813**

Upper-Middle Income Country	Early Bird	Standard
Member (same rate)	-	214
Non-Member (same rate)	-	75
**TOTAL**	-	**289**

Lower-Middle Income Country	Early Bird	Standard
Member (same rate)	-	19
Non-Member (same rate)	-	12
**TOTAL**	-	**31**

Low-Income Country	Early Bird	Standard
Member (same rate)	-	7
Non-Member (same rate)	-	8
**TOTAL**	-	**15**

In-Person Registrants	High	Upper-mid	Low-Mid	Low	TOTAL
Members	670	214	19	7	**910**
Non-Members	143	75	12	8	**238**
**TOTAL**	**813**	**289**	**31**	**15**	**1,148**

Virtual Registrants	High	Upper-mid	Low-Mid	Low	TOTAL
Virtual Recordings (No CME)	-	-	-	-	**4**
Members	133	33	42	9	**217**
Members Technologist/ Allied Health	5	-	-	-	**5**
Members Trainee	33	-	-	-	**33**
Non-Members	10	9	4	16	**39**
Non-Members Trainee	8	-	-	-	**8**
					**306**

1st Time Attendee	TOTAL
Yes	600
No	1,026
**TOTAL**	**1,626**

Full Meeting	TOTAL
Total Paid In-Person Registrants	1,148
Total Paid Virtual Registrants	306
Total Industry Registrants	172
**TOTAL 2026 Attendees (on-site)**	**1,324**

REGISTRATION OVERVIEW

Speciality	TOTAL
Cardiologist	725
Interventionalist	12
Non-Medical	28
Non-Physician Scientist	126
Nuclear Medicine	2
Pediatric/Congenital	108
Physician (other specialty)	25
Radiologist	272
Technologist/Allied Health	68
Trainee	100
Exhibitor	160
**TOTAL**	**1,626**

Preconferences	TOTAL
Congenital Preconference	350
iCMR Preconference	95
Latin America Meeting Preconference	73
Physician's Preconference	300
SCMR-ISMRM Co-Provided Workshop	269
Turning Innovations into Action Preconference	94
I do not plan to attend	445
**TOTAL**	**1,626**

Hands-On Advanced Sponsored Workshops	TOTAL
Introduction to 4D Flow (Pie)	30
Quantifying Flow: A Hands-On Workshop on 4D Flow Data Analysis (Circle)	30
Systolic and Diastolic Heart Failure (NeoSoft)	30
In 2D and 4D Flow We Trust! (NeoSoft)	30
Show Me the Case: Mastering Real-World CMR with Advanced Post-Processing (Medis)	30
Advanced 4D Flow: A Selection of Cases (Pie)	30
The Future of Stress Testing: Precision Quantitative Perfusion by CMR (Circle)	30
Show Me the Case: Mastering Real-World CMR with Advanced Post-Processing (Medis)	30
**TOTAL**	**240**

ATTENDEES BY COUNTRY

IN-PERSON AND VIRTUAL

Albania	2	Greece	7	Romania	4
Algeria	2	Hong Kong	2	Russian Federation	2
Angola	1	India	28	Saudi Arabia	3
Argentina	35	Indonesia	3	Serbia	1
Australia	29	Ireland	2	Singapore	5
Azerbaijan	2	Israel	2	Slovakia	1
Belgium	5	Italy	32	Slovenia	1
Bolivia	1	Jamaica	2	South Africa	8
Brazil	236	Japan	16	Spain	1
Bulgaria	4	Jordan	1	Sri Lanka	1
Cambodia	1	Republic of Korea	1	Sudan	2
Canada	74	Malaysia	8	Sweden	30
Chile	19	Mexico	13	Switzerland	34
China	11	Morocco	2	Syrian Arab Republic	4
Colombia	13	Mozambique	1	Taiwan	2
Costa Rica	4	Netherlands	64	Thailand	12
Cuba	1	New Zealand	6	Tunisia	1
Denmark	1	Oman	1	Turkey	3
Dominican Republic	1	Pakistan	6	United Arab Emirates	3
Ecuador	4	Panama	3	United Kingdom	161
Egypt	18	Paraguay	1	United States	560
El Salvador	1	Peru	11	Uruguay	3
Finland	1	Philippines	11	Vietnam	1
France	25	Portugal	18		
Germany	55	Qatar	1		

TOTAL COUNTRIES

73

TOP 10 COUNTRIES BY ATTENDANCE

1. United States: 560
2. Brazil: 236
3. United Kingdom: 161
4. Canada: 74
5. Netherlands: 64
6. Germany: 55
7. Argentina: 35
8. Switzerland: 34
9. Italy: 32
10. Sweden: 30

ABSTRACTS & CASES

Accepted Abstracts

CMR Open-Source Software: 5

Congenital Track: 12

Early Career Award Basic Science: 6
Early Career Award Clinical: 6
Early Career Award Congential: 6
Early Career Award Translation: 6

ISMRM-SCMR Preconference Workshop: 3

MICCAI Challenge: 3

Oral Abstracts Basic Science: 30
Oral Abstracts Clinical: 24
Oral Abstracts Translation: 18

Oral Case Cardiac Masses: 6
Oral Case Ischemic Heart Disease: 6
Oral Case Non-Ischemic Cardiomyopathy: 6
Oral Case Systemic and Inflammatory: 6

Quick Fire: 165

Rapid Fire: 327

Technologist Track Abstract: 6
Technologist Track Case: 6

Unmet Clinical Needs: 2

Accepted Countries

Argentina: 21
Australia: 2
Azerbaijan: 1
Belgium: 2
Brazil: 36
Bulgaria: 1
Canada: 27
Chile: 11
China: 37
(People's Republic)
Colombia: 5
Cuba: 1
Ecuador: 1
Egypt: 7
Finland: 1
France: 21
Germany: 35
Hong Kong: 1
India: 18
Iran: 1
Italy: 27
Japan: 10
Lebanon: 1
Malaysia: 1
Mexico: 15
Netherlands: 19
New Zealand: 2
Oman: 3
Panama: 1
Peru: 4
Philippines: 1
Portugal: 5
Romania: 1
Russia: 3
Singapore: 1
South Africa: 3
Spain: 2
Sweden: 21
Switzerland: 13
Taiwan: 3
(Republic of China)
Thailand: 3
Turkey: 2
United Arab Emirates: 1
United Kingdom: 51
United States: 233

Abstracts and Cases Submitted
794

Countries with Accepted Abstracts
44

Accepted Abstracts and Cases
655

Late-Breaking Abstracts: 6

TRAVEL GRANT AWARDS

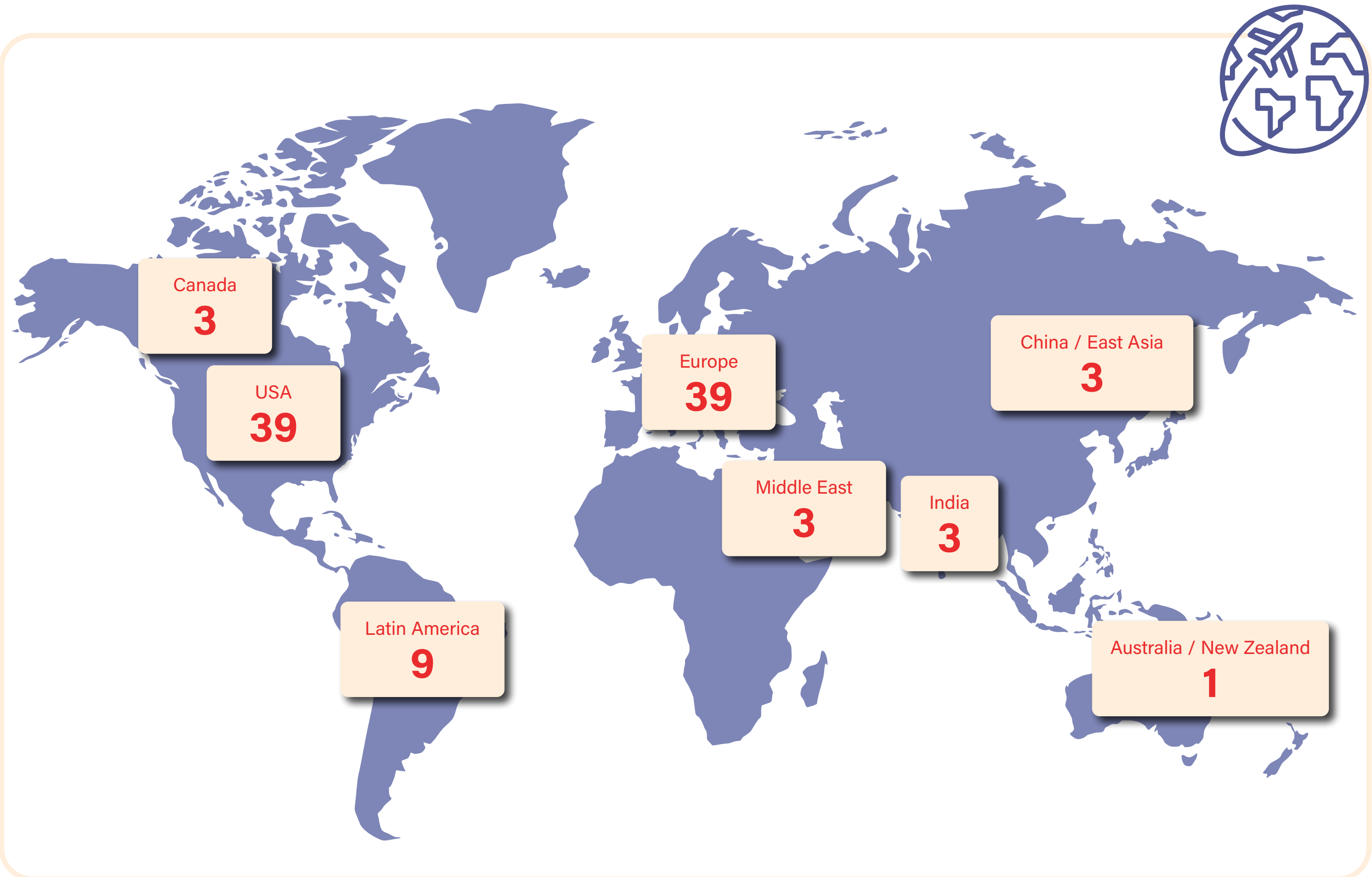

Applications Received
103

Travel Grants Awarded
61

Registered & Attended SCMR 2026
53

INVITED FACULTY MEMBERS

Representation by **Speaker**, **Moderator**, and **Panelist** Roles

774
Roles in Total
included in the program

IN-PERSON	
Argentina	12
Australia	8
Belgium	3
Brazil	56
Canada	25
Chile	13
China (People's Republic)	9
Colombia	3
Egypt	1
France	4
Germany	29
Greece	2
Hungary	1
India	4
Italy	31
Japan	3
Malaysia	1
Mexico	9
Netherlands	24
New Zealand	2
Norway	1
Peru	5
Portugal	9
South Africa	4
Sweden	10
Switzerland	11
United Kingdom	113
United States	381

TOP COUNTRIES

1. United States: 381
2. United Kingdom: 113
3. Brazil: 56
4. Italy: 31
5. Germany: 29

TOTAL COUNTRIES: 28

Speciality	
Cardiologist	455
Radiologist	104
Scientist	123
Technologist	24
Did Not Respond	68

Gender	
Female	253
Male	511
Prefer not to say	10

SCMR 2026 HIGHLIGHTS

6 Preconference Courses/Workshops

80 Scientific Sessions (excluding pre-conference sessions)

34 Sessions with live translation

8 Hands-On Advanced Sponsored Workshops (14 sessions in total) all reaching full capacity

8 Innovation Track Sessions

68 Countries Represented

44 Countries with Accepted Abstracts/Cases

61 Travel Grants Awarded

300 Participants from Latin America (the highest representation in SCMR history)

45 LATAM Societies supporting SCMR 2026

JOINT SESSIONS

8 joint sessions and 1 pre-conference

13 Sister Societies

European Society of Cardiovascular Radiology

DIC

Sociedade Brasileira de Cardiologia – SBC 1943

MICCAI

SMRA

SCCT – Society of Cardiovascular Computed Tomography

SPR – Sociedade Paulista de Radiologia e Diagnóstico por Imagem

IC-OS – International Cardio-Oncology Society

Heart Rhythm Society℠

NASCI – North American Society for Cardiovascular Imaging

EACVI – European Association of Cardiovascular Imaging

International Society for ISMRM – Magnetic Resonance in Medicine

ASCI – Asian Society of Cardiovascular Imaging

ASNC – American Society of Nuclear Cardiology

SYMPOSIUMS

THURSDAY 5 FEBRUARY

vista

Breakfast Symposium
Taking CMR Beyond Human Limit
Daniel Hawkins - CEO Vista AI
Mohamad Khattab, DO

BAYER

Lunch Symposium
CMR for Cardiovascular Disease: The Time is Now!
Jeanette Schulz-Menger, MD
James C. Moon, MD
Laura C. Dragonetti, MD
Raymond Y. Kwong, MD, MPH, FSCMR
Tim Leiner, MD, PhD

circle CARDIOVASCULAR IMAGING

Lunch Symposium
Imaging Intelligently: Optimizing Patient Care with MR and CT
Joao Cavalcante, MD
Tilman Emrich, MD, FSCMR

FRIDAY 6 FEBRUARY

PHILIPS

Breakfast Symposium
Quantitative Perfusion: Why and how we do it
Sven Plein, MD, PhD
Automatic Cardiac Planning using SmartHeart: Insights into clinical practice
Julian A. Luetkens, MD

JENAVALVE

Lunch Symposium
Redefining Aortic Regurgitation: Diagnostic Challenges, Advanced Imaging and Emergining Therapies
Purvi Parwani, MBBS, Fellow
CMR: A Crucial Componenet to Accurate AR Assessment
Dipan J. Shah, MD
Unmet needs for AT Treatment Evidence for Decicated Devices and ARTIST Trial
Cleverson Zukowski, MD, PhD, FESC
ALIGN-AR post-hoc CT analysis and ARTIST Imaging Substudy
Joao Cavalcante, MD

SIEMENS Healthineers

Lunch Symposium
Cardiac MRI at 0.55T: Introducing MAGNETOM Free.XL
Oskar Isay
Is Low-field the future of Cardiac MRI?
Amedeo Chiribiri, PhD, MB, FSCMR
Cardiac MRI in the Era of Precision Oncology: Tracking Cardiotoxicity from Targeted Therapies to Immunotherapies
Marco Francone, MD, PhD

SATURDAY 7 FEBRUARY

GE HealthCare

Breakfast Symposium
Rapid pediatric cardiac MRI with Sonic DL – The Power to Explore Further
Melany B. Atkins, MD
Michael P. DiLorenzo, MD, MSc

SYMPOSIUMS

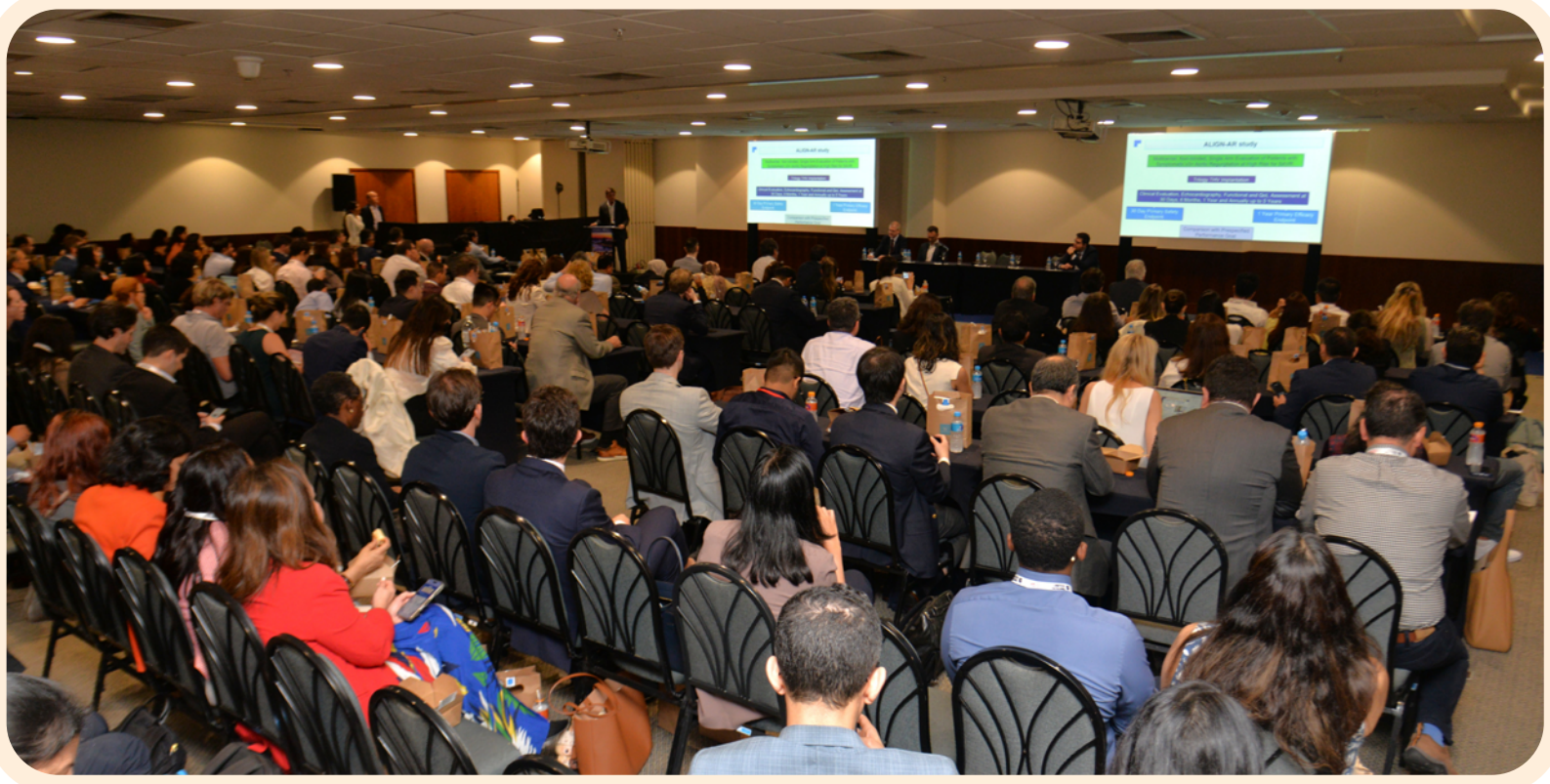
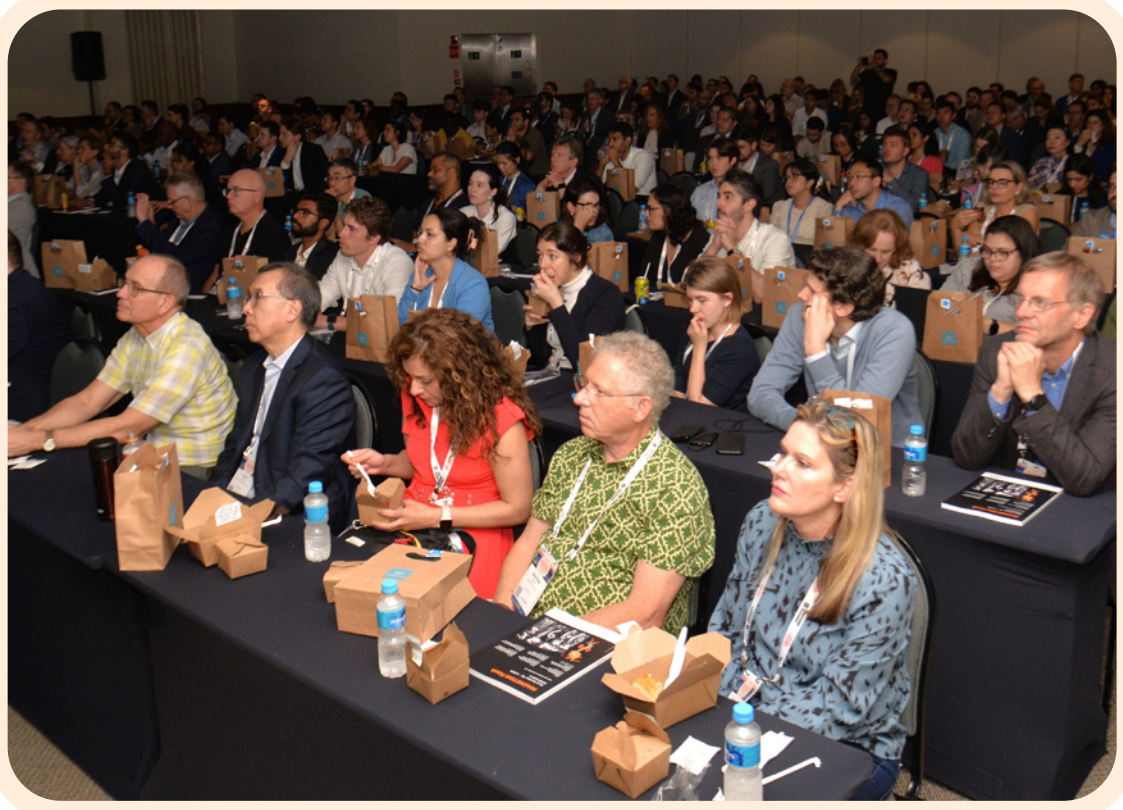
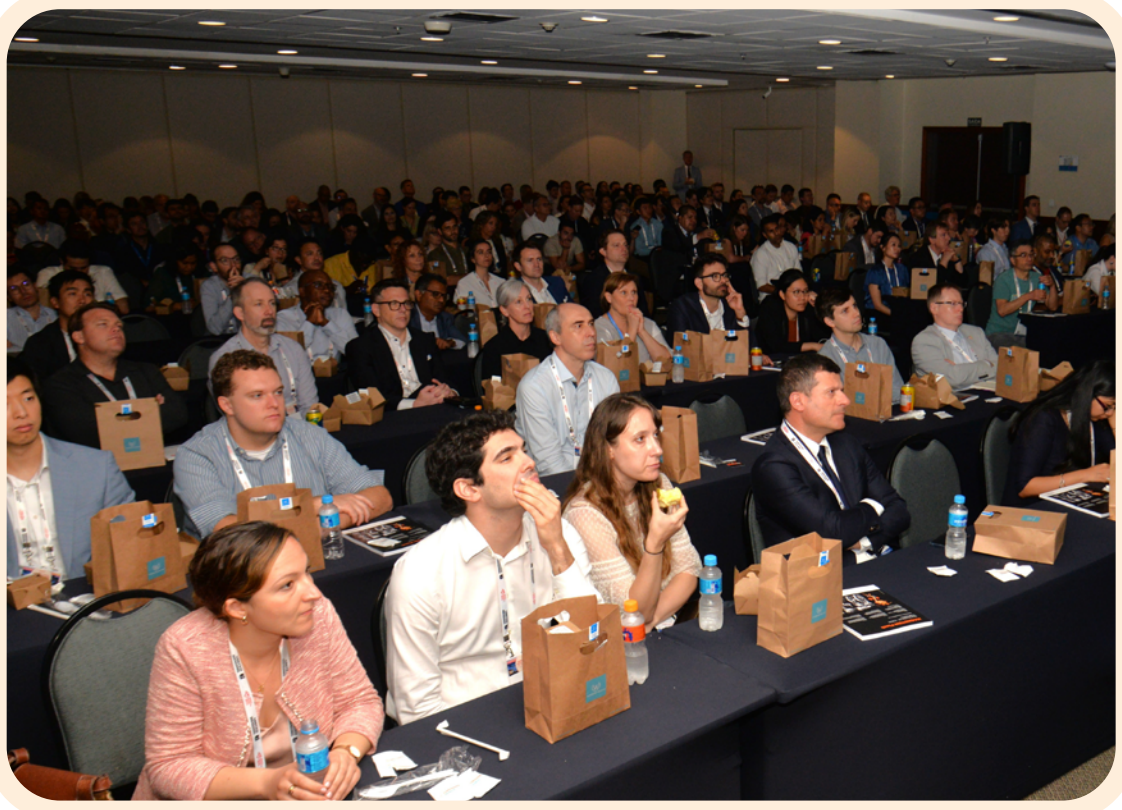
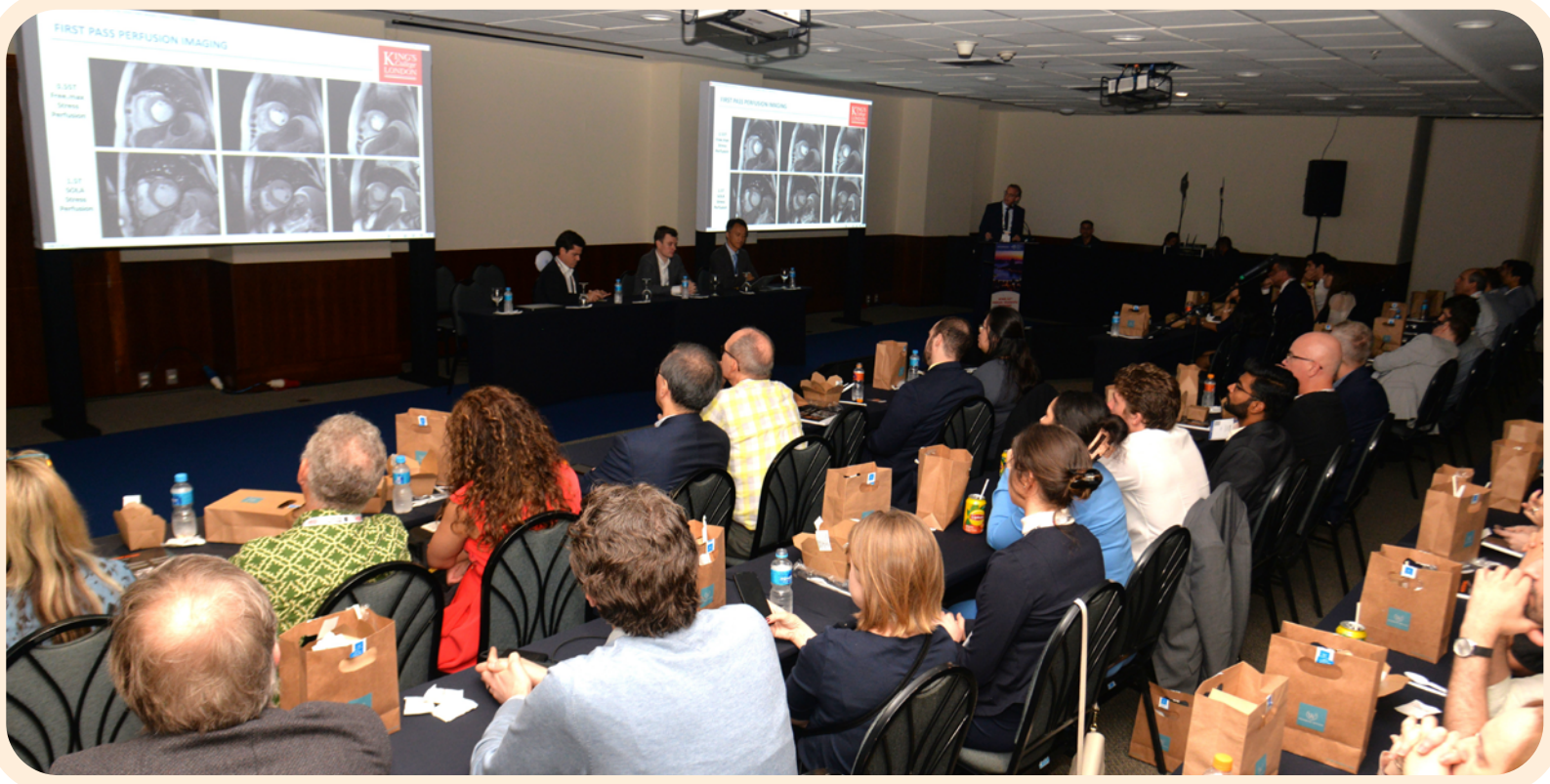
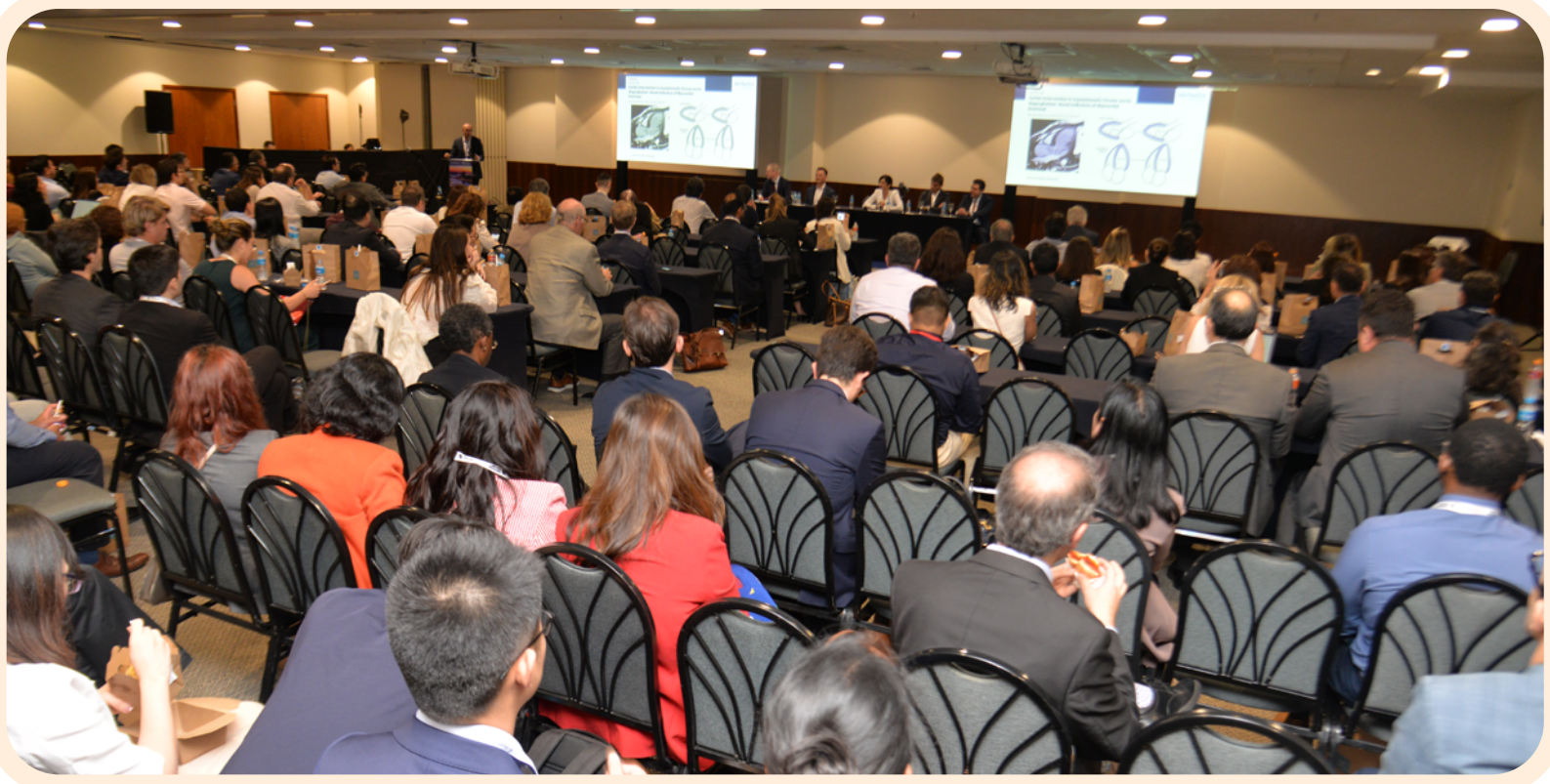
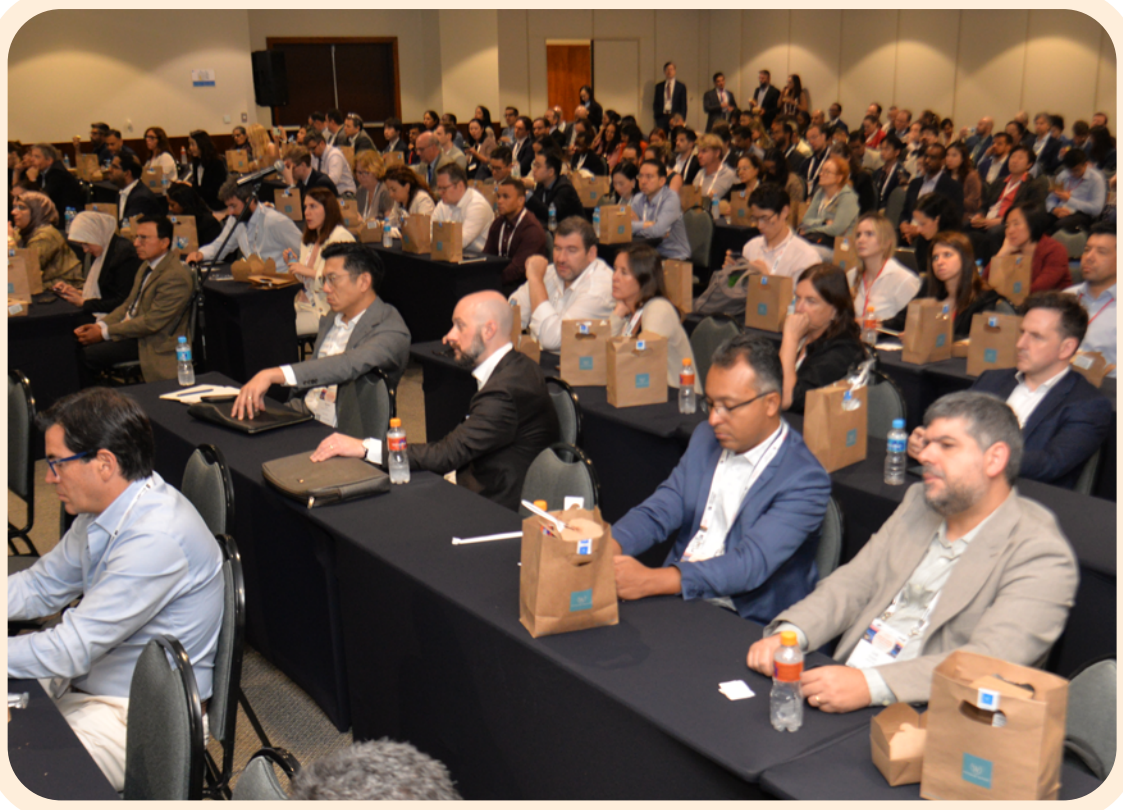
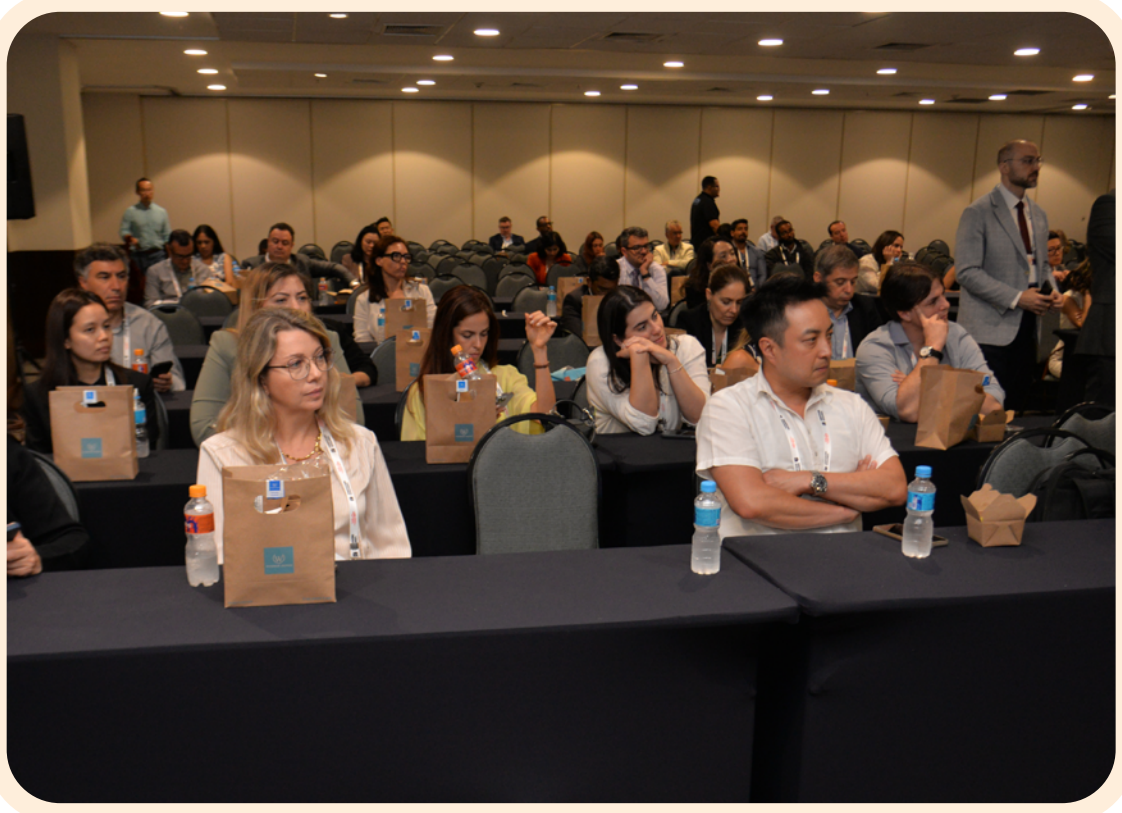
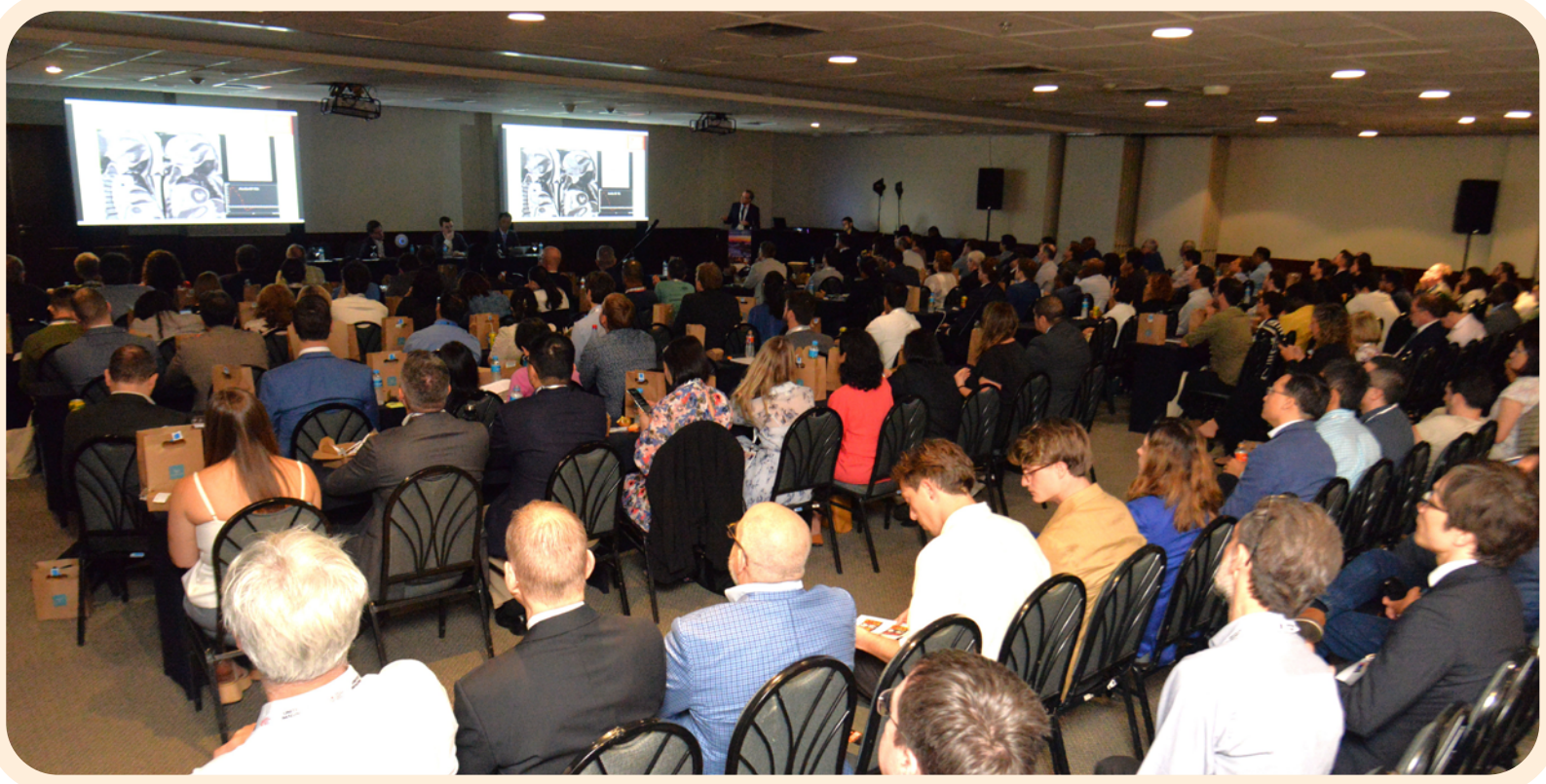

SPONSORED HANDS-ON SESSIONS

With the Support of Our Industry Partners

NEOSOFT · circle CARDIOVASCULAR IMAGING · Medis MEDICAL IMAGING · PIE MEDICAL IMAGING

8 HANDS-ON SESSIONS

Session 1 (PIE MEDICAL IMAGING)
Introduction to 4D Flow

Gaston Vogel, MSc - Product Manager, Pie Medical Imaging, Maastricht, Limburg, Netherlands
Malenka M. Bissell, MD, PhD, FSCMR - Clinical lecturer, University of Leeds, Leeds, England, United Kingdom

Session 2 (circle CARDIOVASCULAR IMAGING)
Quantifying Flow: A Hands-On Workshop on 4D Flow Data Analysis

Christopher J. Francois, MD - Professor, Mayo Clinic, Rochester, Minnesota, United States

Session 3 (NEOSOFT)
Systolic and Diastolic Heart Failure

Steven Wolff, MD, PhD - Owner, NeoSoft, LLC, New York, New York, United States
Tommaso D'Angelo, MD, PhD - Associate Professor of Radiology, Università degli Studi di Messina, Messina, Sicilia, Italy

Session 4 (NEOSOFT)
In 2D and 4D Flow We Trust!

Steven Wolff, MD, PhD - Owner, NeoSoft, LLC, New York, New York, United States

Session 5 (Medis MEDICAL IMAGING)
Mastering Real-World CMR with Advanced Post-Processing

Sara E. Ramirez Flores, MD - Coordinator of Advanced Cardiac Imaging Unit (Hospital Central FAP) / Coordinator of Cardiovascular Imaging Unit (Clínica Internacional), Hospital Central Fuerza Aérea del Perú / Clínica Internacional, Lima, Lima, Peru
Erasmo De La Pena-Almaguer, MD - Director, Cardiovascular Service Line and CV Imaging Dpt, Tecnologico de Monterrey, Monterrey, Nuevo Leon, Mexico
Lucas Gutierrez, MD - Chief Cardiac MRI, Penta Medicina Cardiovascular, Lujan de Cuyo, Mendoza, Argentina

Session 6 (PIE MEDICAL IMAGING)
Advanced 4D Flow: A Selection of Cases

Malenka M. Bissell, MD, PhD, FSCMR - Clinical lecturer, University of Leeds, Leeds, England, United Kingdom
Gaston Vogel, MSc - Product Manager, Pie Medical Imaging, Maastricht, Limburg, Netherlands

Session 7 (circle CARDIOVASCULAR IMAGING)
The Future of Stress Testing: Precision Quantitative Perfusion by CMR

Sven Plein, MD, PhD - Professor of Cardiology, Leeds Institute of Cardiovascular and Metabolic Medicine, Leeds, England, United Kingdom

Session 8 (Medis MEDICAL IMAGING)
Show Me the Case: Mastering Real-World CMR with Advanced Post-Processing

Robin Nijveldt, MD, PhD - Prof. Dr. Radboud University Medical Center, Nijmegen, Gelderland, Netherlands
Alexander Schulz, MD - Dr. Harvard Medical School / BIDMC Berlin, Berlin, Germany

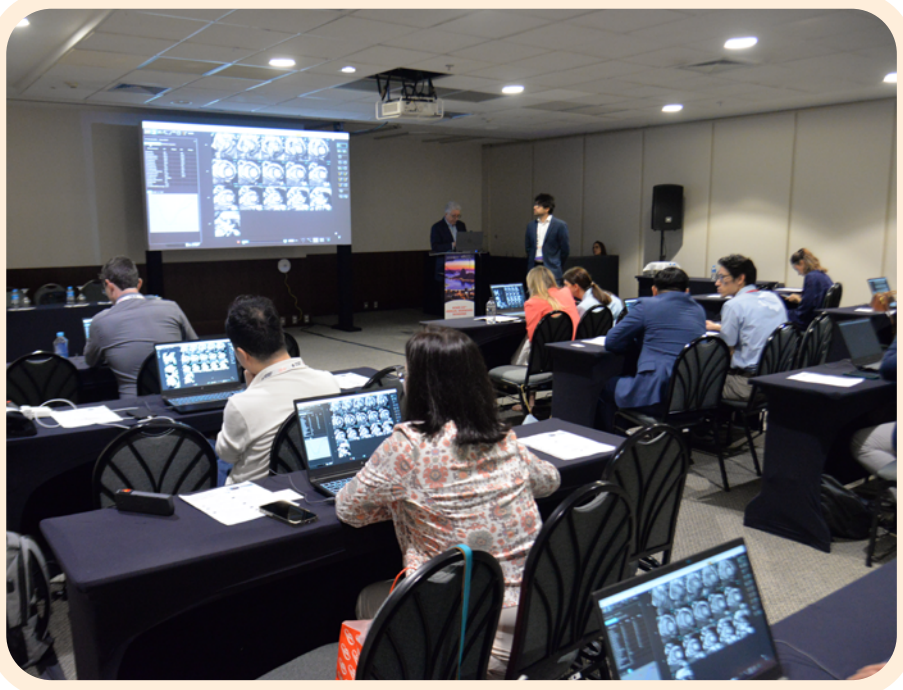

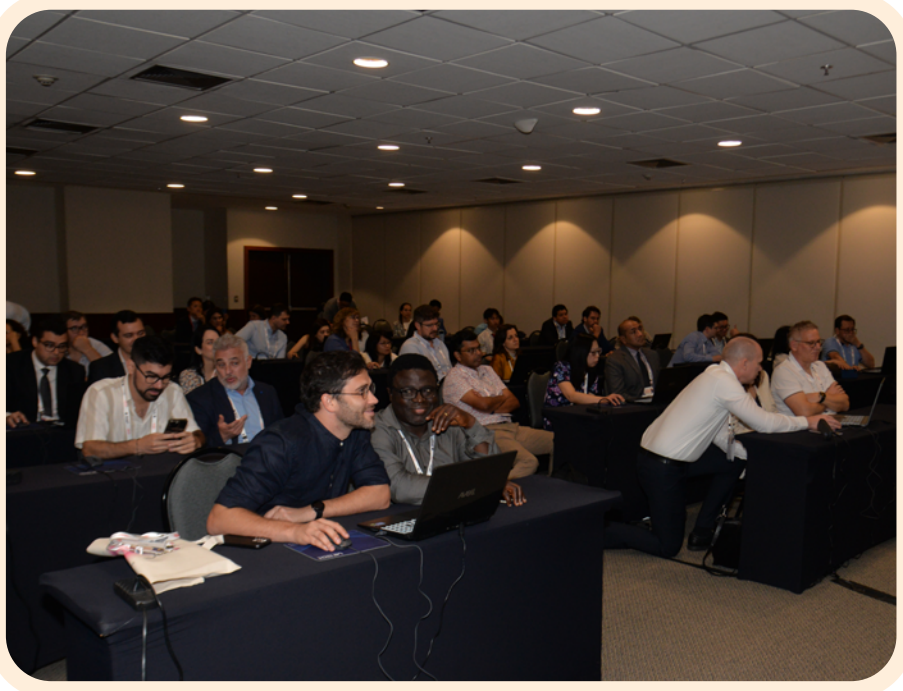

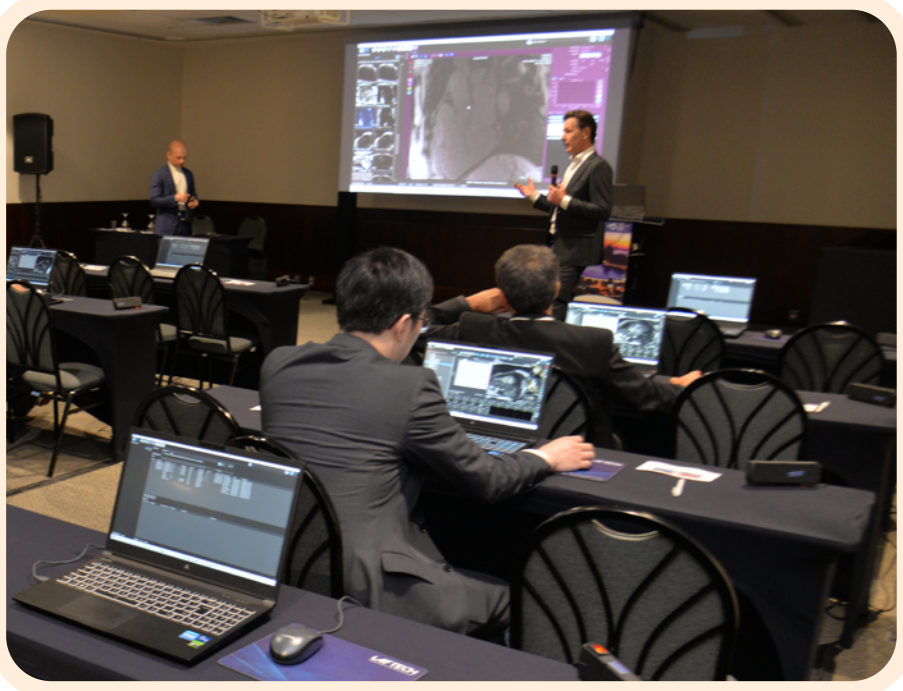

COFFEE BREAK SPONSORSHIP

With the Support of Our Industry Partners

heartvue.ai | imricor | MYCARDIUM PRECISION CARDIAC DIAGNOSTICS

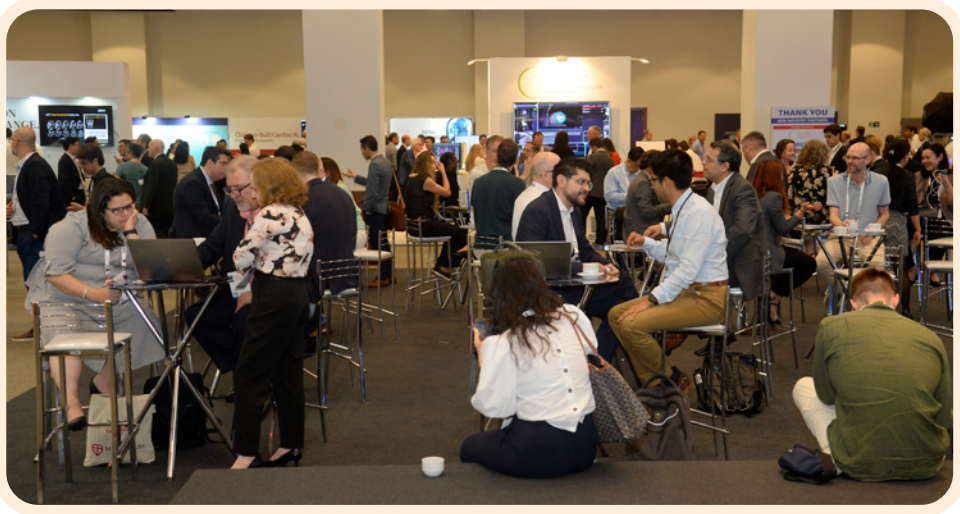

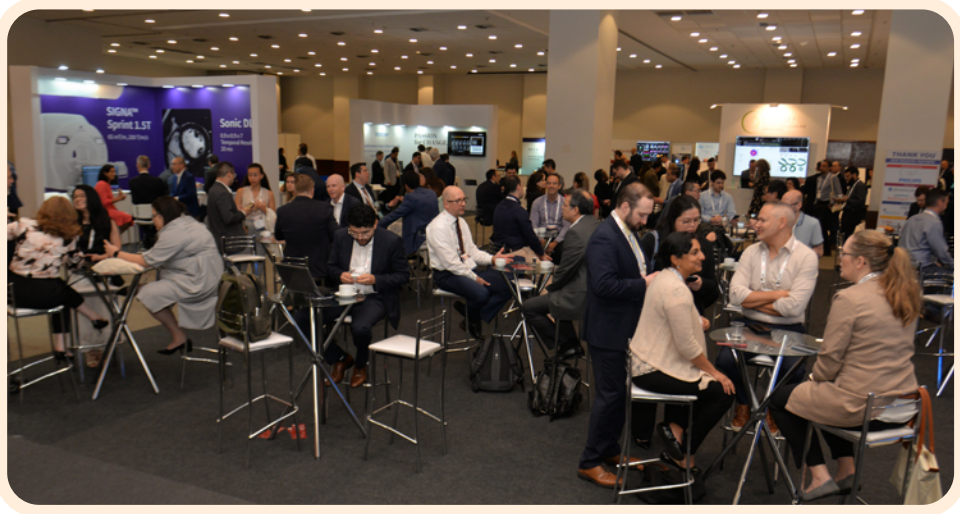

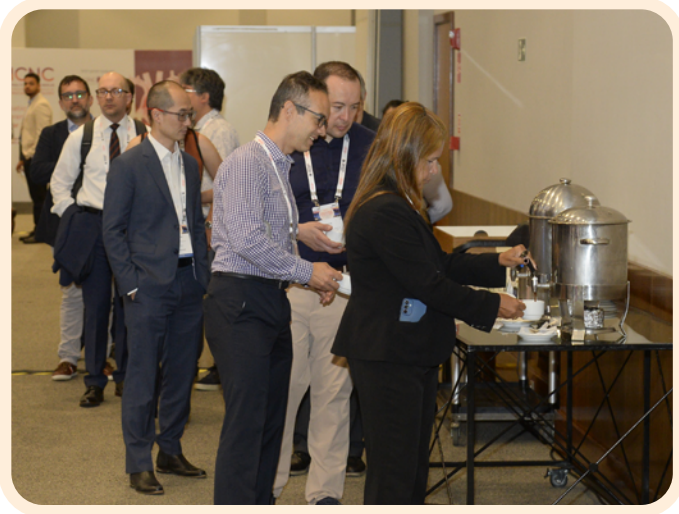

THAN YOU TO OUR INDUSTRY PARTNERS

Double Diamond

circle CARDIOVASCULAR IMAGING | PHILIPS

Diamond

BAYER | JENAVALVE | SIEMENS Healthineers

Platinum

GE HealthCare | NEOSOFT | vista

Gold

Canon CANON MEDICAL | Medis MEDICAL IMAGING | PIE MEDICAL IMAGING | UNITED IMAGING

Silver

imricor | heartvue.ai | MYCARDIUM PRECISION CARDIAC DIAGNOSTICS

Bronze

BRACCO LIFE FROM INSIDE | ziosoft | CARDIO FLOW DESIGN | Adas3D Medical | ALTA DIAGNÓSTICOS | CASIS Cardiac Simulation & Imaging Software | NORTHH MEDICAL | AI4CMR | Realize MEDICAL

Tabletops

stratasys | HELICAL ANALYTICS | HEXAMEDICAL TAKING CARE OF YOUR EQUIPMENT | Careverse

KEYNOTE SPEAKERS

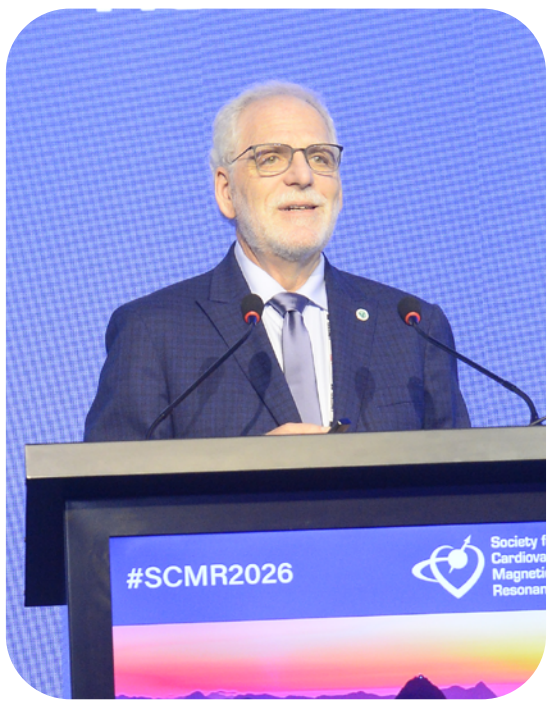

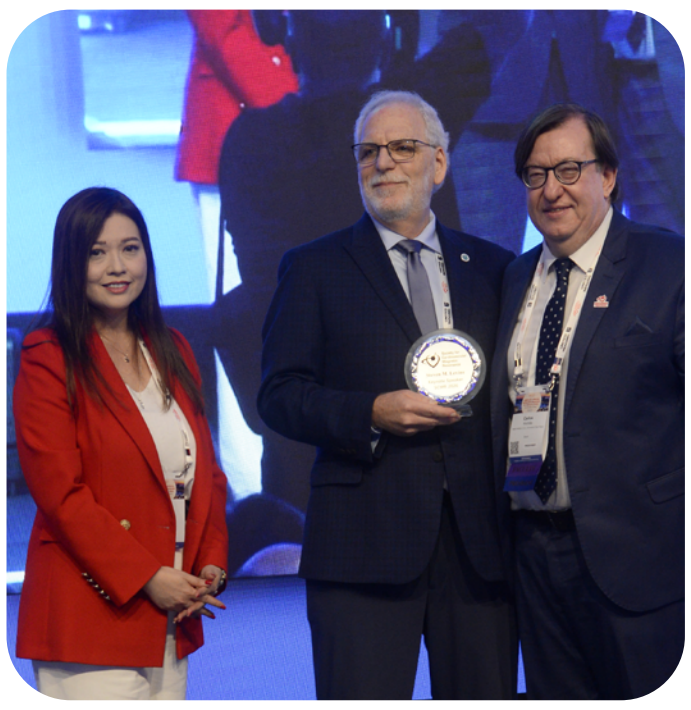

Steve Levine, PhD

Sr. Director, Virtual Human Twin & Founder, Living Heart Project
San Diego County, California, United States
"The Living Heart: How Digital Twins Are Transforming Cardiovascular Medicine"

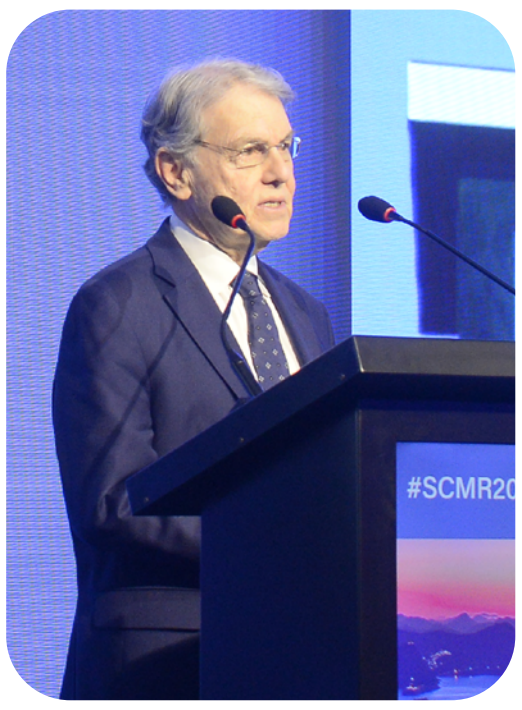
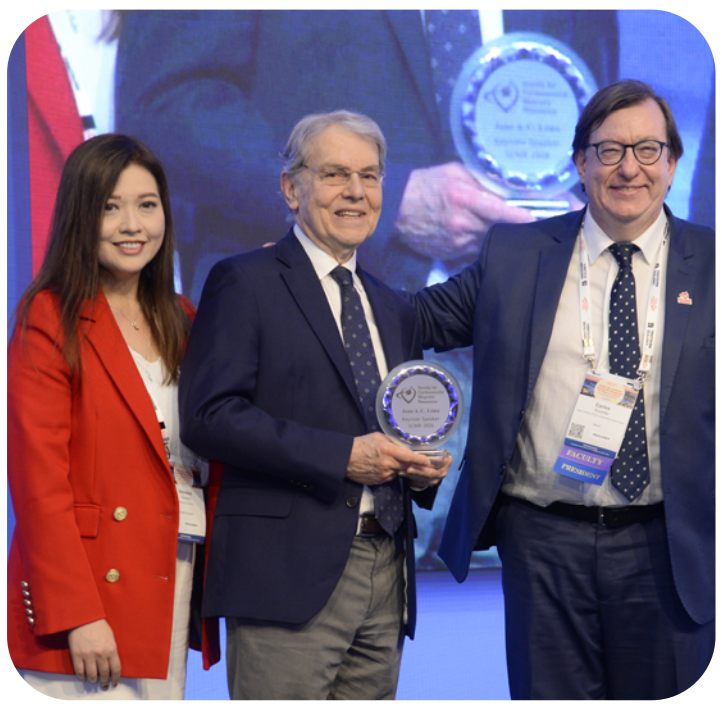

Joao A. Lima, MD

Professor - The Johns Hopkins University
Baltimore, Maryland, United States
"CMR and SCMR Journey: Science, Discovery, and Global Health"

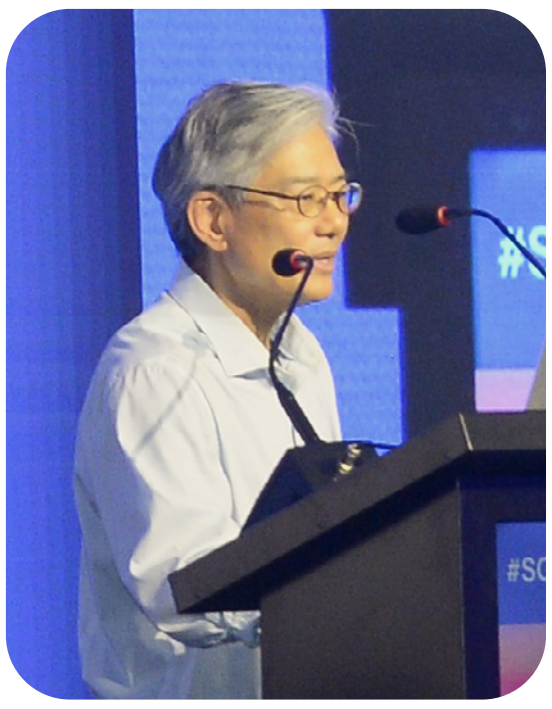
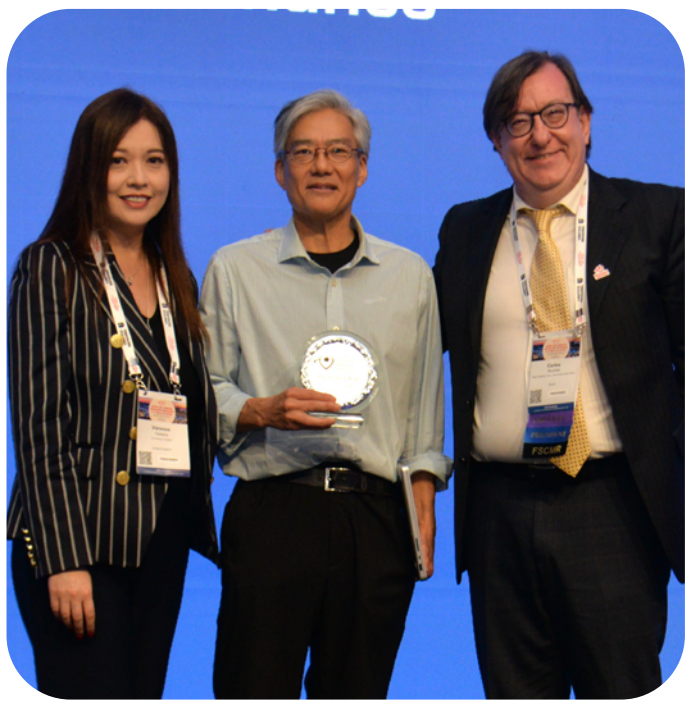

Raymond Kim, MD

Professor - Duke Cardiovascular Magnetic Resonance Center
Durham, North Carolina, United States
"What is the Next Paradigm Shift for CMR"

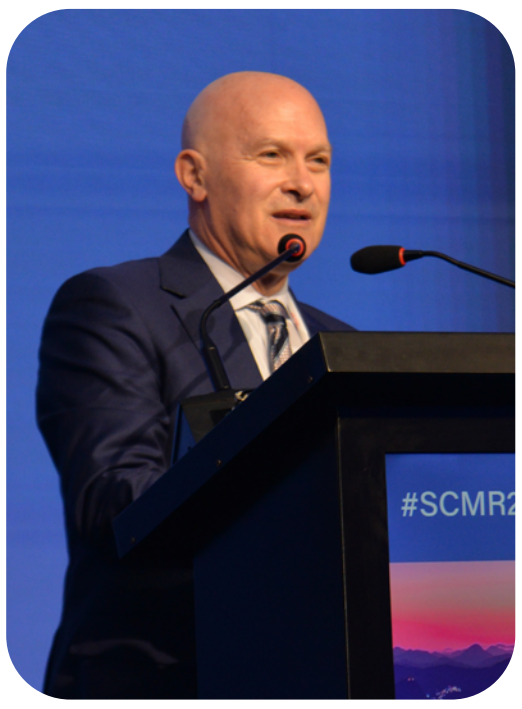
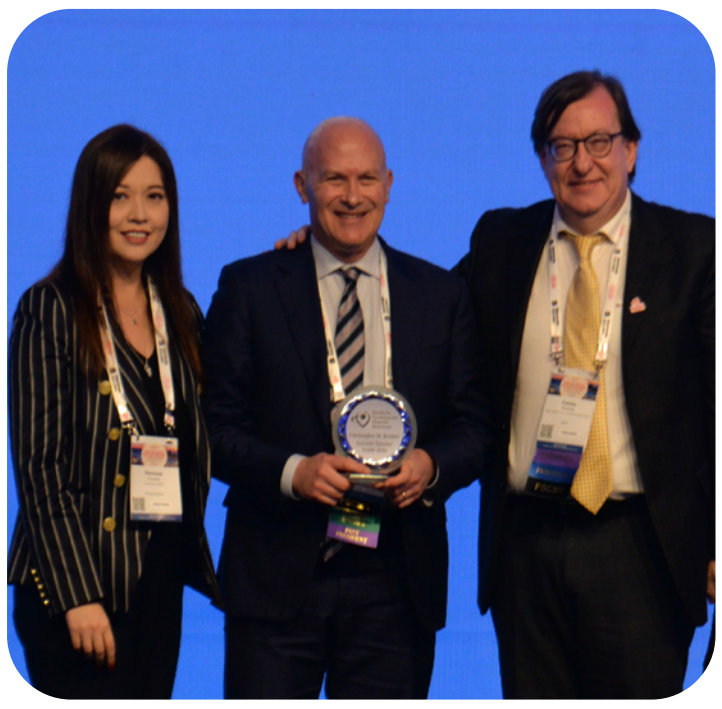

Christopher M. Kramer, MD

Professor - University of Virginia Health
Charlottesville, Virginia, United States
"CMR and the Future of Global Cardiovascular Health"

GOLD MEDAL 2025 TALKS

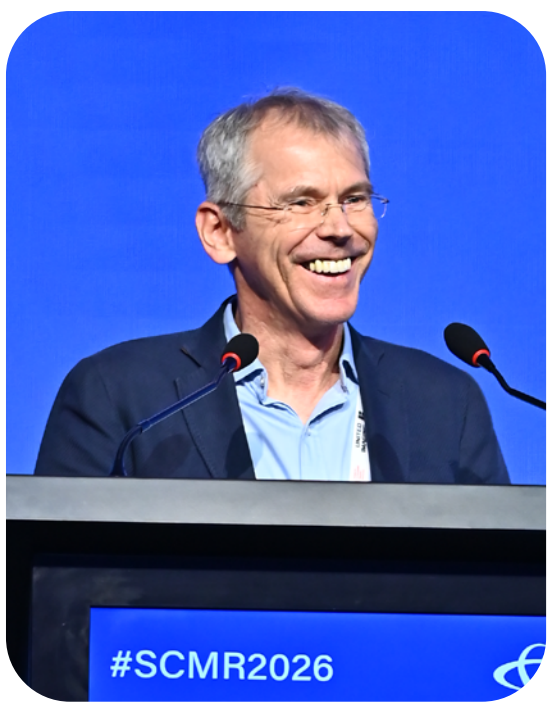

Rene M. Botnar, PhD

Professor of Cardiovascular Imaging
Institute for Biological and Medical Engineering,
Pontificia Universidad Católica de Chile
Santiago de Chile, Region Metropolitana, Chile
"Coronary MR: Seeing Beyond the Lumen"

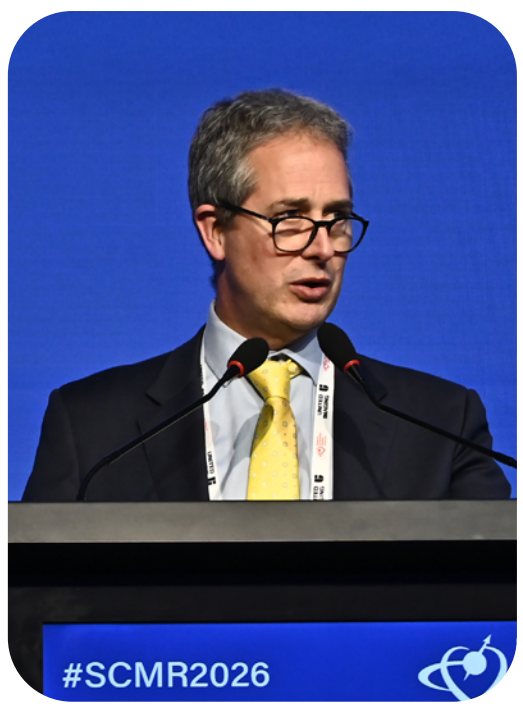

Steffen E. Petersen, MD, PhD, MSc, MPH, FSCMR

Professor of Cardiovascular Medicine
Queen Mary University of London
London, England, United Kingdom
"The Privilege of Scale: the Stories Inside the CMR Data"

2026 SCMR AWARDS

GOLD MEDAL

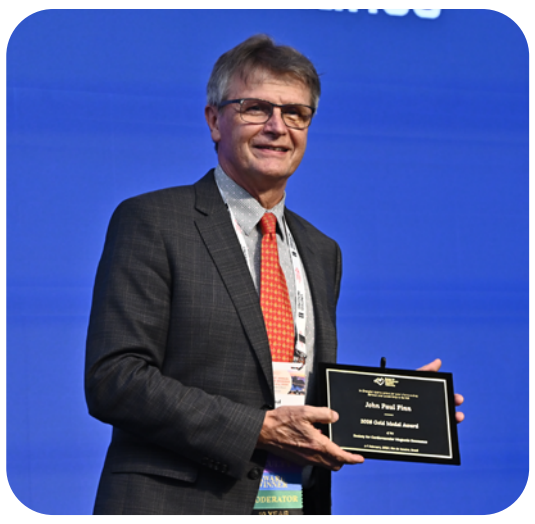

John Paul Finn, MD

Diagnostic Cardiovascular Imaging Laboratory, Department of Radiological Sciences,
David Geffen School of Medicine at UCLA, Los Angeles, CA, USA
Physics and Biology in Medicine Graduate Program, University of California,
Los Angeles, CA, USA

The Gold Medal is the Society's highest award of distinction and given only to an individual for distinguished and extraordinary contribution to innovating and advancing the field of Cardiovascular Magnetic Resonance.

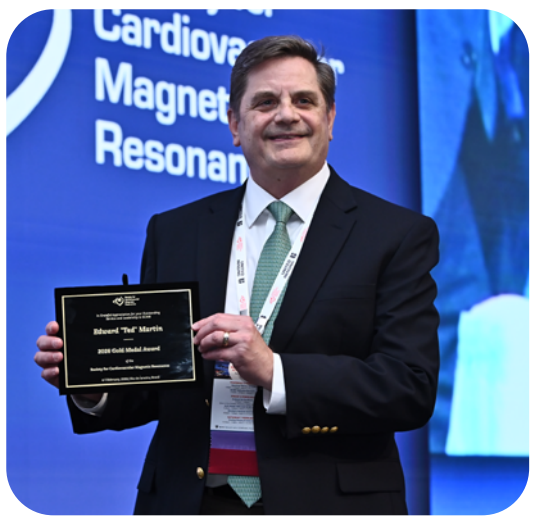

Edward “Ted” Martin, MD, MSCMR, FSCMR

Oklahoma Heart Institute, Tulsa, OK, USA

The Gold Medal is the Society's highest award of distinction and given only to an individual for distinguished and extraordinary contribution to innovating and advancing the field of Cardiovascular Magnetic Resonance.

SCMR ADVOCACY AWARD

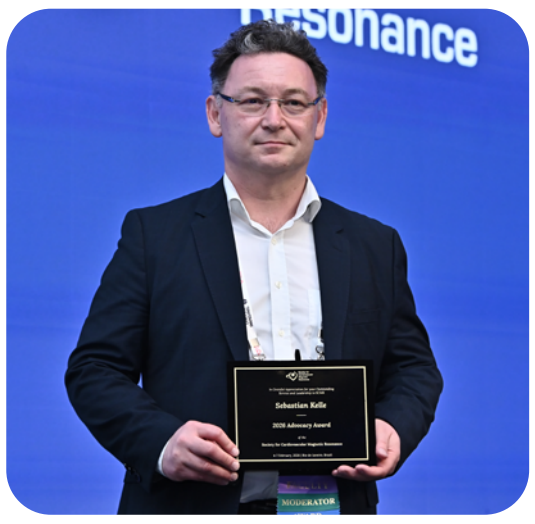

Sebastian Kelle, MD, FESC, FSCMR

German Heart Center of the Charité University Medicine - Berlin,Germany

The SCMR Advocacy award recognizes members who have made exemplary contributions to the field of CMR and the Society in national and international advocacy. Candidates will have made major contributions of engagement in public policy issues and concerns affecting CMR professionals and our patients around the world, including activities to prioritize public policy issues and concerns, identify those that SCMR should focus its attention and resources, and contribute to raising awareness about the role of CMR for patient care.

SCMR EDUCATION AWARD

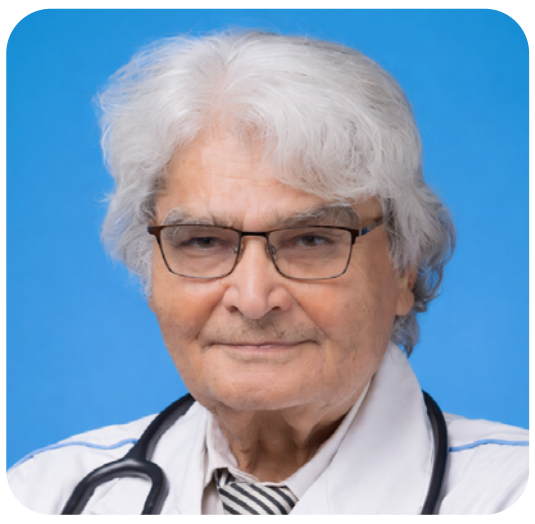

Omprakash Tavri, MD, DMRE, FICR

Asian Heart Institute - Mumbai, India

The SCMR Education award recognizes members who have made exemplary contributions to the field of CMR and the Society in education and training. Candidates for this award will have a developed or led educational and training efforts in CMR, including in-person and online learning, developed new educational materials addressing current SCMR needs in the most effective manner, developed CMR or SCMR training guidelines or undertaken other major activities in the and training relevant to the CMR community.

SCMR TECHNOLOGIST AWARD

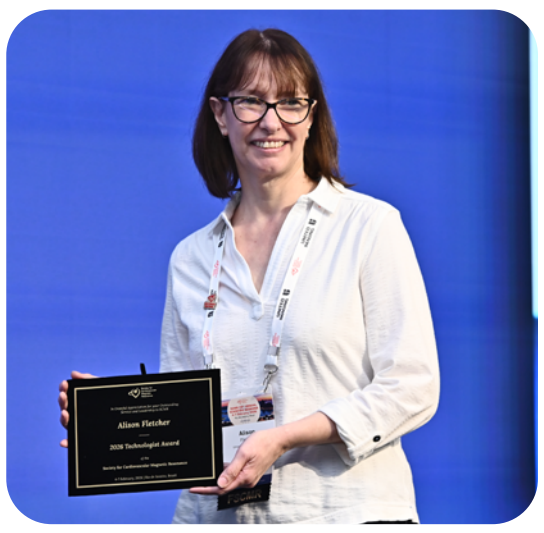

Alison Fletcher, FSCMR

Papworth Hospital - Cambridge, UK

The SCMR Technologist Award recognizes technologists who have made exemplary contributions to the Society and/or field of CMR. Candidates for this award should demonstrate a track record of distinguished service to the Society in the area of technologist education, training, practice development, mentoring, or research.

MASTER OF SCMR (MSCMR)

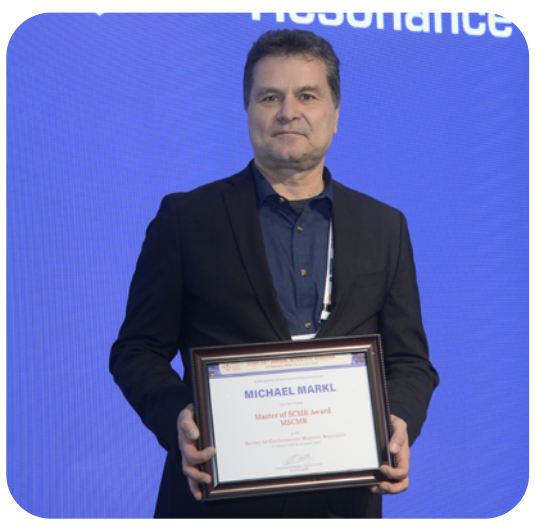

Michael Markl, PhD, FSCMR

Departments of Radiology and Biomedical Engineering, Northwestern University - Chicago, USA

The Master of the Society for Cardiovascular Magnetic Resonance (MSCMR) credential is awarded to individuals who have a track record of distinguished service to the Society and have demonstrated outstanding accomplishments in multiple domains including scientific, educational, clinical, or advocacy. Members who fulfill published criteria can be nominated by the Board of Trustees and the Executive Committee. Awards are made once a year and given out at the Annual Scientific Sessions.

EARLY CAREER AWARDS

Translational Science

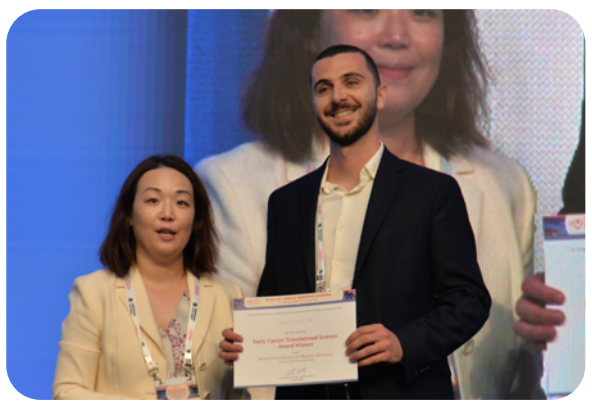

Thomas Coudert, PhD
Postdoctoral Research FellowUniversity of California, Los Angeles
Los Angeles, California, United States
"Dynamic Fractional Myocardial Blood Volume Mapping Using Ferumoxytol-Enhanced MR Multitasking"

Basic Science

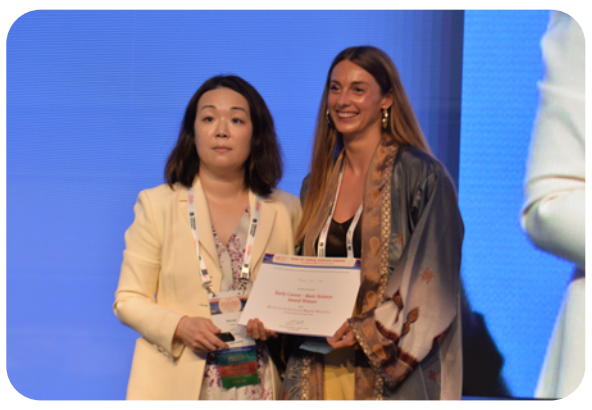

Pauline Gut, PhD
University Hospital (CHUV) and University of Lausanne (UNIL)Lausanne, Vaud, Switzerland
"Wideband myocardial T2 mapping in patients with implantable cardiac devices at 1.5 T"

Congenital Science

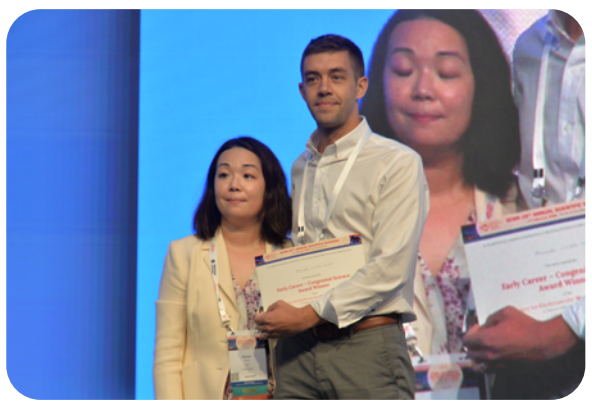

Alexander Kiener, MD, MPH
Post-Doctoral Clinical FellowBaylor College of Medicine / Texas Children's Hospital
Houston, Texas, United States
"Prognostic Value of Stress Perfusion CMR in Kawasaki Disease with Coronary Artery Aneurysms"

Clinical Science

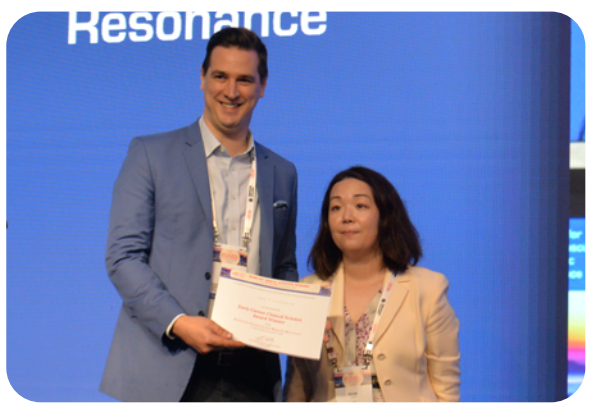

Simon J. Littlewood, MD
Clinical Research FellowKing's College London, London, England, United Kingdom
"Combined Coronary Vessel Lumen and Plaque Assessment Using iT2prep-BOOST:
A Prospective Study in Patients with Stable Chest Pain"

TECHNOLOGIST AWARDS

Best Abstract

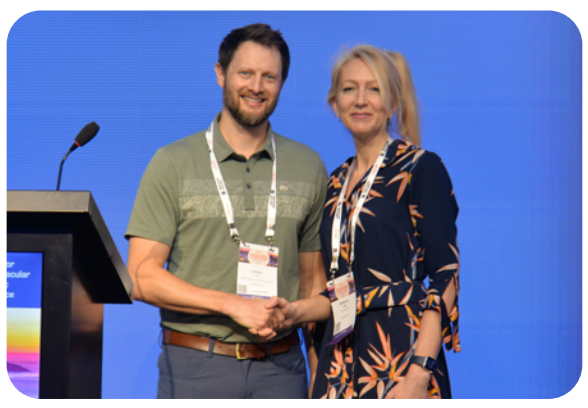

Jordan C. Taylor
NIH
"Free-breathing isotropic 3D whole-heart imaging vs 2D cartesian imaging:
A comparison of left ventricular volumetry"

Best Case

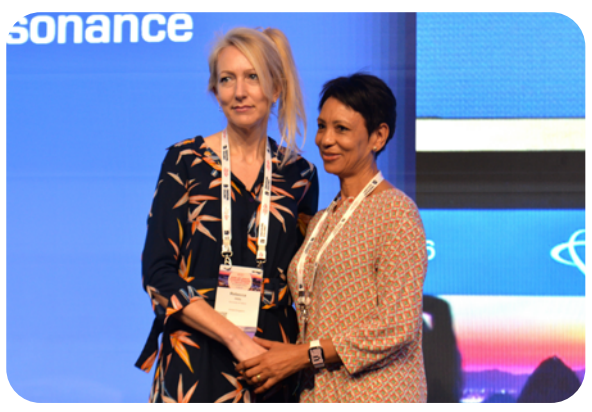

Petronella Samuels, MSc
University of Cape Town
"Application of Cardiac Proton Magnetic Resonance Spectroscopy to Myocardial Steatosis in Women with ART: a Case Report"

ADDITIONAL SCMR 2026 AWARDS

Journal of Cardiovascular Magnetic Resonance

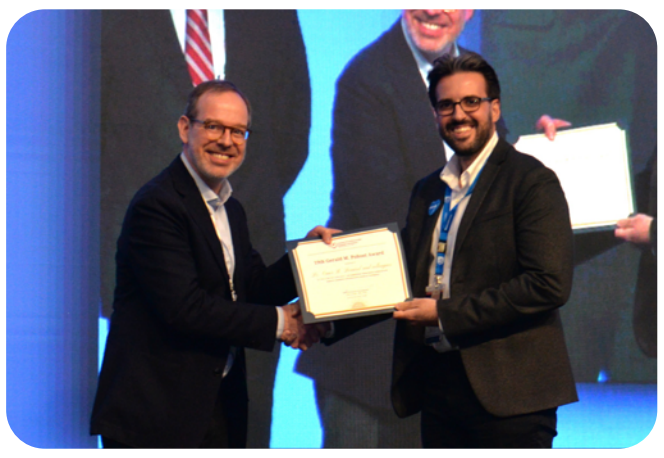

19th Gerald M. Pohost Award

JCMR Award Winner

Omer Burak Demirel, PhD, and colleagues

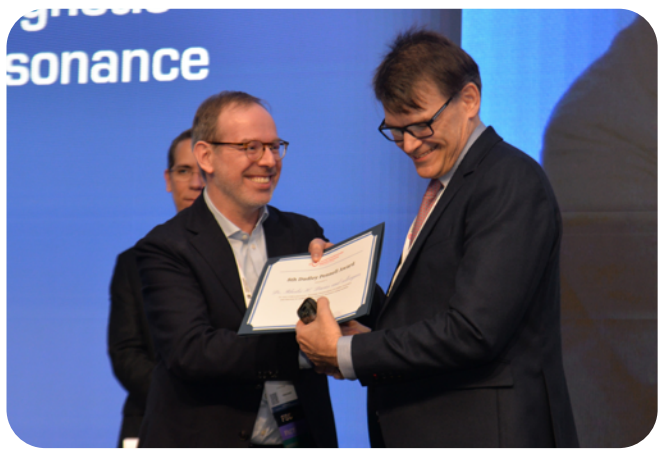

8th Dudley Pennell Award

JCMR Award Winner

Rhodri H. Davies, PhD, and colleagues

James C. Moon (last author) collecting the award on stage

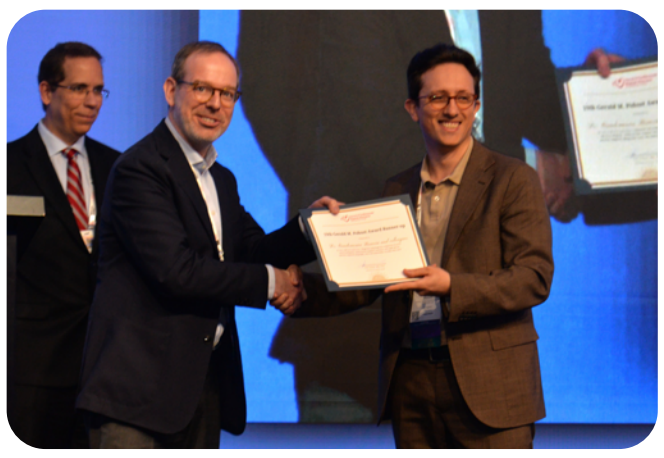

19th Gerald M. Pohost Award

JCMR Award Runner-up

Giandomenico Bisaccia, MD, PhD, and colleagues

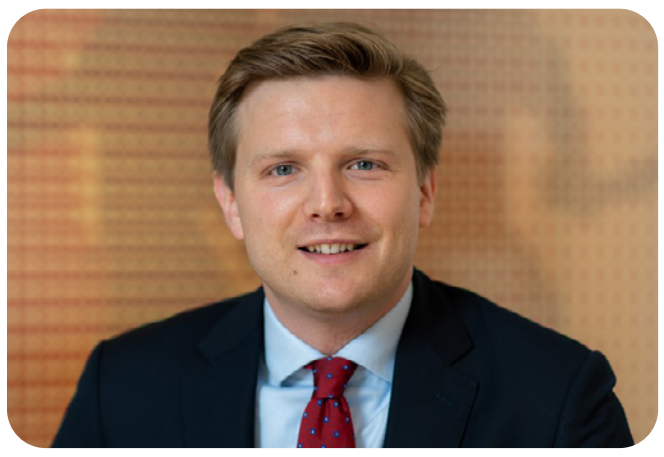

8th Dudley Pennell Award

JCMR Award Runner-up

Friso M. Rijnberg, MD, PhD, and colleagues

2026 Best Case of SCMR

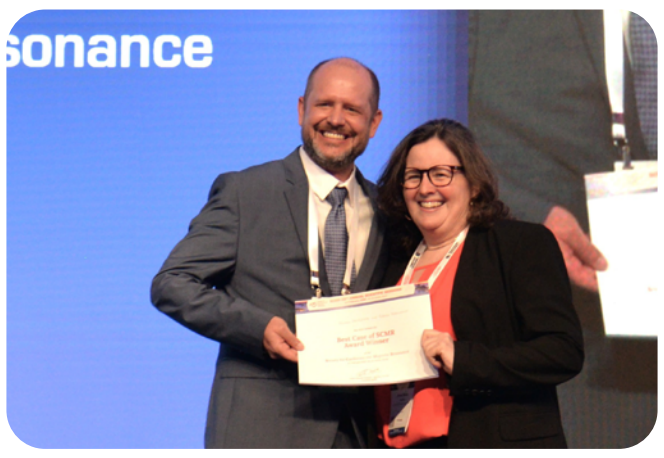

Karina Javalkar, MD and Rebecca S. Beroukhim, MD

"Biventricular Repair of Complex Congenital Left Ventricular Aneurysm"

(Anne-Marie Valente collecting the award on stage)

SCMR 2026 Best Image Competition

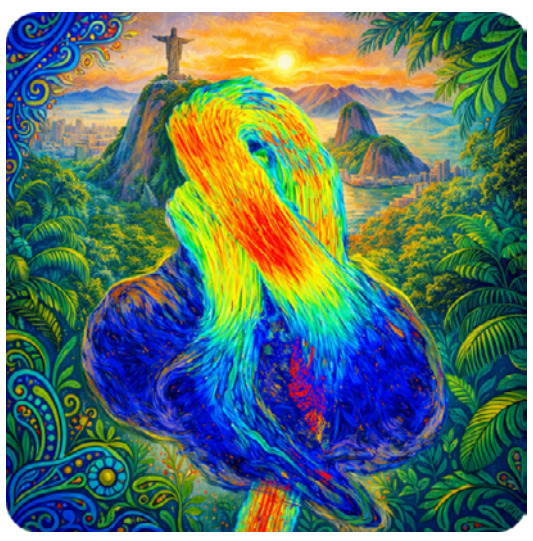

Winner

"Flows of Life: Resonance of Deep Brazil"

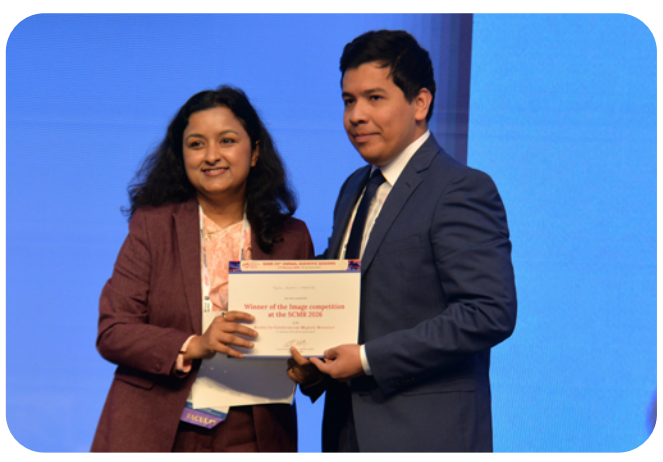

Martín Alanís-Naranjo

SCMR 2026 Best Social Media Post

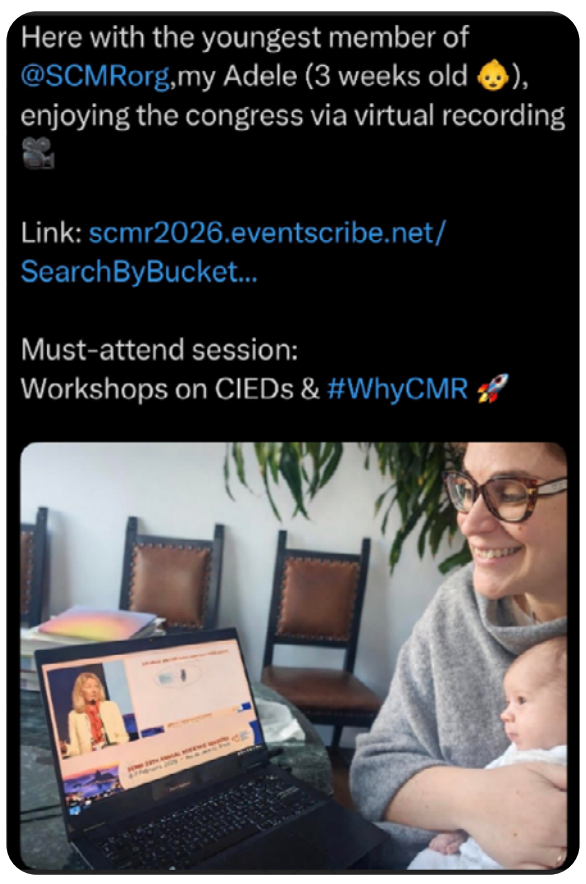

Anna Giulia Pavon, MD, FSCMR

2026 SCMR GRANTS

Science Seed Grant

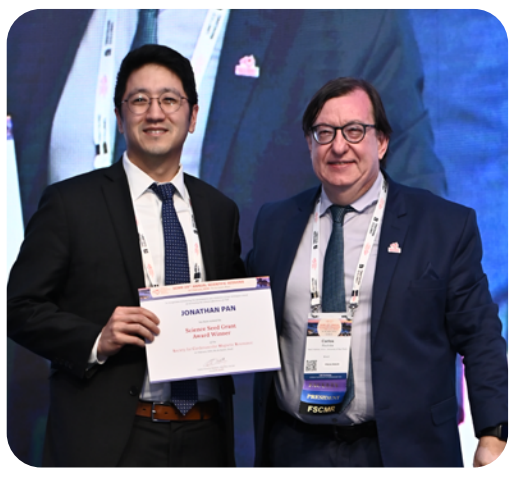

Jonathan Pan, MD, MBA, MS

Assistant Professor of Medicine, University of Virginia

Project: Validate novel cardiac magnetic resonance (CMR) sequences for exercise testing to better understand the mechanisms of exercise intolerance and improve the diagnosis of heart failure with preserved ejection fraction.

In recognition of fostering the development and validation of new techniques aimed at increasing the clinical effectivess of CMR

Outcomes Research Grant

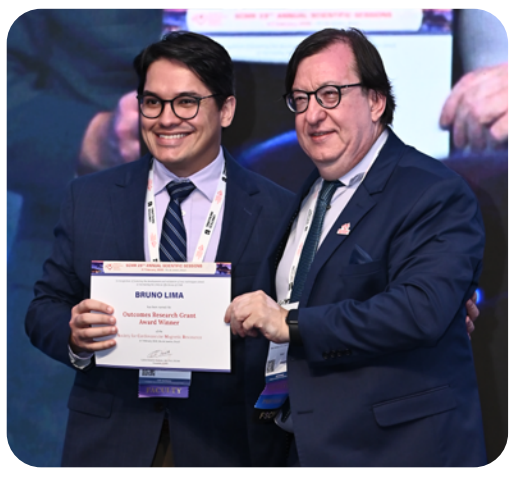

Bruno Lima, MD, PhD

Assistant Professor of Medicine, Vanderbilt University Medical Center

Project: VALID-TR: Validation of Advanced Liver and Imaging Diagnostics in Tricuspid Regurgitation via the SCMR Registry.

In recognition of fostering the development and validation of new techniques aimed at increasing the clinical effectivess of CMR

Training Fellowship Grant

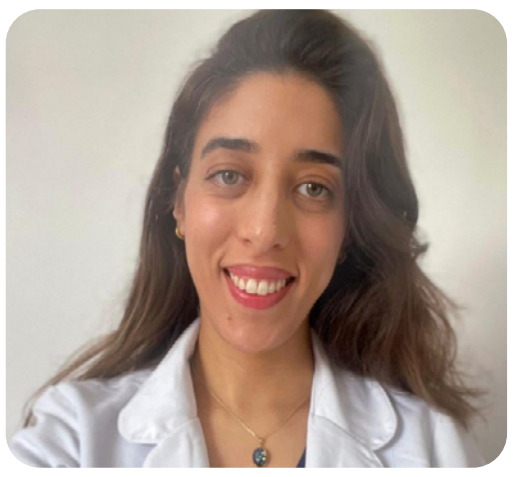

Fatima-Zahrae Chrifi, MD

Cardiology resident, Ibn Sina University Hospital Center, Rabat, Morocco

In recognition of fostering advanced training in cardiovascular magnetic resonance imaging to expand equitable global access to CMR expertise.

Training center: University of Virginia (UVA), Virginia, USA
Duration: 12 months

Pediatric/Congenital Heart Disease Early/Mid-Career Visiting Fellowship Grants

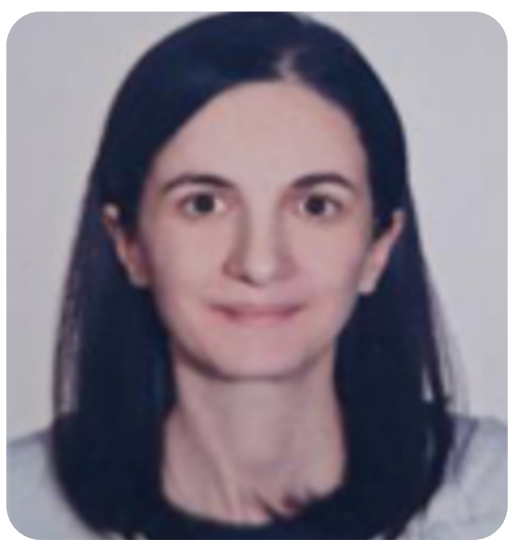

Dimah Aljarmakani, MD

Children's University Hospital,Damascus, Syria

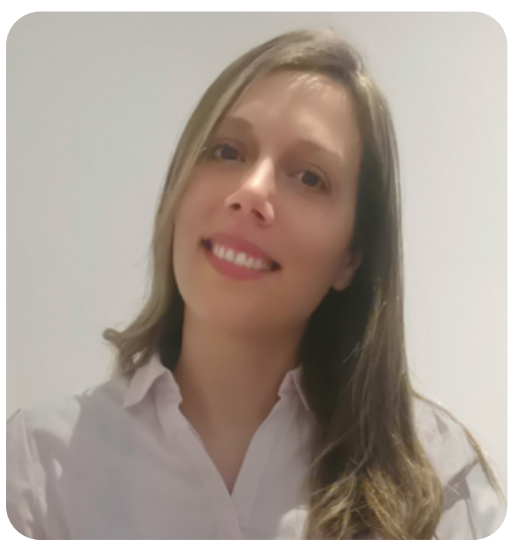

Julieta Anahi Rancati, MD

Hospital de Niños Pedro Elizalde, Buenos Aires, Argentina

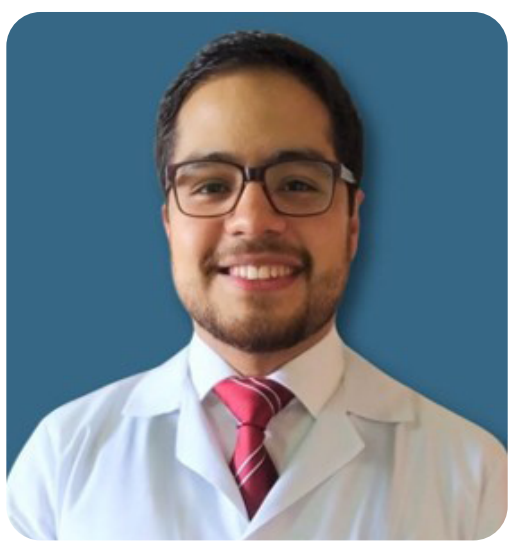

Jorge Silva Estrada, MD

Instituto Nacional de Pediatría, Mexico City

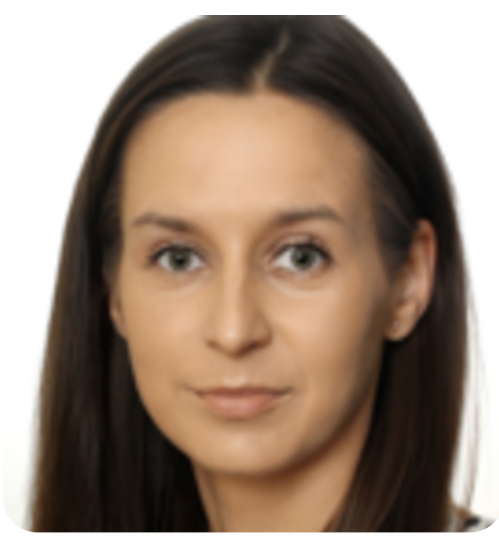

Monika Rozewicz Juraszek, MD

City Hospital, Gliwice, Poland

This grant is designed to support early- and mid-career members in accessing unique clinical or research opportunities abroad.

SCMR 2026

COMMUNITY BUILDING & NETWORKING

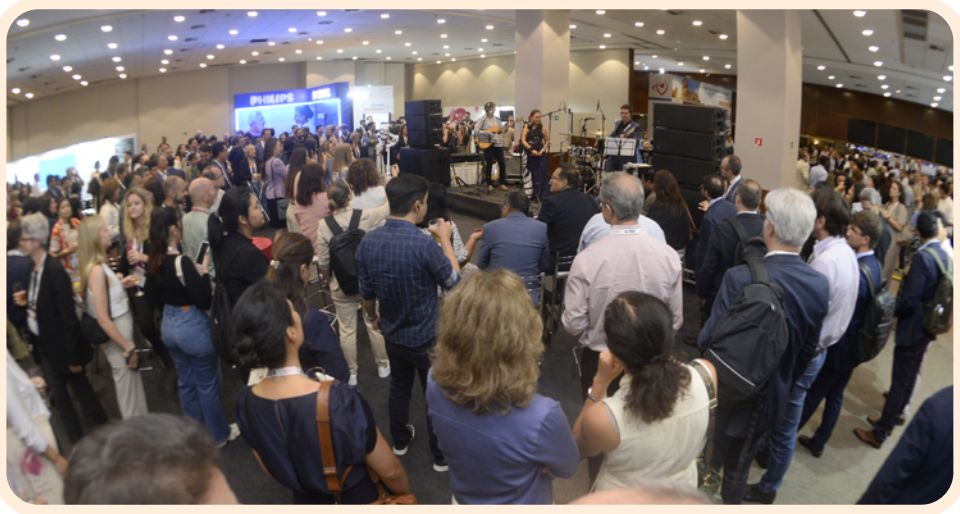
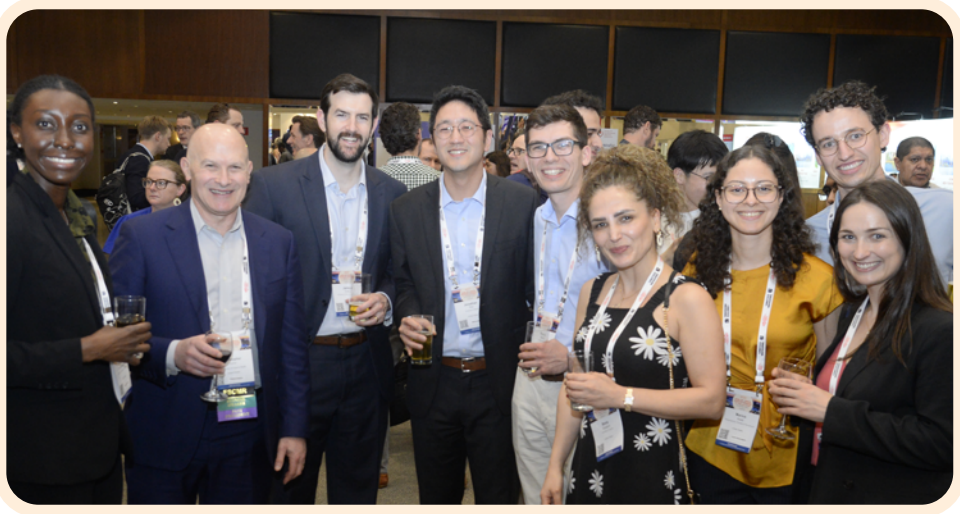
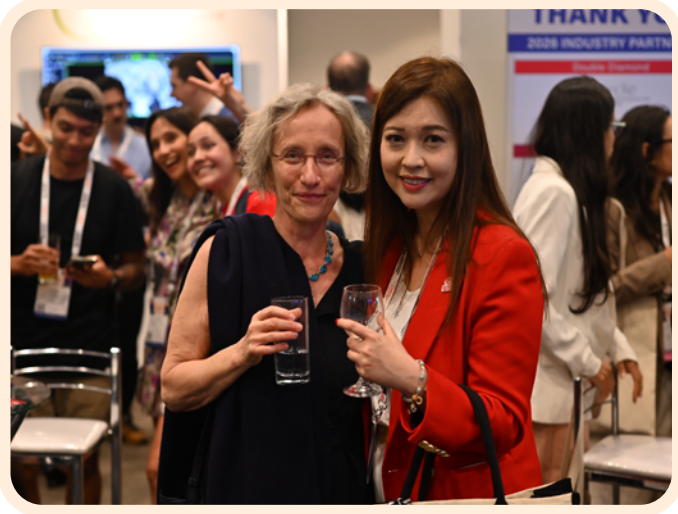
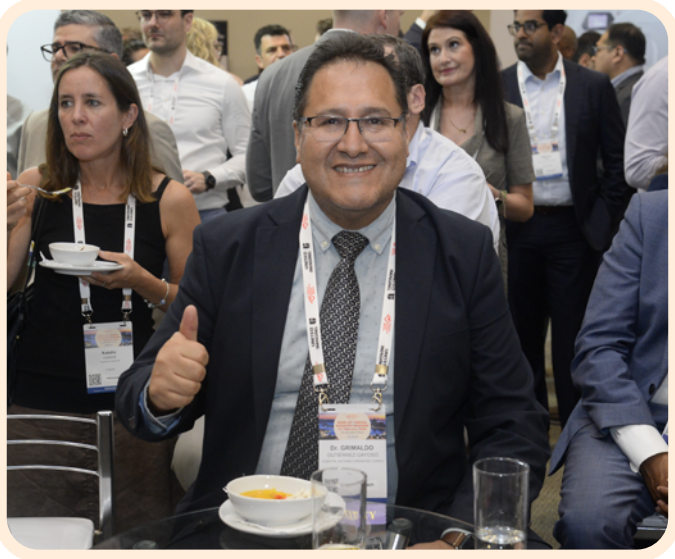
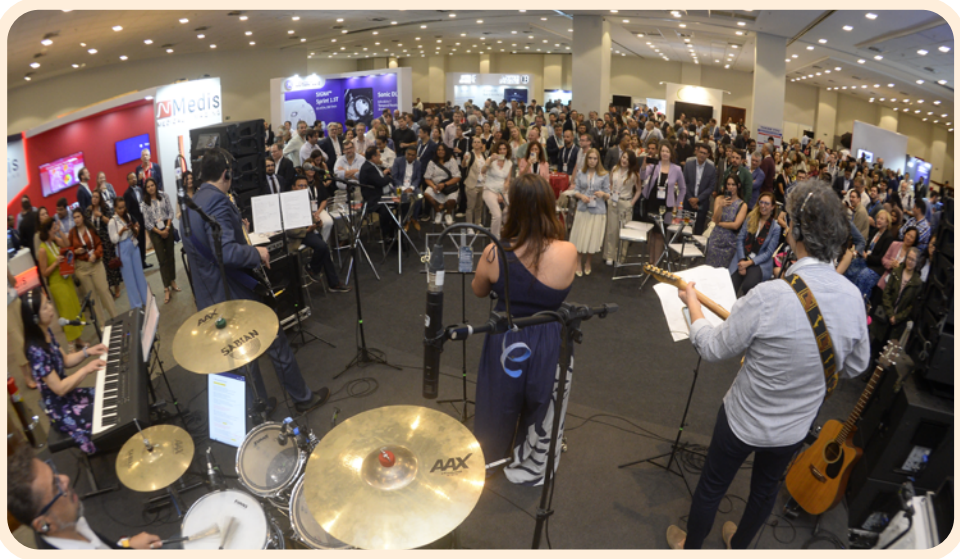
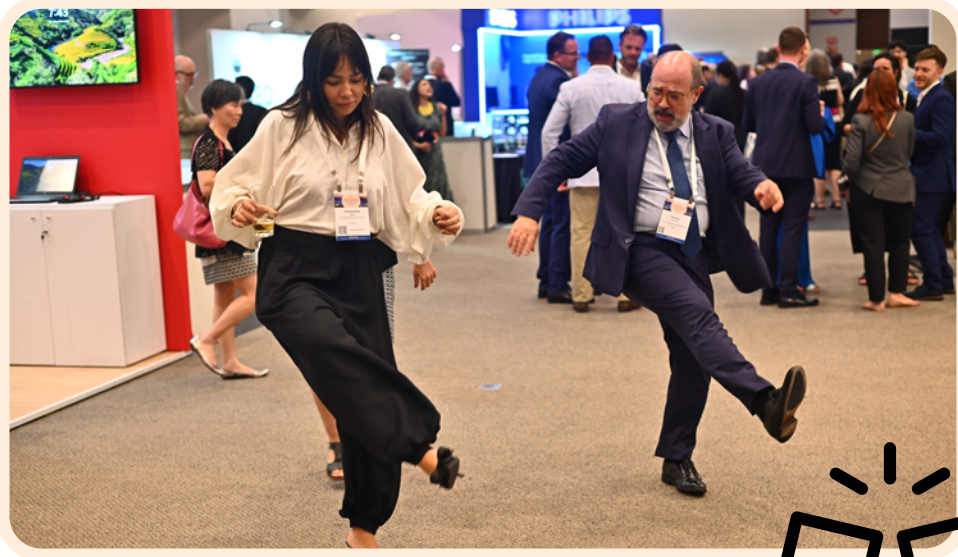
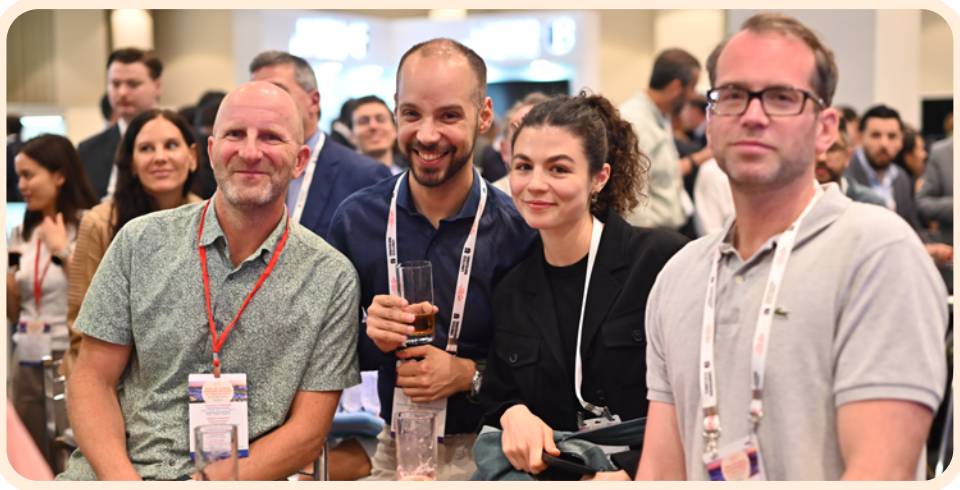
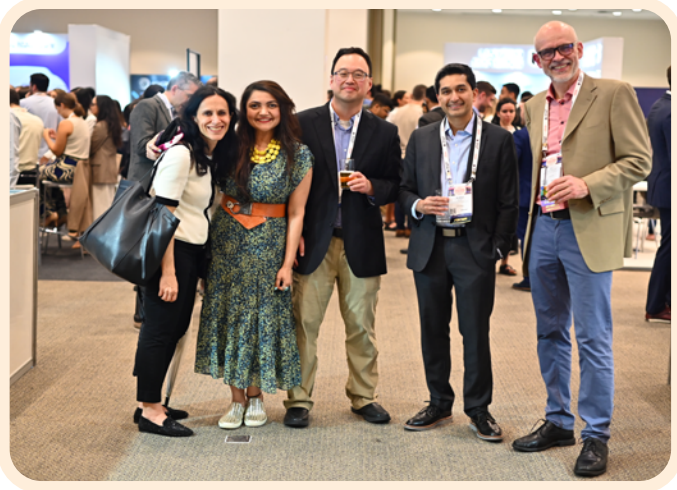

Welcome Reception

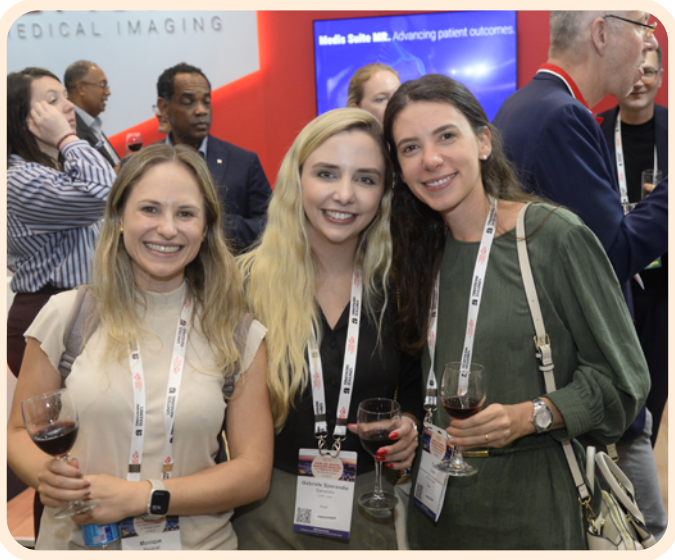
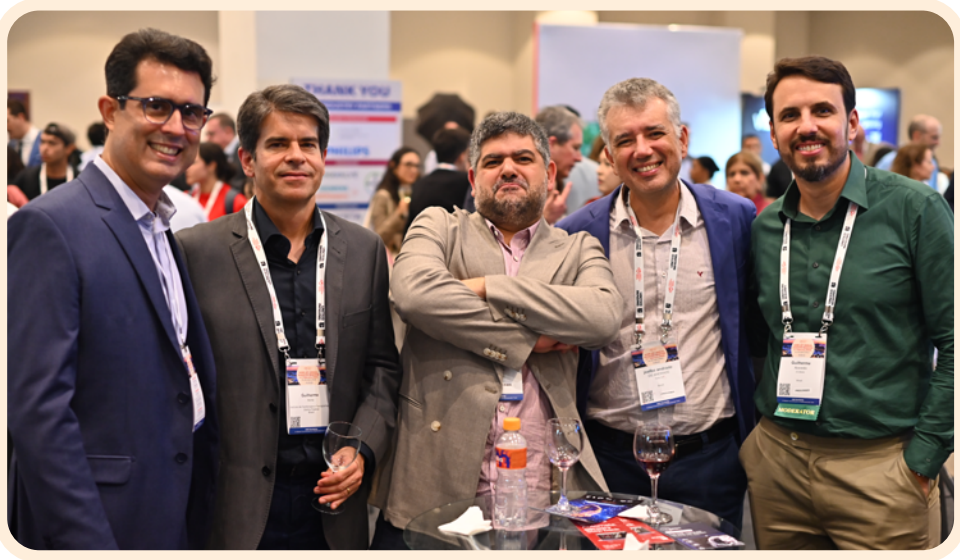
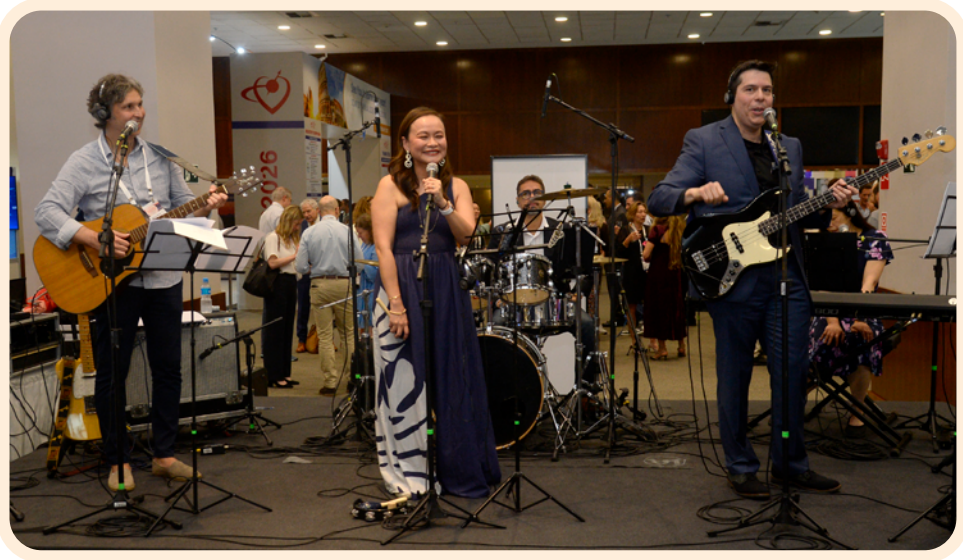

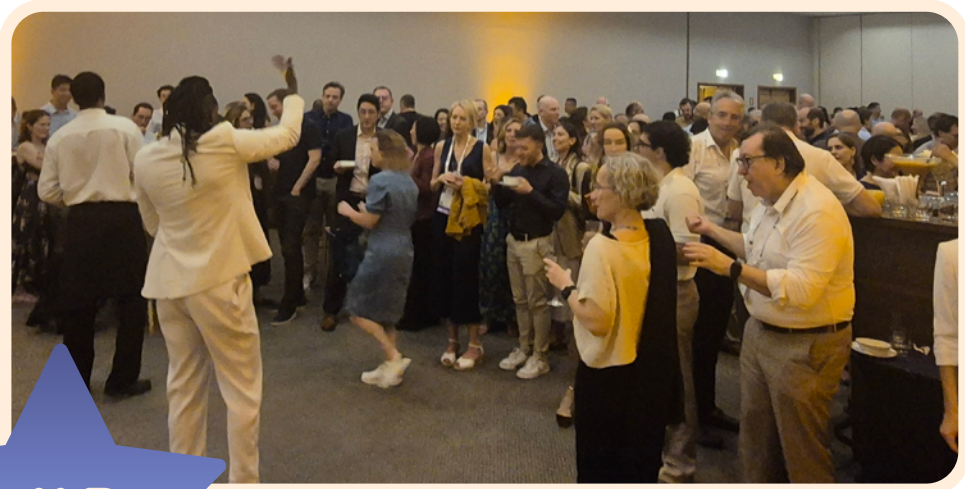
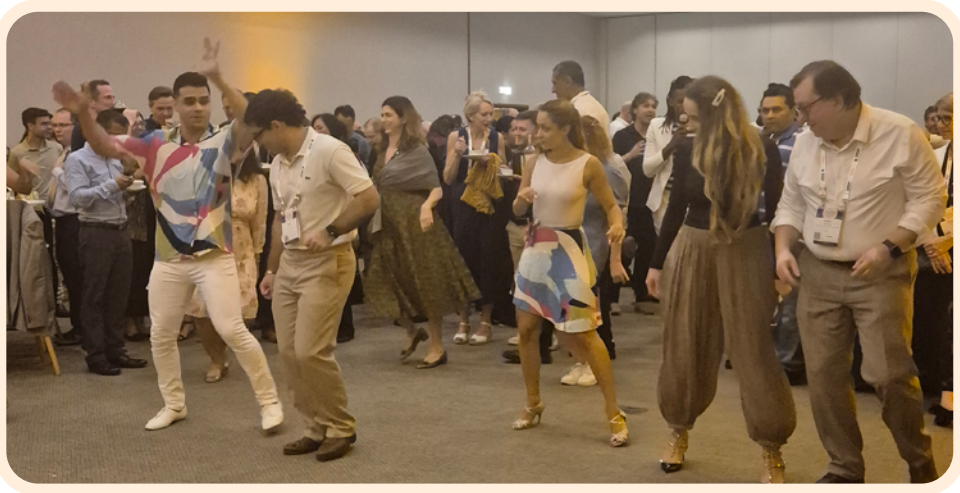
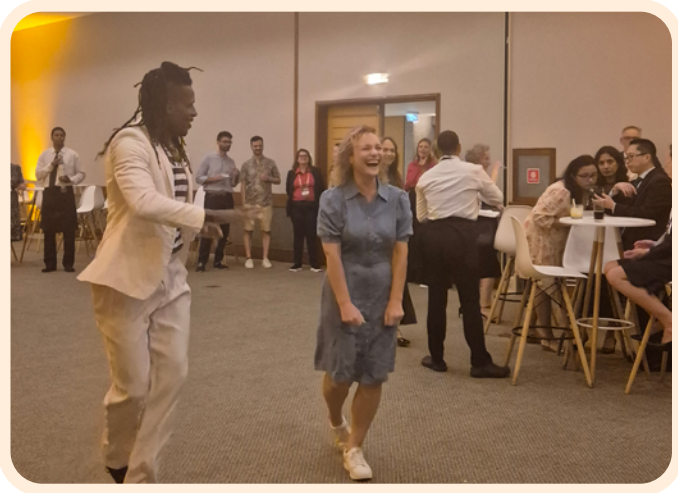

VIP

Faculty Reception and samba show!

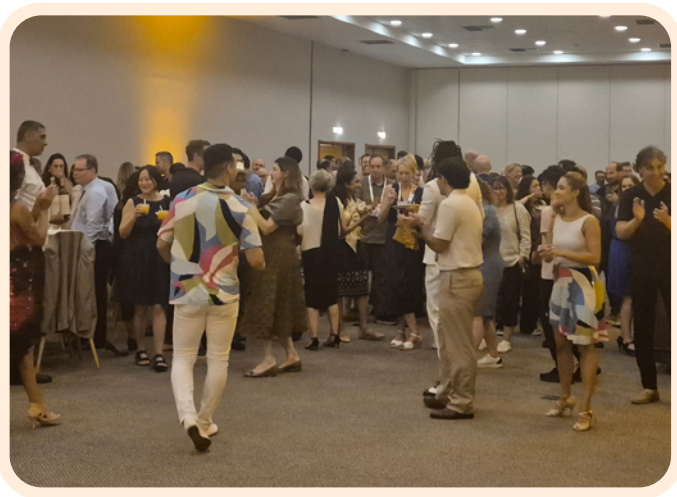
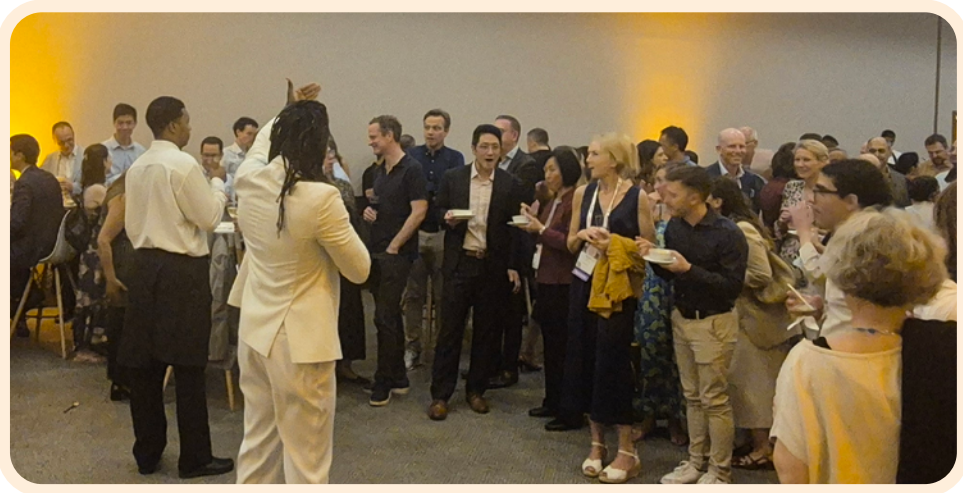

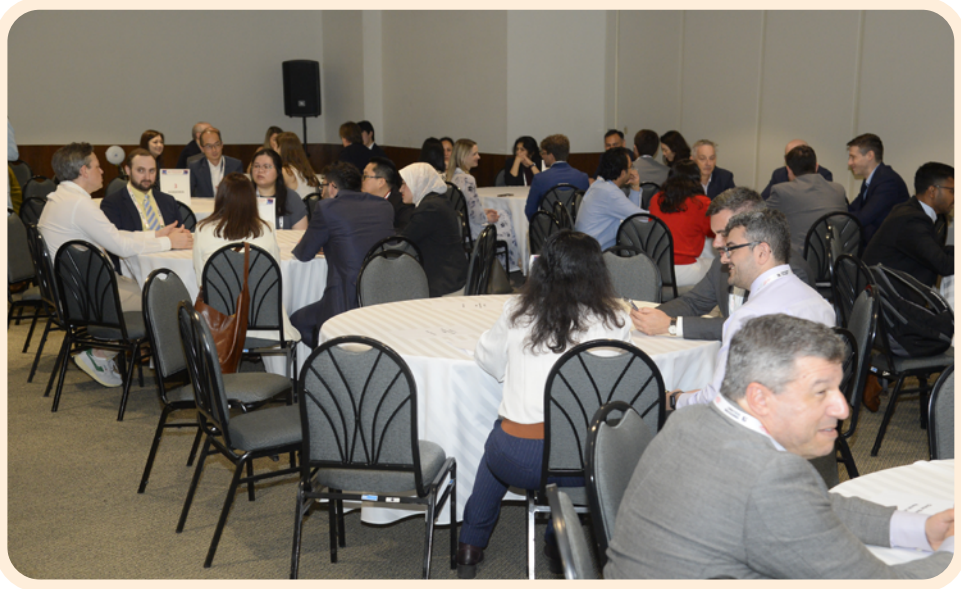
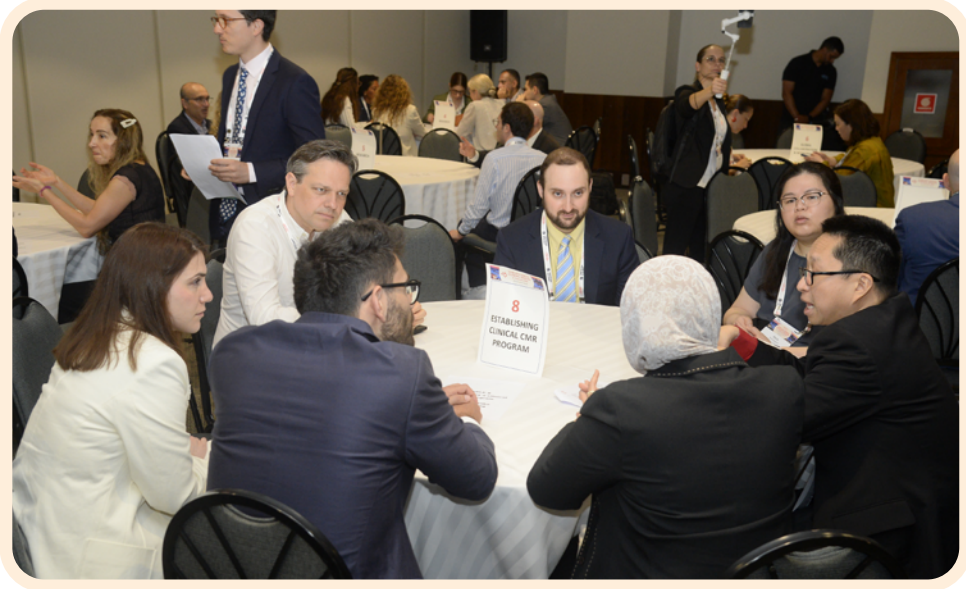
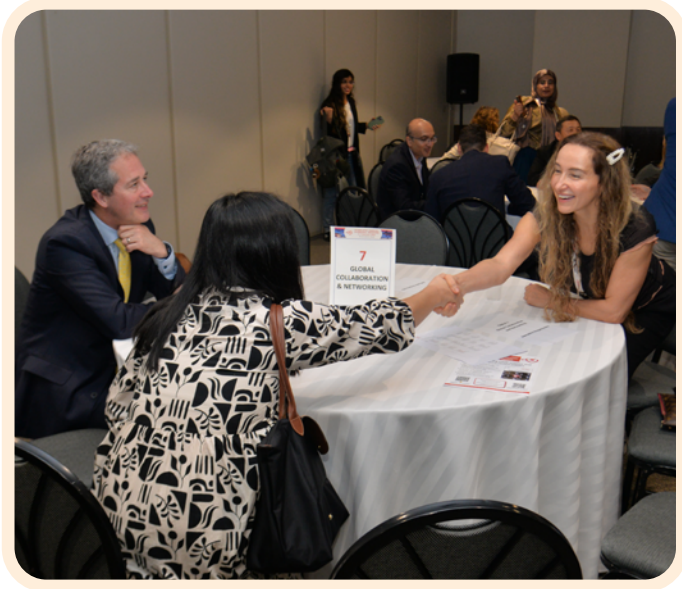

Speed Mentoring

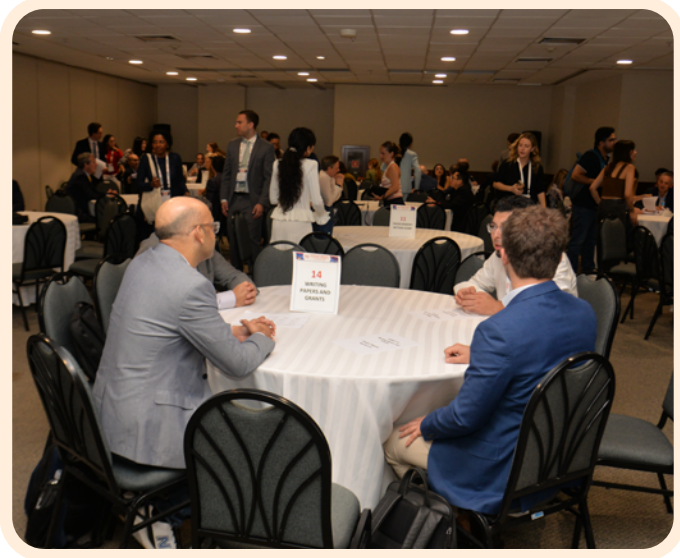
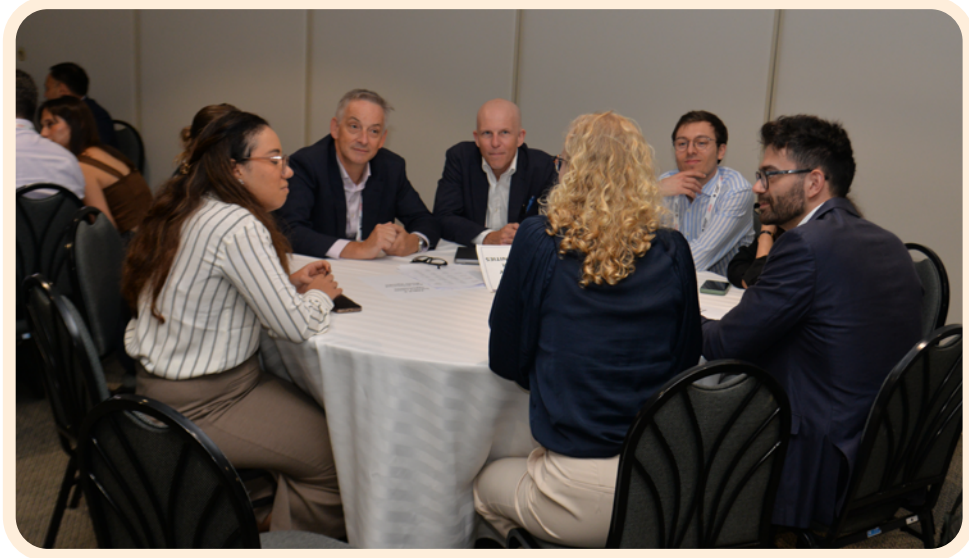
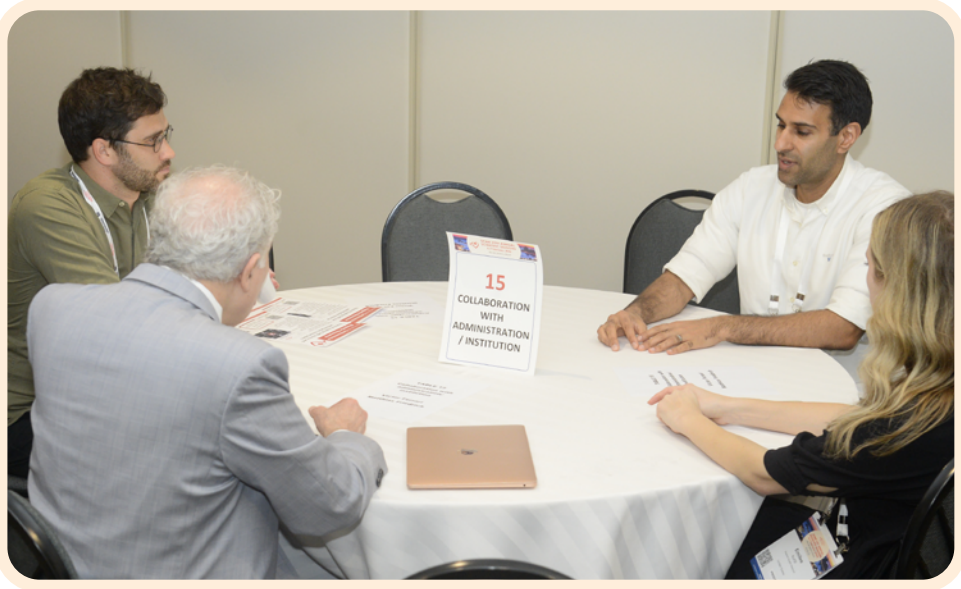
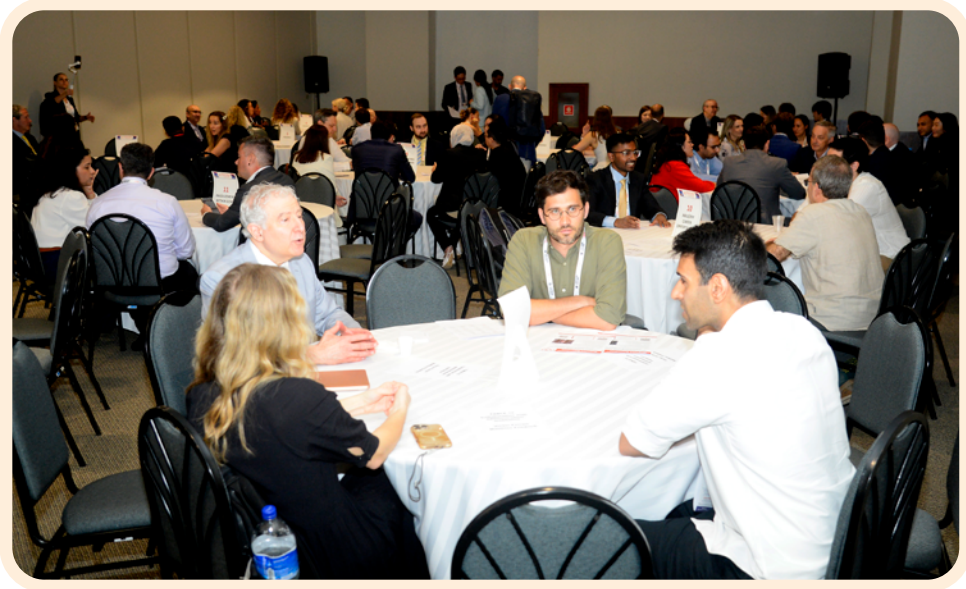
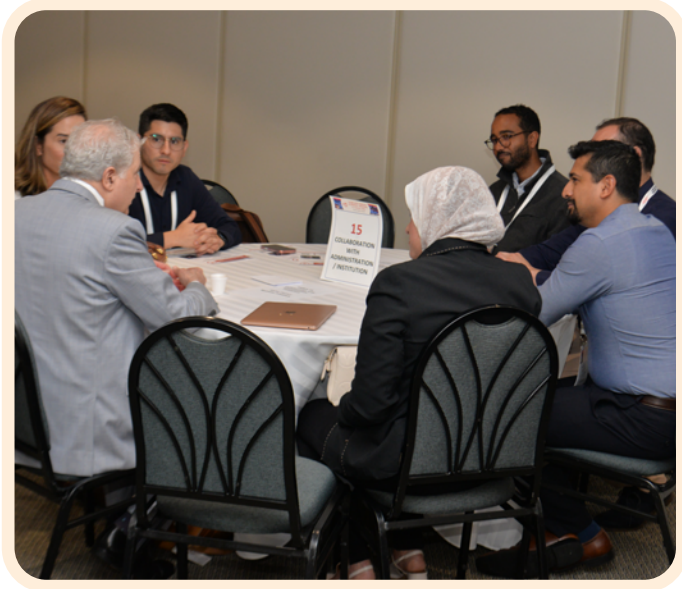

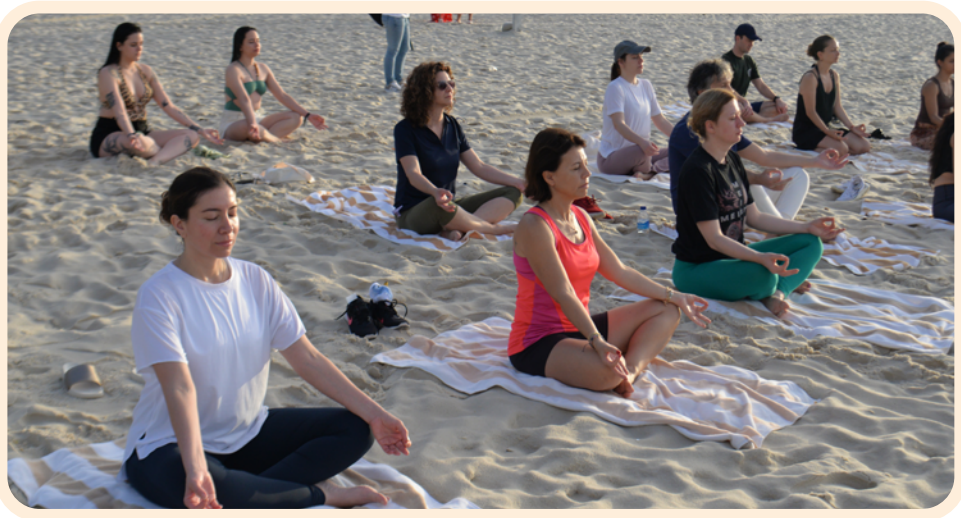
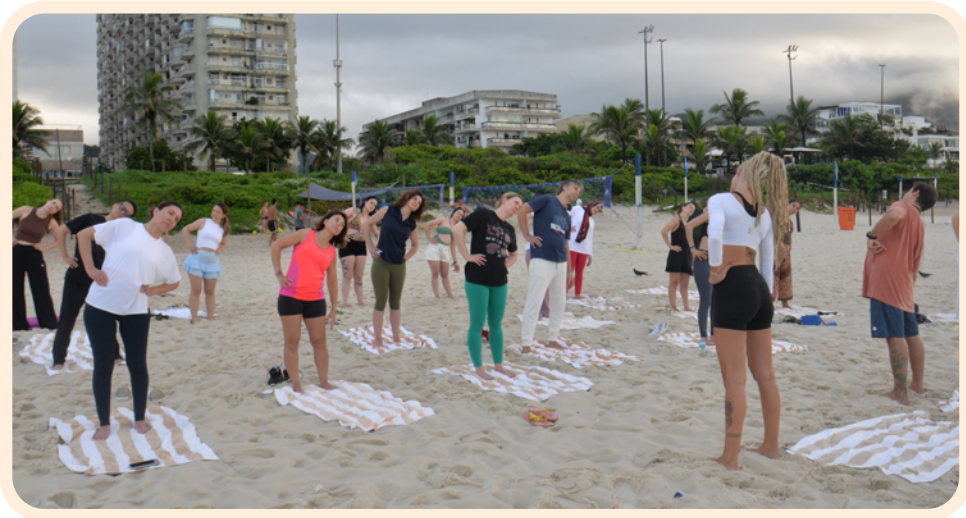
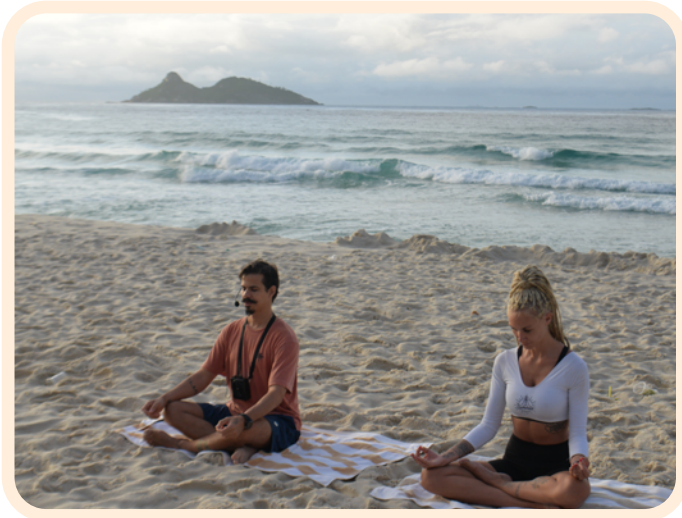
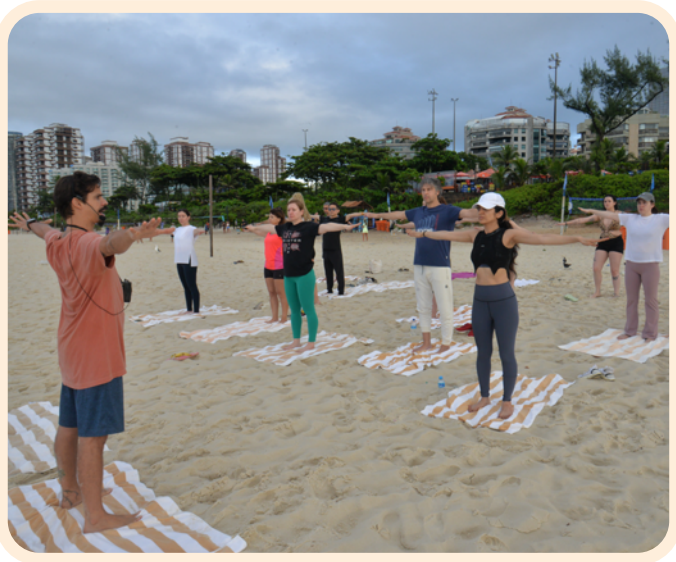
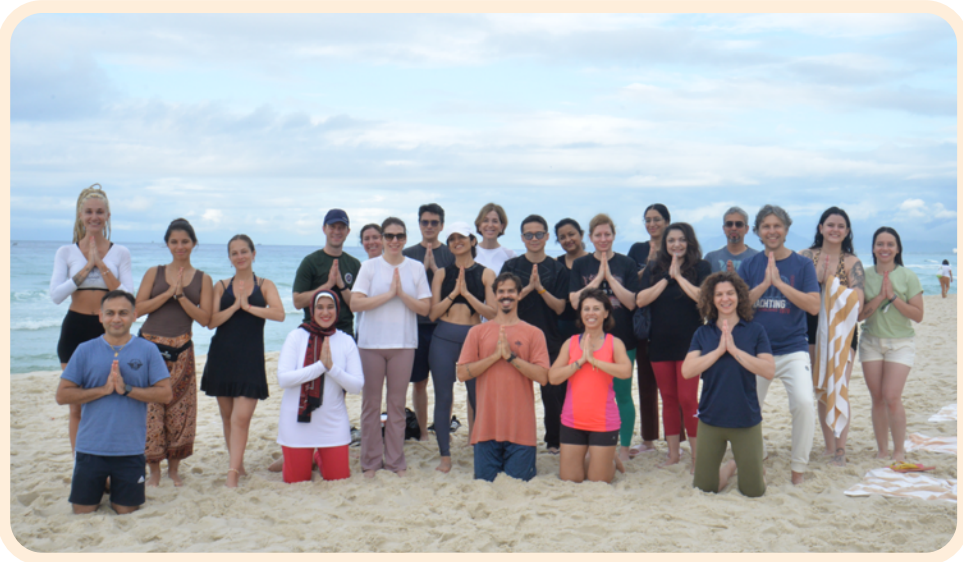

Science by the Shore

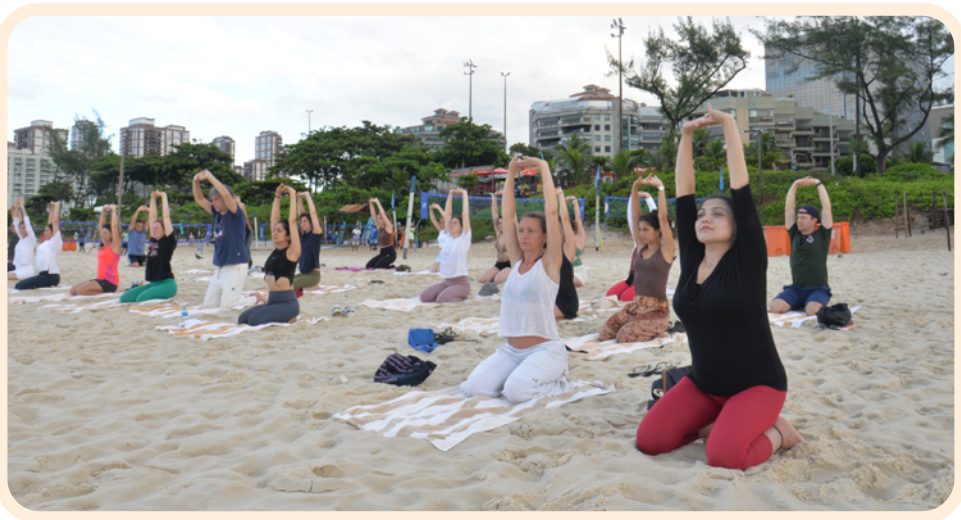
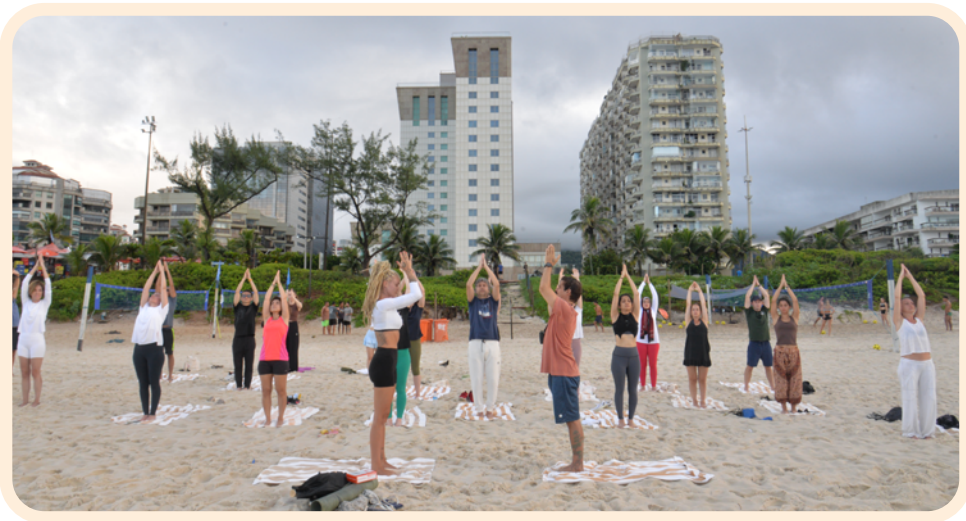
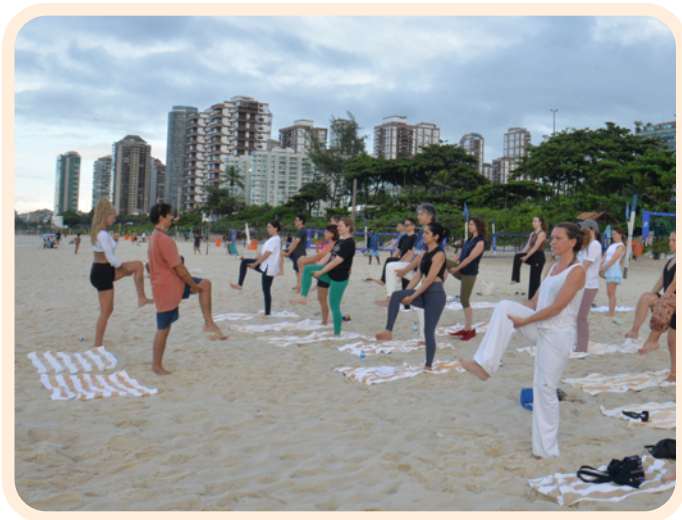

MARKETING & COMMUNICATION
ONLINE

E-mailing Campaign

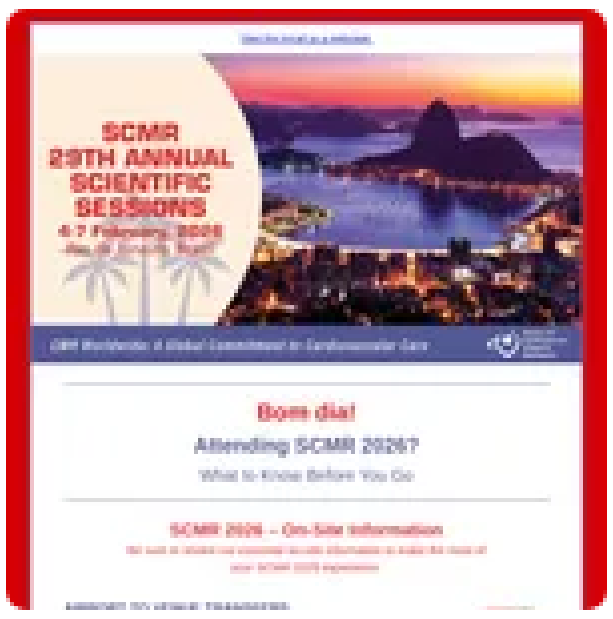

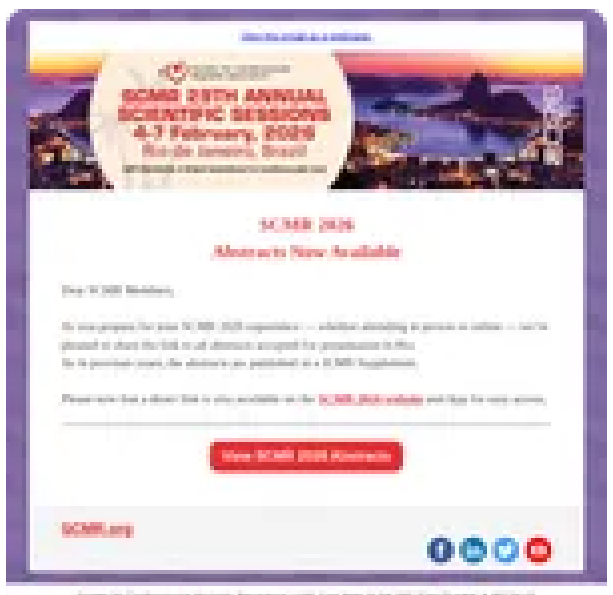

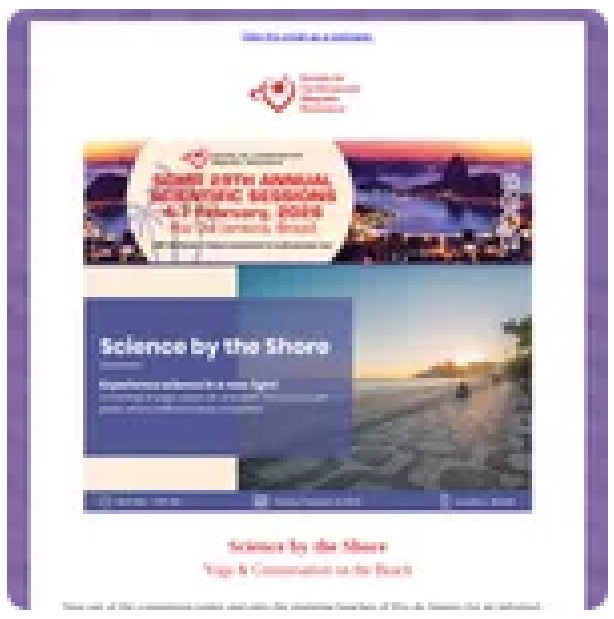

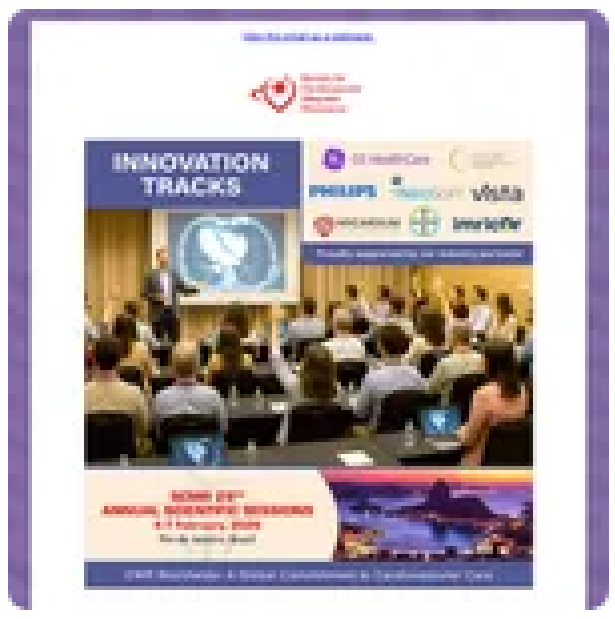

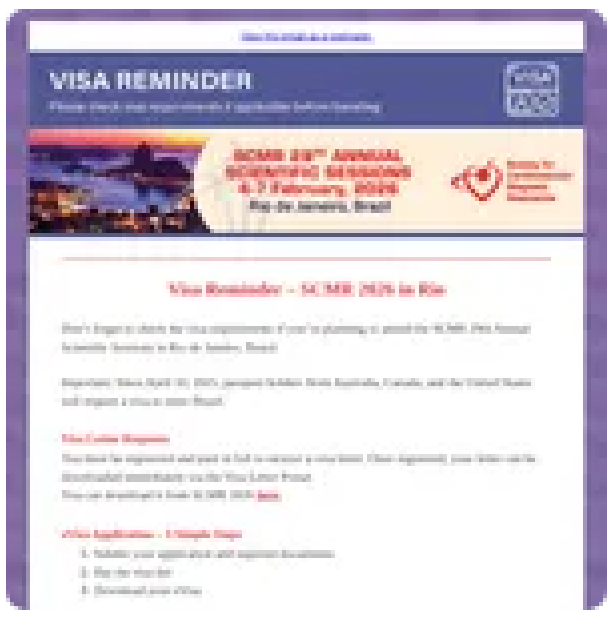

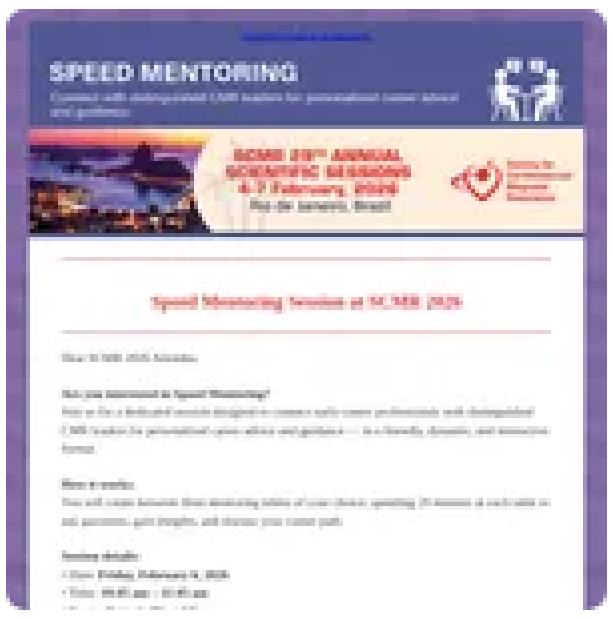

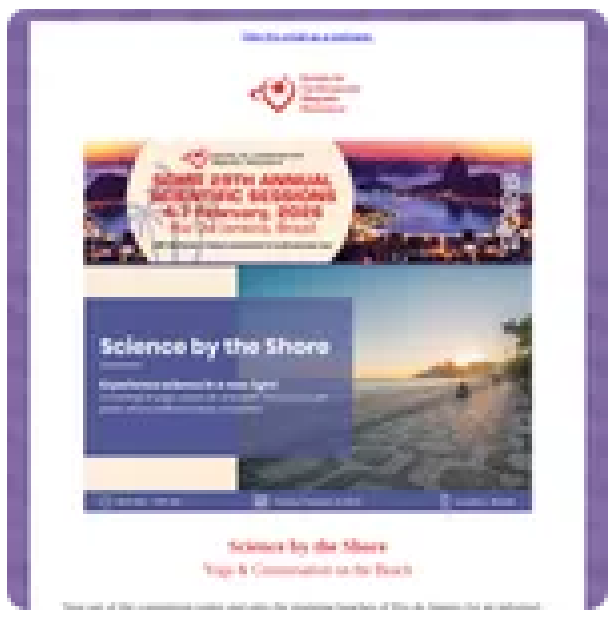

36
E-blasts Sent

Social Media

192 publications across all social media platforms

1 Video Teaser

6 Promotional Videos

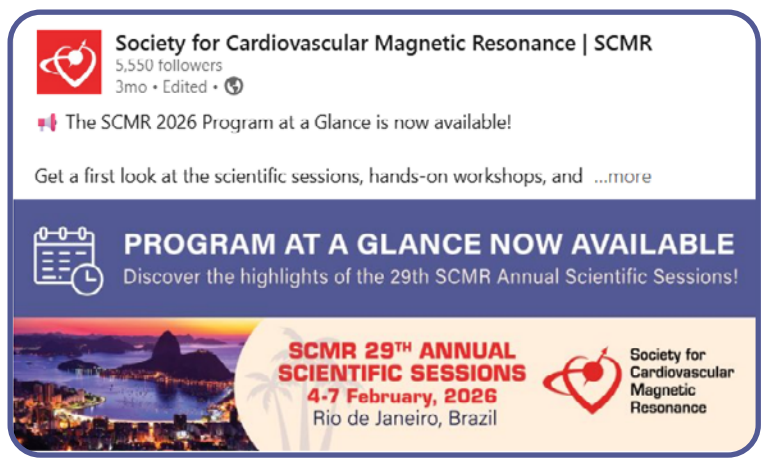

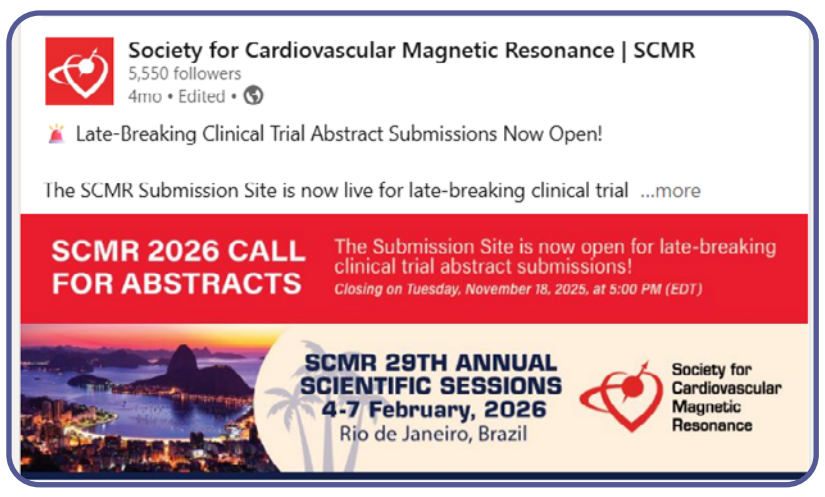

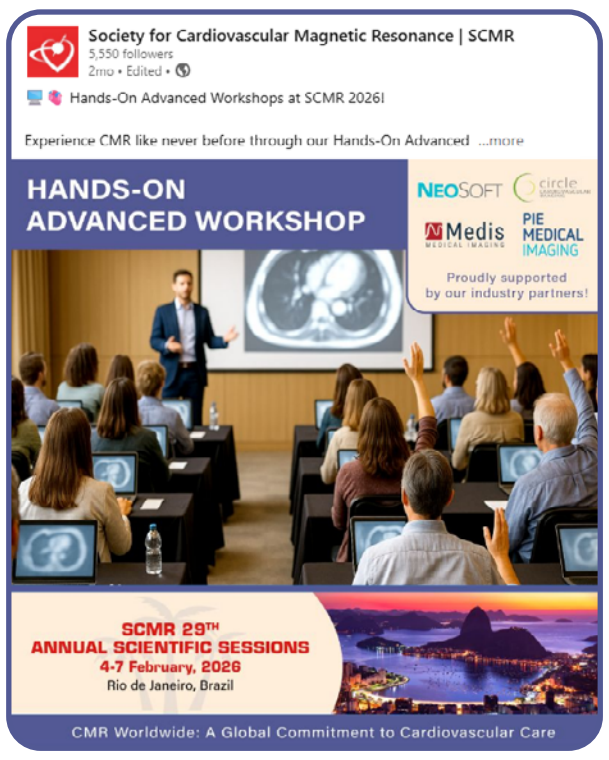

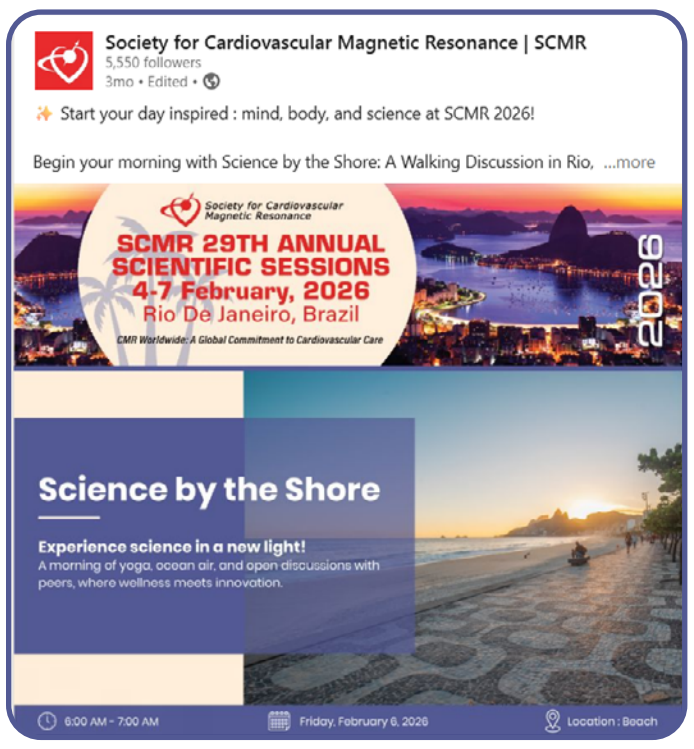

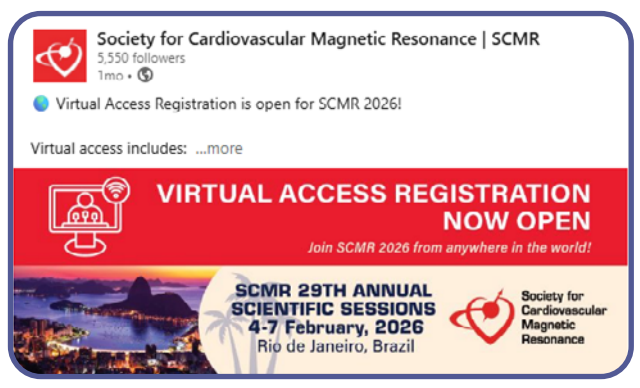

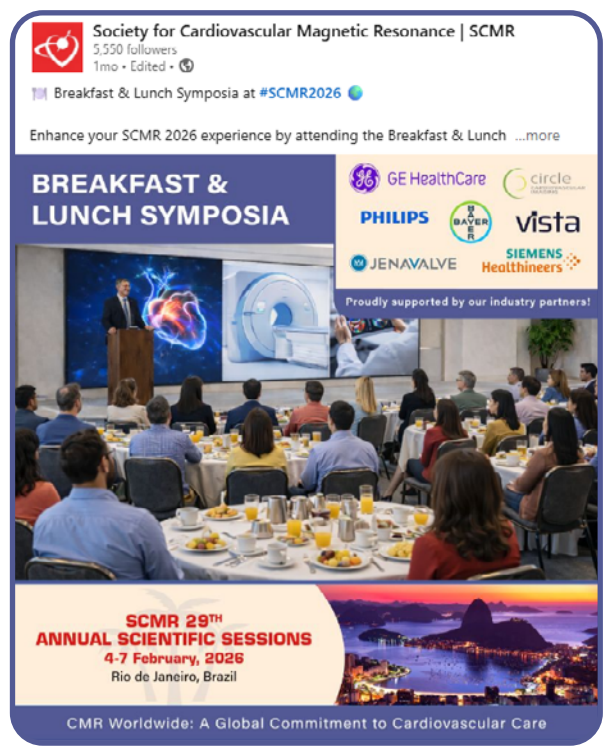

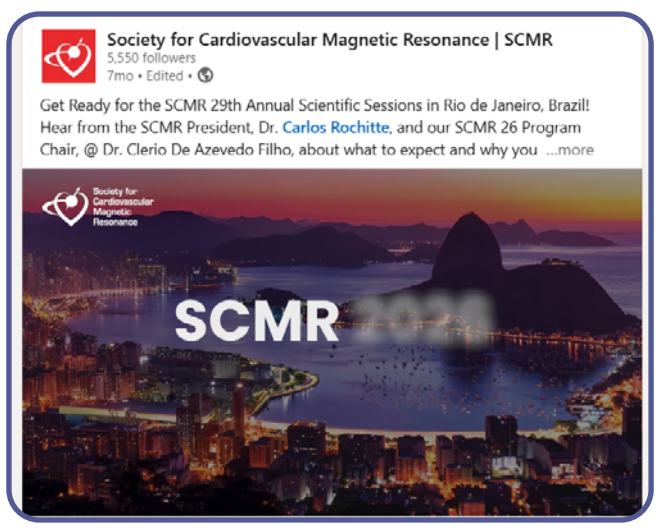

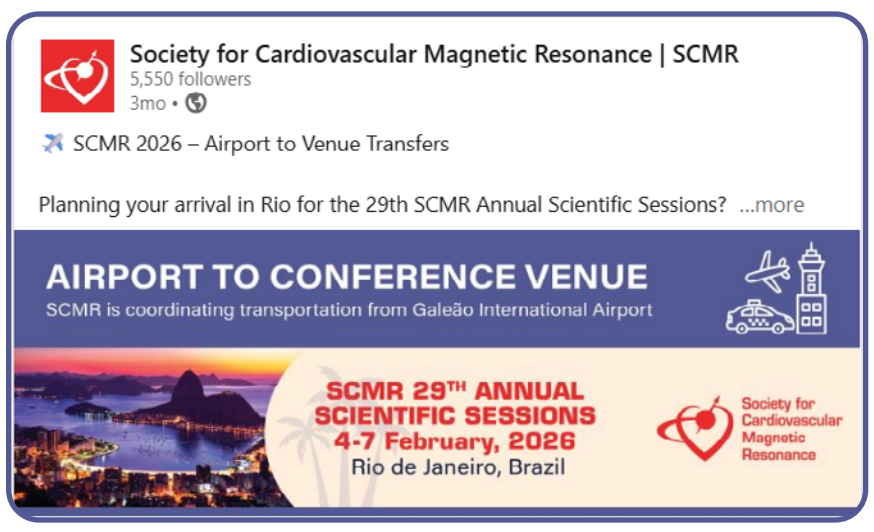

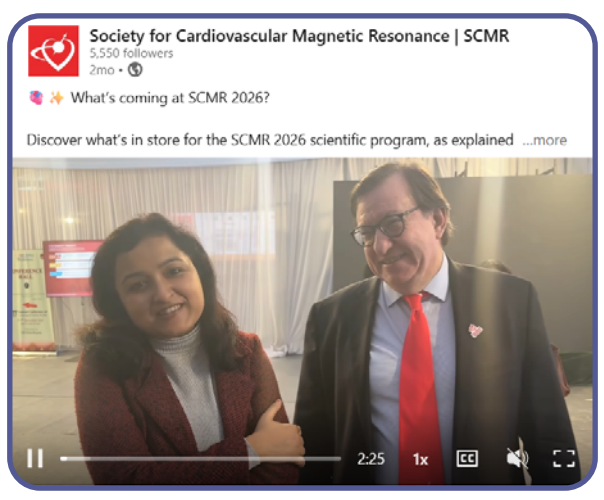

MARKETING & COMMUNICATION
ONLINE

SCMR 2026 Key Metrics Overview

LinkedIn

BEFORE SCMR 2026 (≈ 5 months)
Impressions: 142,223 **(+185.2%)**
Reactions: 2,158 **(+216.9%)**
Comments: 70 **(+677.8%)**
Reposts: 35 **(+84.2%)**
New Followers: 899 **(+132.9%)**

DURING + POST EVENT (23 days)
Impressions: 35,898 **(+49%)**
Reactions: 880 **(+118.9%)**
Comments: 13 **(+160%)**
Reposts: 10 **(-16.7%)**
New Followers: 221 **(+45.4%)**

The pre-congress period covers nearly five months, while the congress and post-event phase represents less than one month.
Yet in only 23 days, LinkedIn generated:
221 new followers (≈ 25% of the 5-month total)
880 reactions (≈ 40% of the 5-month total)
Nearly 36,000 impressions
When adjusted for time, the Conference period demonstrates a clear acceleration effect in both visibility and audience growth.

Although the pre-Conference period spans nearly five months, the 23-day Annual Meeting + post-event phase clearly demonstrates **the strengthening effect of SCMR 2026 communication on engagement growth.**
The Annual Scientific Sessions acted as a powerful driver, reinforcing visibility, interaction, and follower momentum on LinkedIn.

Facebook

BEFORE SCMR 2026 (≈ 5 months)
Views: 54,879 **(+258%)**
Interactions: 822 **(+627.4%)**
Reactions: 709
Comments: 38
Shares: 75
Total Followers: 3,746 **(+2.0%)**
Net Follows: 73

DURING + POST EVENT (23 days)
Views: 10,185 **(-1%)**
Interactions: 127 **(-42.3%)**
Reactions: 115
Comments: 8
Shares: 4
Total Followers: 3,746 **(+0.6%)**
Net Follows: 23

Although the pre-Conference period spans nearly five months, the 23-day Annual Meeting + post-event window maintained steady audience growth and engagement levels.
The communication surrounding SCMR 2026 contributed to sustaining visibility and follower acquisition momentum, reinforcing the Annual Meeting as a consistent digital visibility driver on Facebook.

X (formerly Twitter) – Analytics Limitation
Due to the use of a non-premium account, full analytics access is not available for X.

Bluesky doesn't currently provide built-in analytics on the platform.
No performance data is available.

Overall Digital Performance Conclusion – SCMR 2026

The digital communication strategy surrounding SCMR 2026 significantly strengthened SCMR online visibility and audience growth.
- On LinkedIn, the Annual Scientific Sessions acted as a clear **acceleration driver, generating concentrated engagement and follower growth** within a short 23-day window, despite the pre-Conference phase spanning nearly five months.
- On Facebook, the Conference period **helped sustain visibility and support continued community growth**, reinforcing its role within the Society's broader digital ecosystem.

Overall, SCMR 2026 functioned not only as a major scientific event for SCMR, but also as a strong driver of digital performance — enhancing engagement, expanding reach, and reinforcing SCMR's online presence.

MARKETING & COMMUNICATION
ONLINE

#SCMR2026 - Social Media Ambassadors

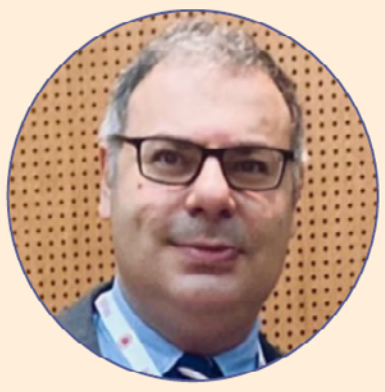
Vass Vassiliou
@vass_vassiliou

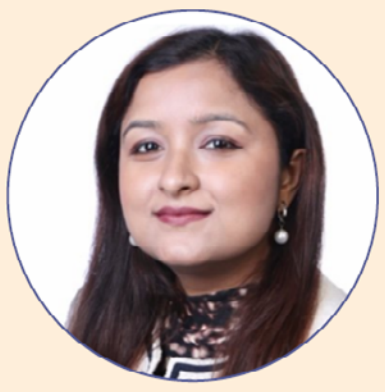
Vineeta Ojha
@vineetao17

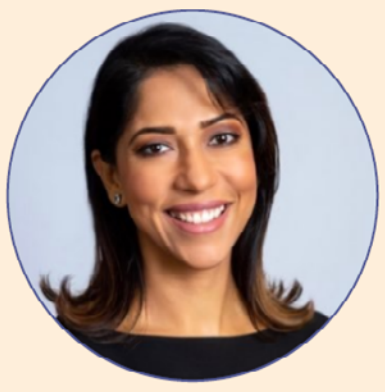
Kanwal Farooqui
@Kfarooqi

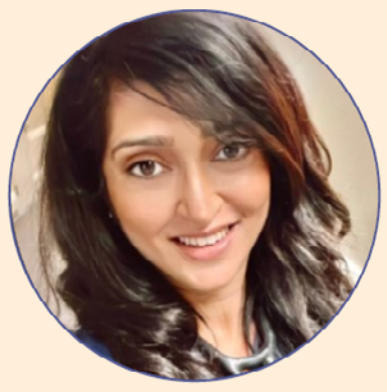
Purvi Parwani
@purviparwani

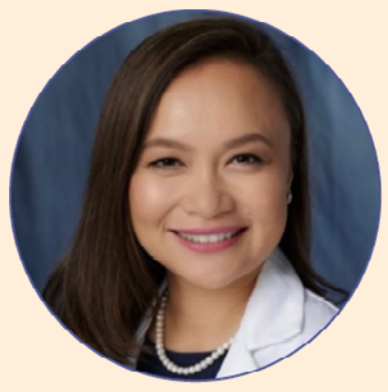
Jennifer Co Vu
@DrJenniferCo_Vu

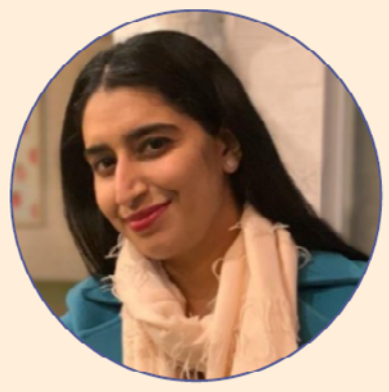
Mansi Verma
@MansiV1991

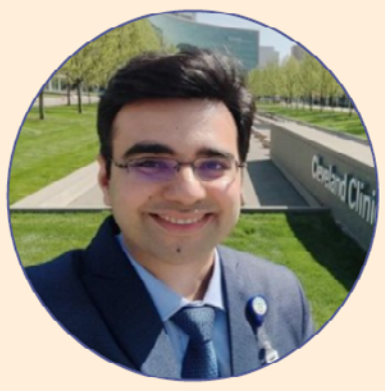
Rishabh Khurana
@rishk88

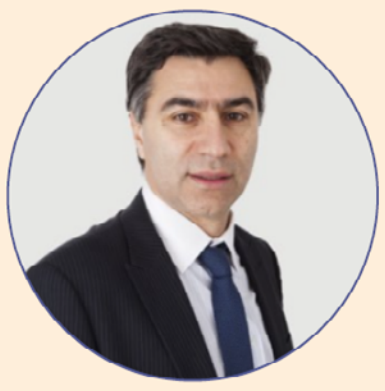
Yousef Daryani
@ydaryani

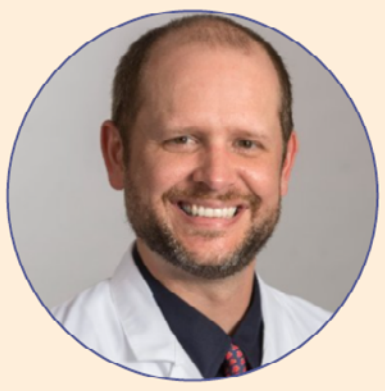
Jason johnson
@Jason_N_Johnson

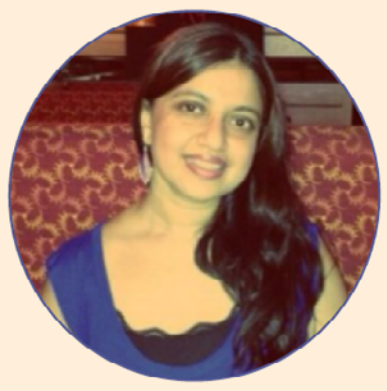
Nita Ray Chaudhary
@RayRcnita

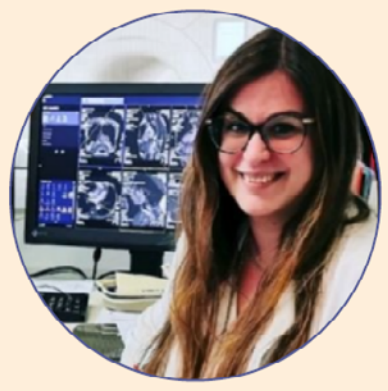
Anna Giulia Pavon
@annagiuliapavon

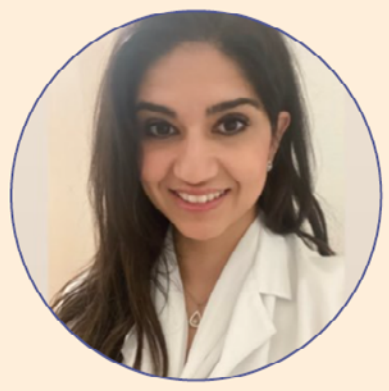
Sara Ersozlu
@ErsozluSara

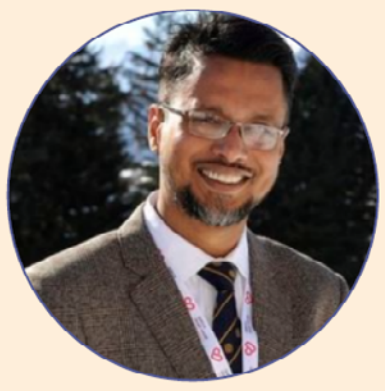
DMM Osmany
@DmmOsmany

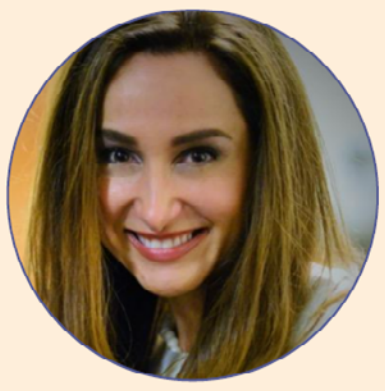
Jadranka Stovanovska
@JStojanovskaMD

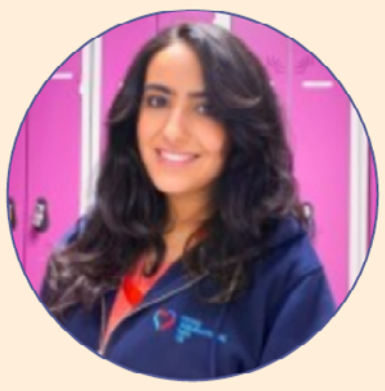
Soukaina Scadi
@soukaina scadi

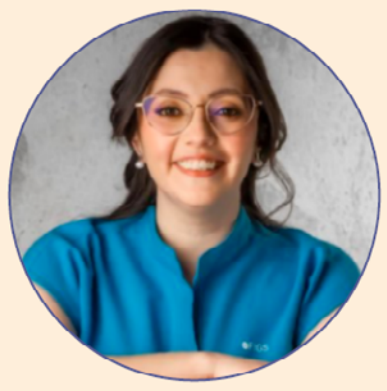
Laura Victoria
@VictoriaTAraujo

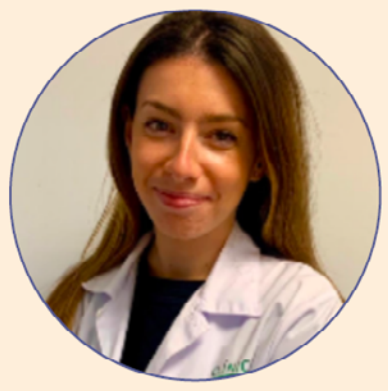
Blanca Domenech
@blanca_domenech

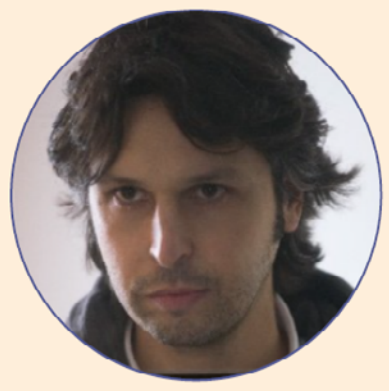
Alexandros Khallifatidis
@AKallifatidis

Conference - Trending

#SCMR2026
#ISC26
#ArabHealth2026
#HIMSS26
#EchoHawaii
#SCOPE2026
#Heartofcardio

Top 10 Influencial

1. @Jason_N_Johnson — Jason N. Johnson...
2. @purviparwani — Dr. Purvi Parwani
3. @DrJenniferCo_Vu — Jennifer Co-Vu, M...
4. @ydaryani — Yousef Daryani
5. @iamritu — Ritu Thamman MD
6. @AnnaGiuliaPavon — Anna Giulia Pavon
7. @LuchianLuiza — Maria Luiza Luchi...
8. @CarreroCeleste — Celeste Carrero
9. @SatoKeiMount9 — さとけい Keisuke ...
10. @TomoResoSac — @Tc-rmn-sac

Ranking calculated using three key influence metrics:
impressions (reach), reposts (amplification), and interactions (engagement)

MARKETING & COMMUNICATION
ON SITE

Main entrance

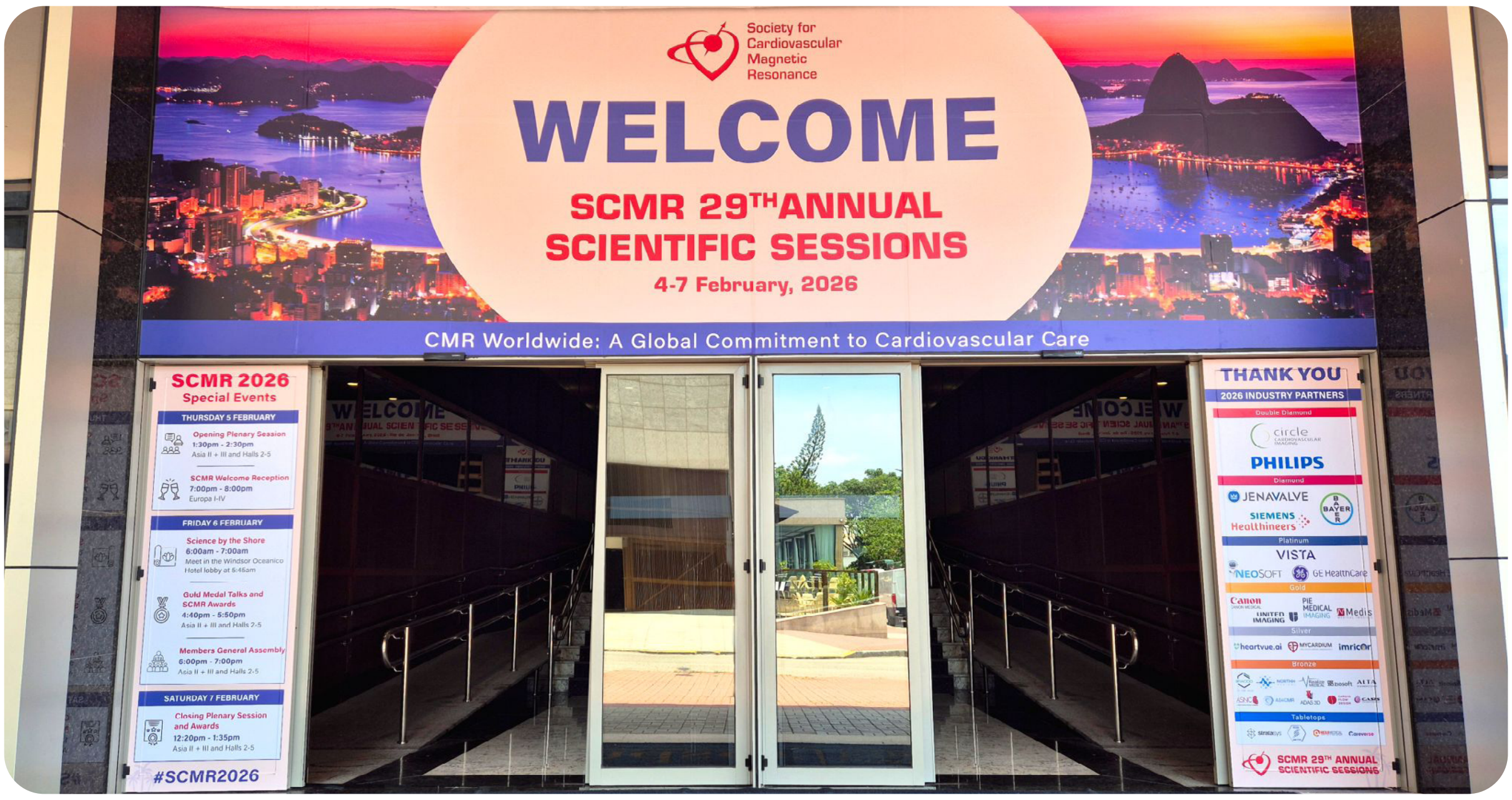

Exhibition hall entrance arch + stairs stickers

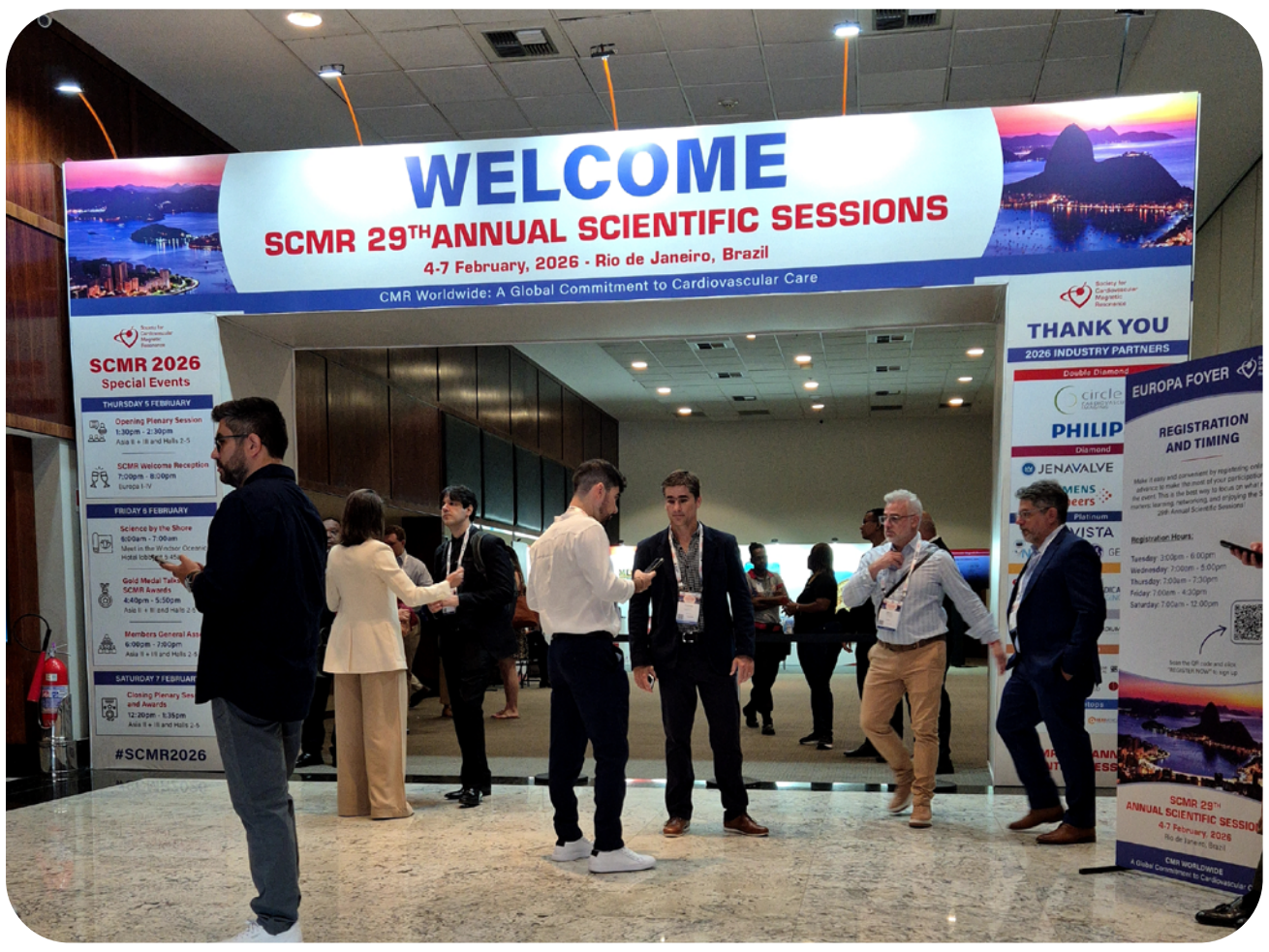

Front

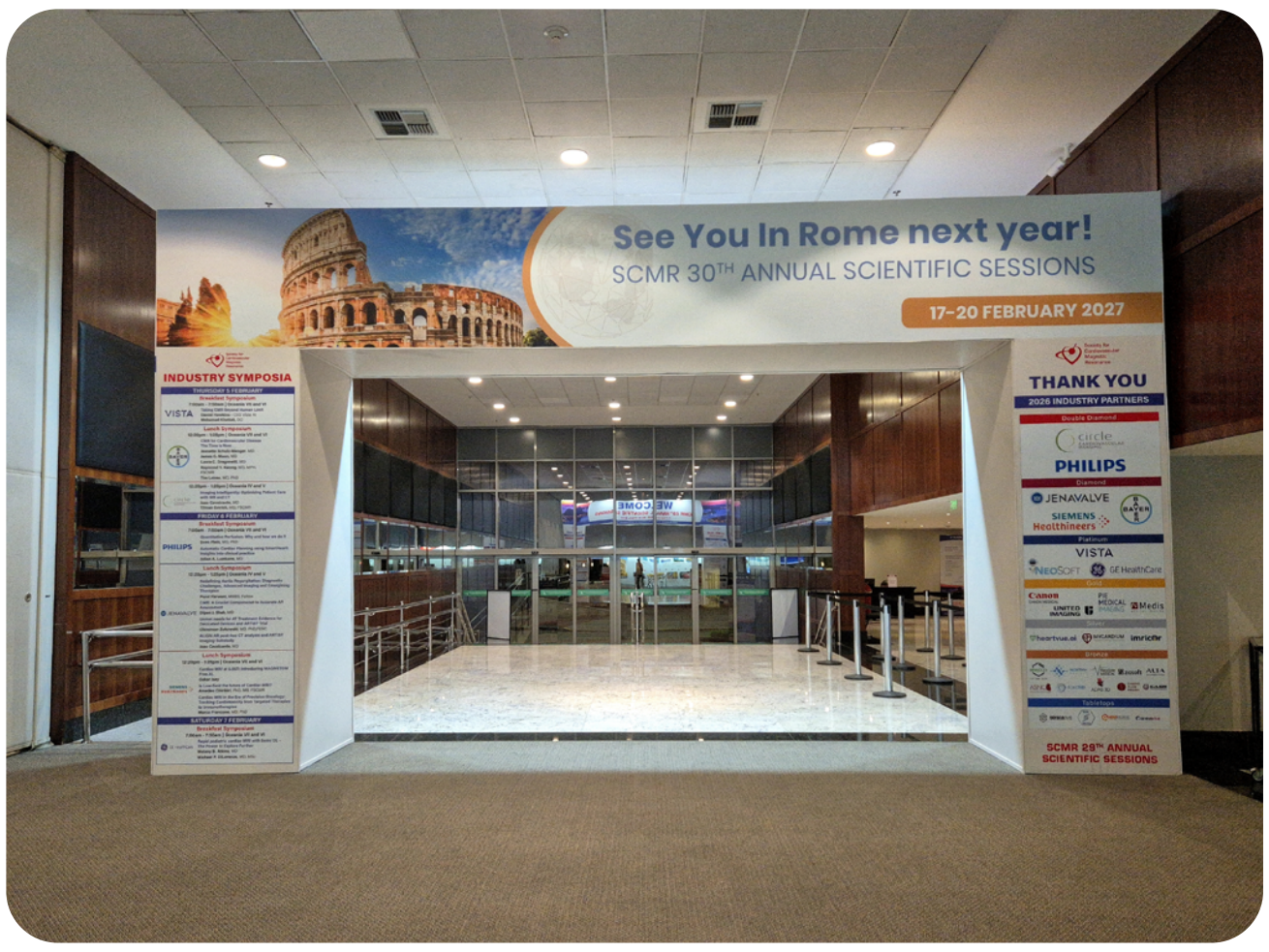

Back

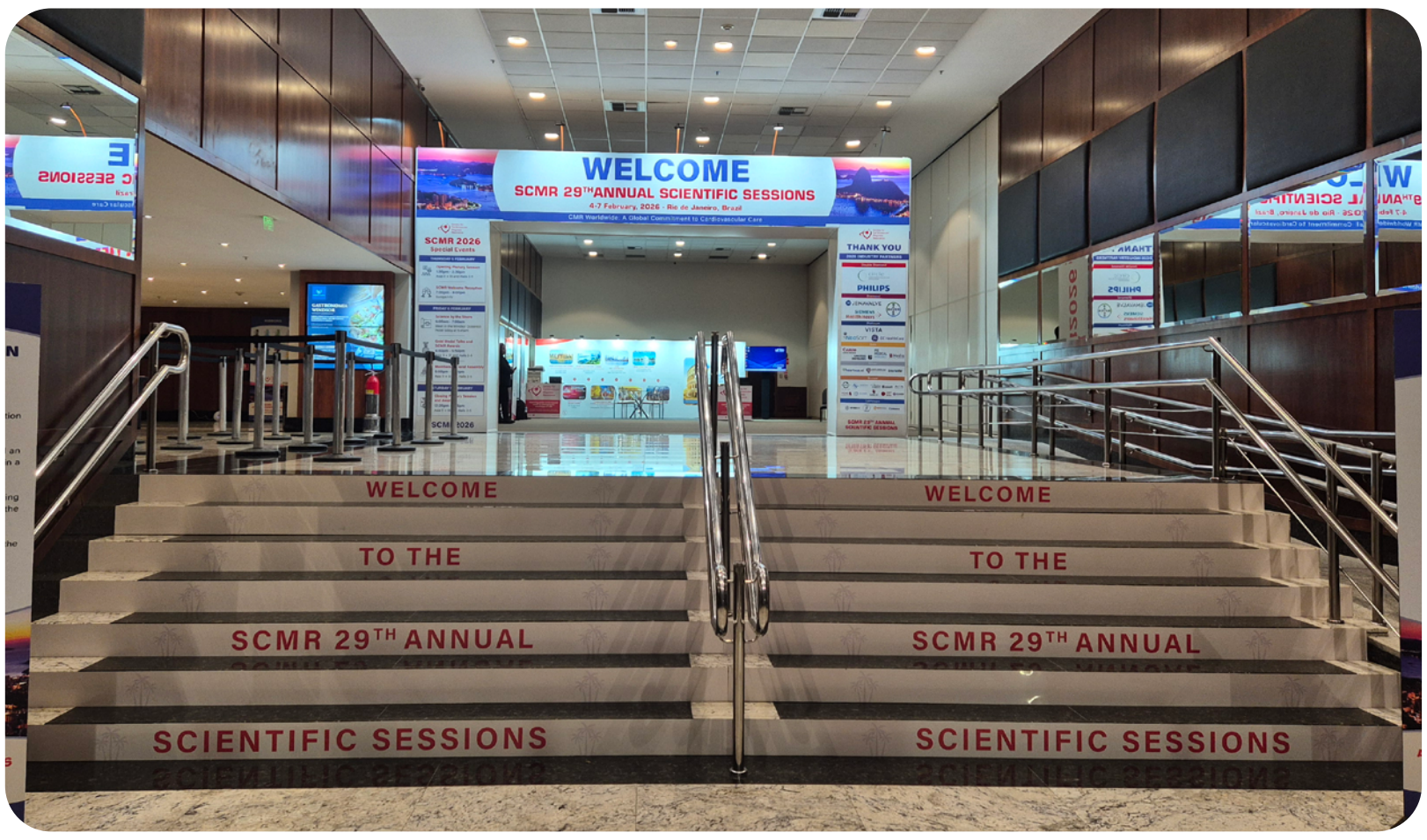

MARKETING & COMMUNICATION
ON SITE

Registration Desk & Event Timeline

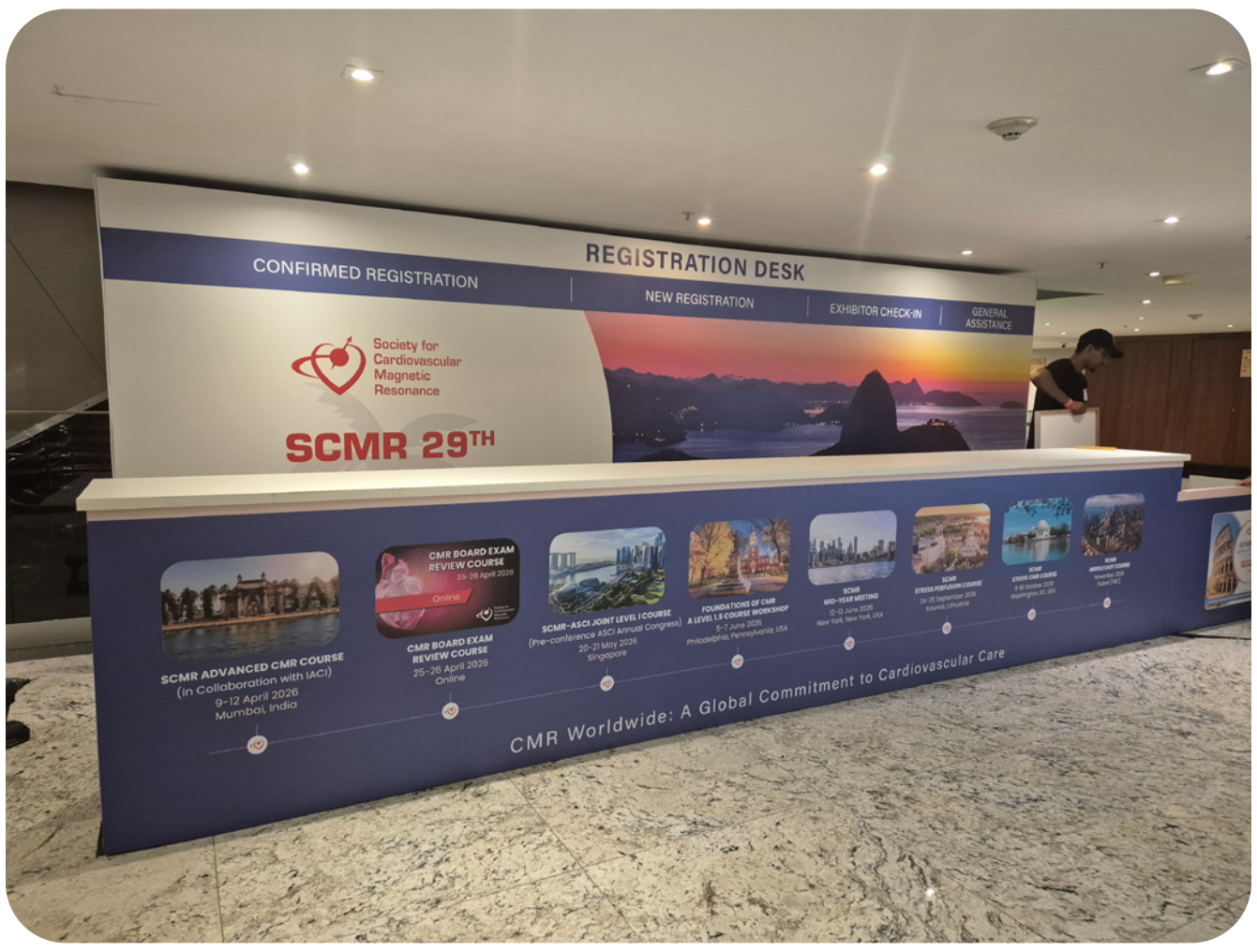

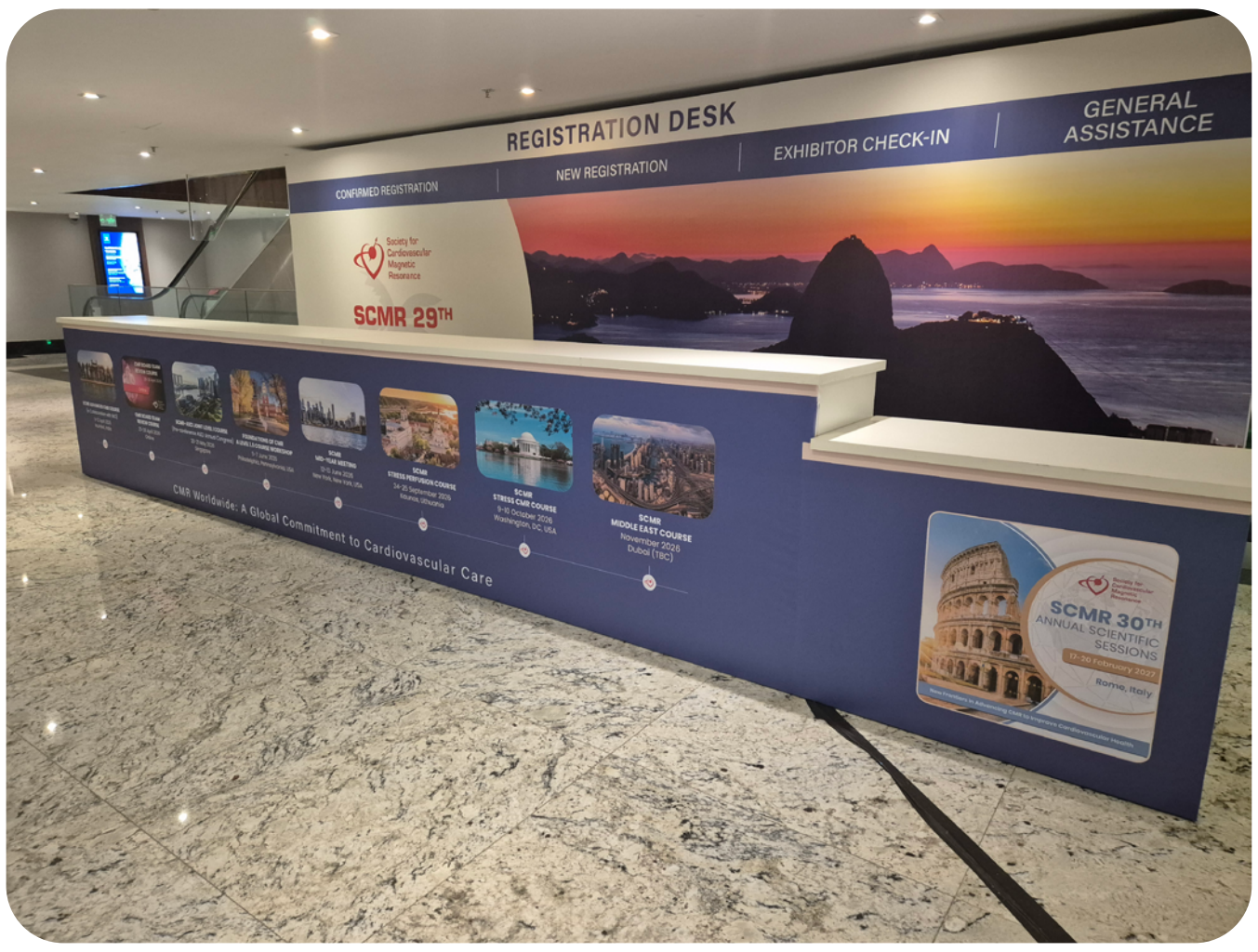

68 Meterboards Designed for SCMR 2026

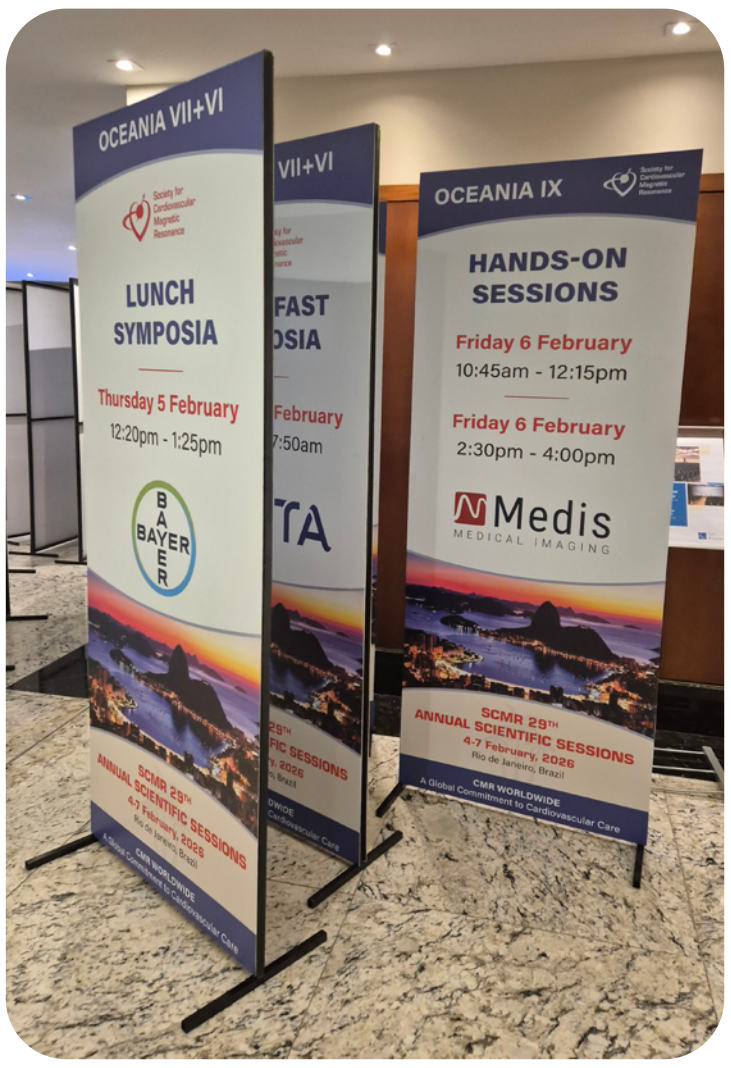

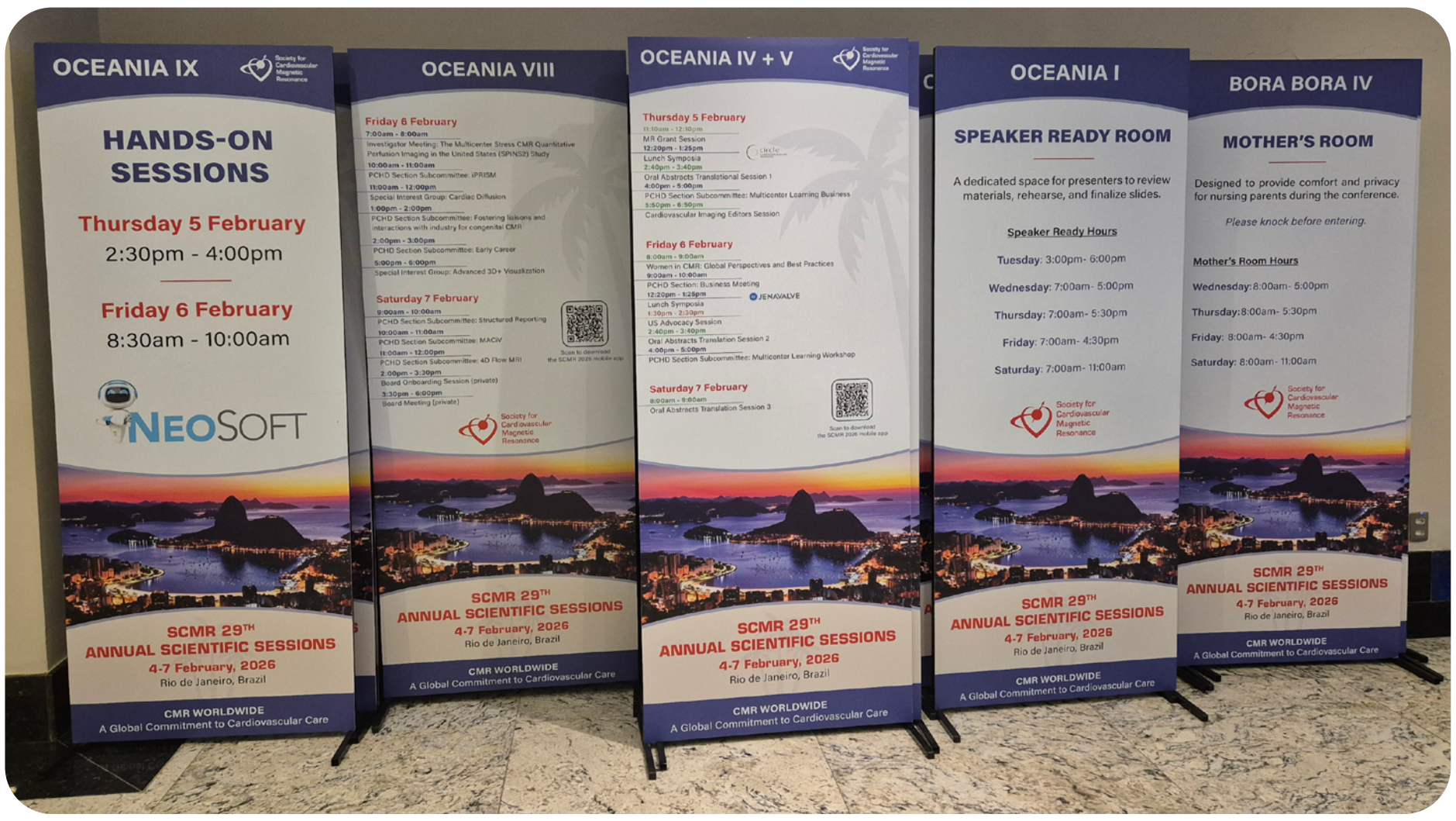

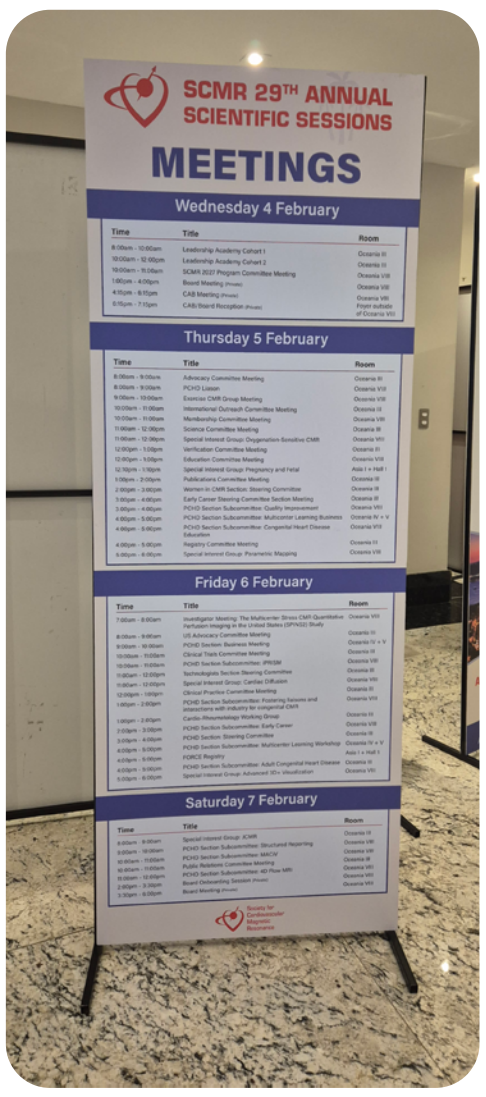

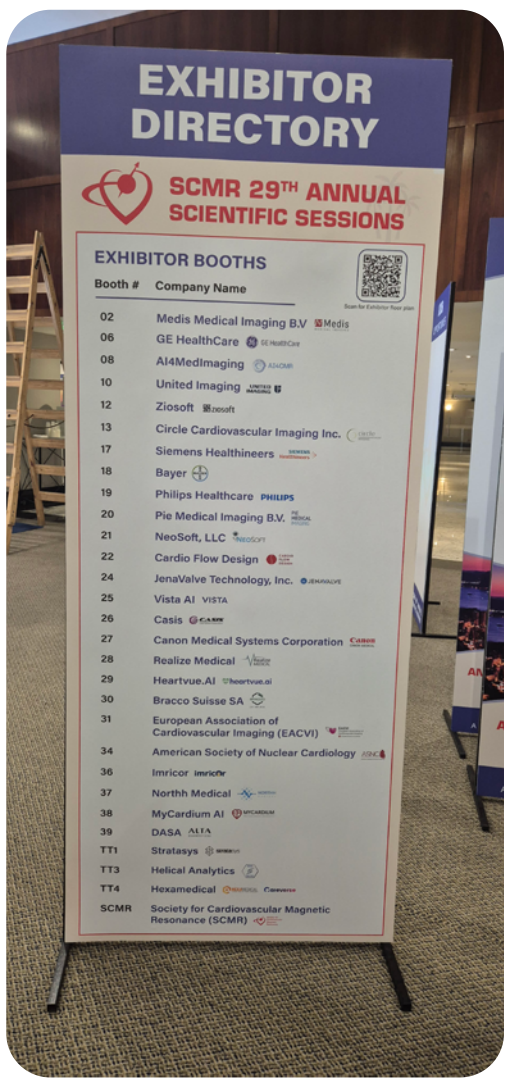

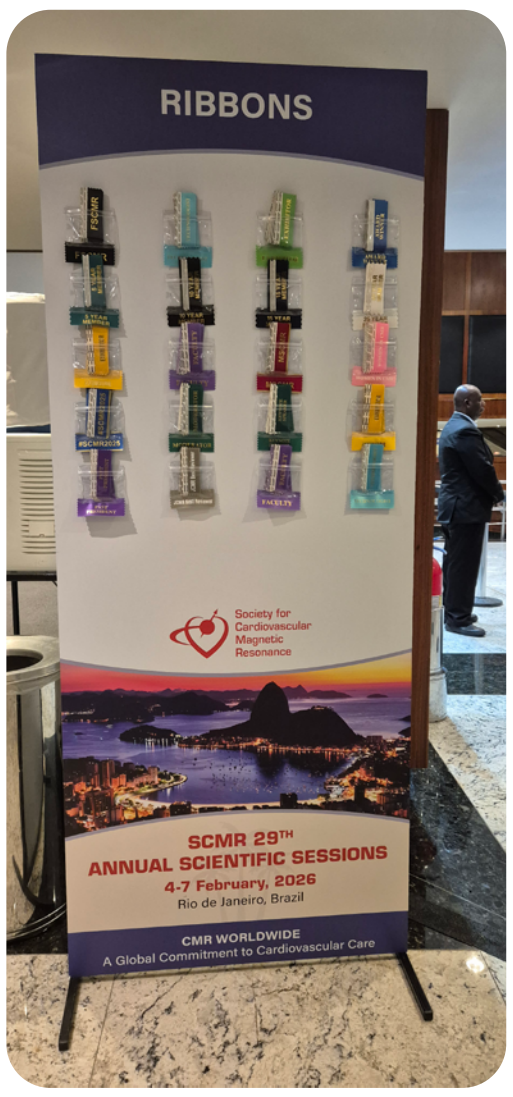

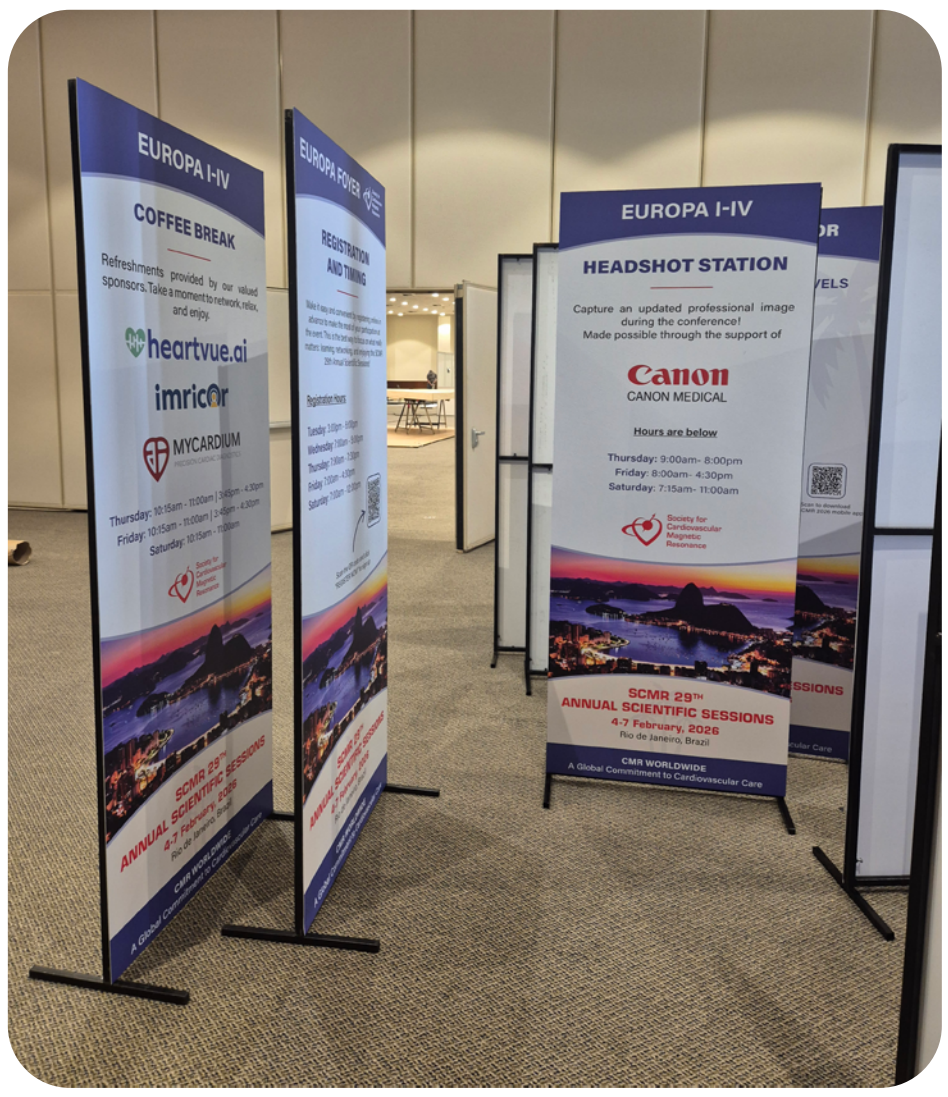

MARKETING & COMMUNICATION
ON SITE

Illuminated 3D letters

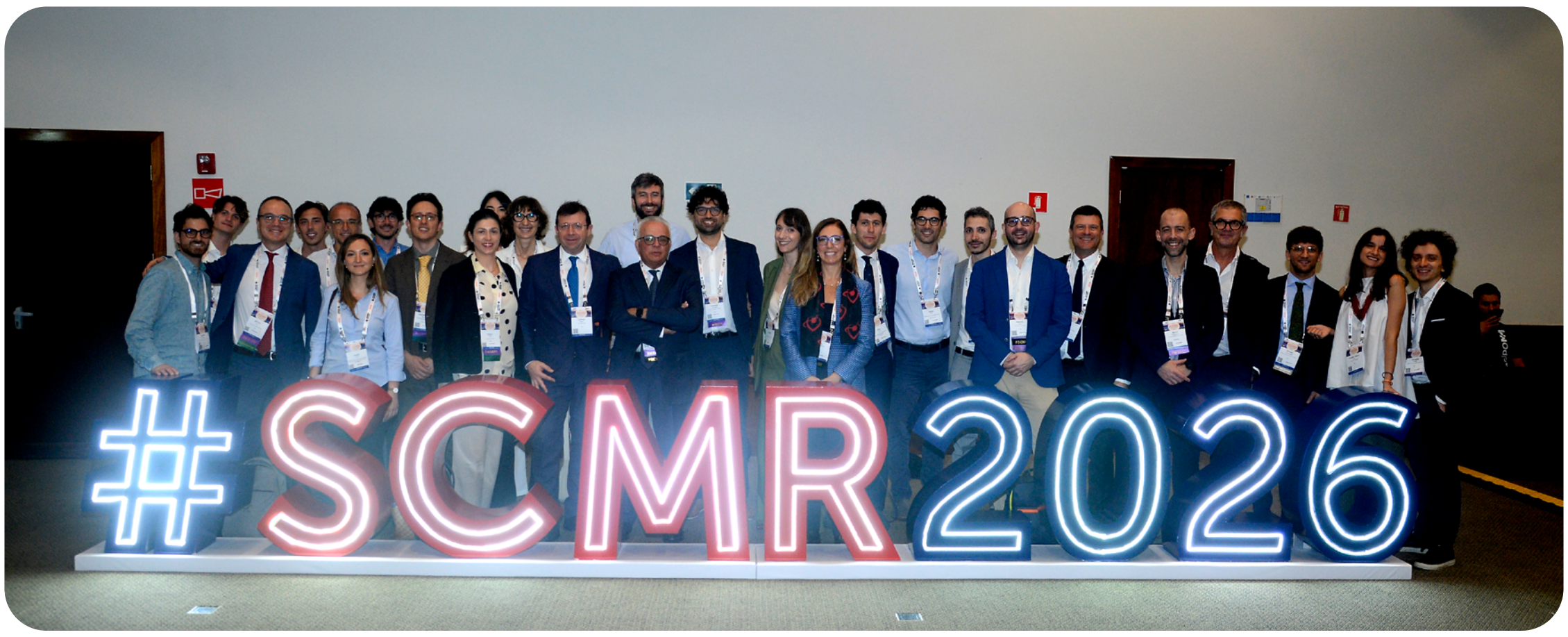

SCMR Booth

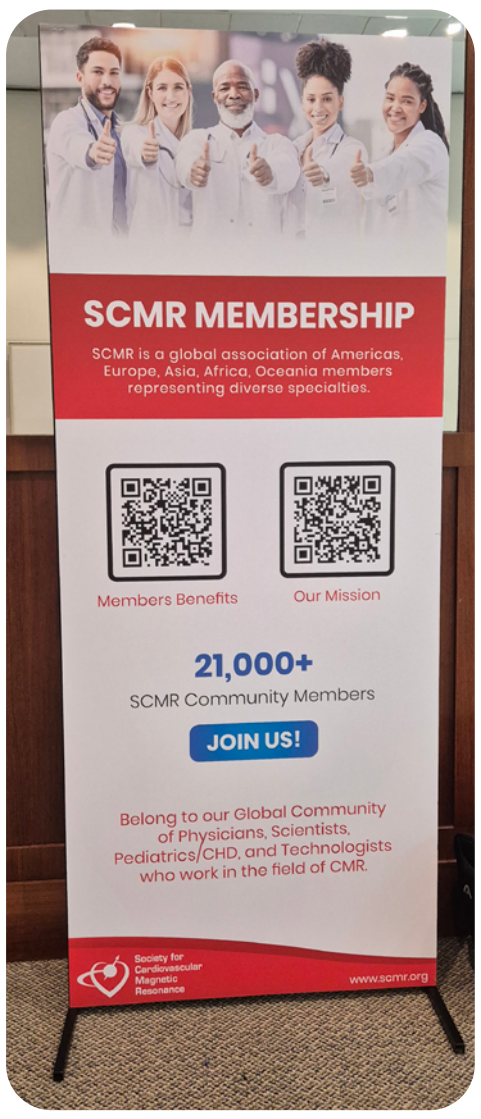

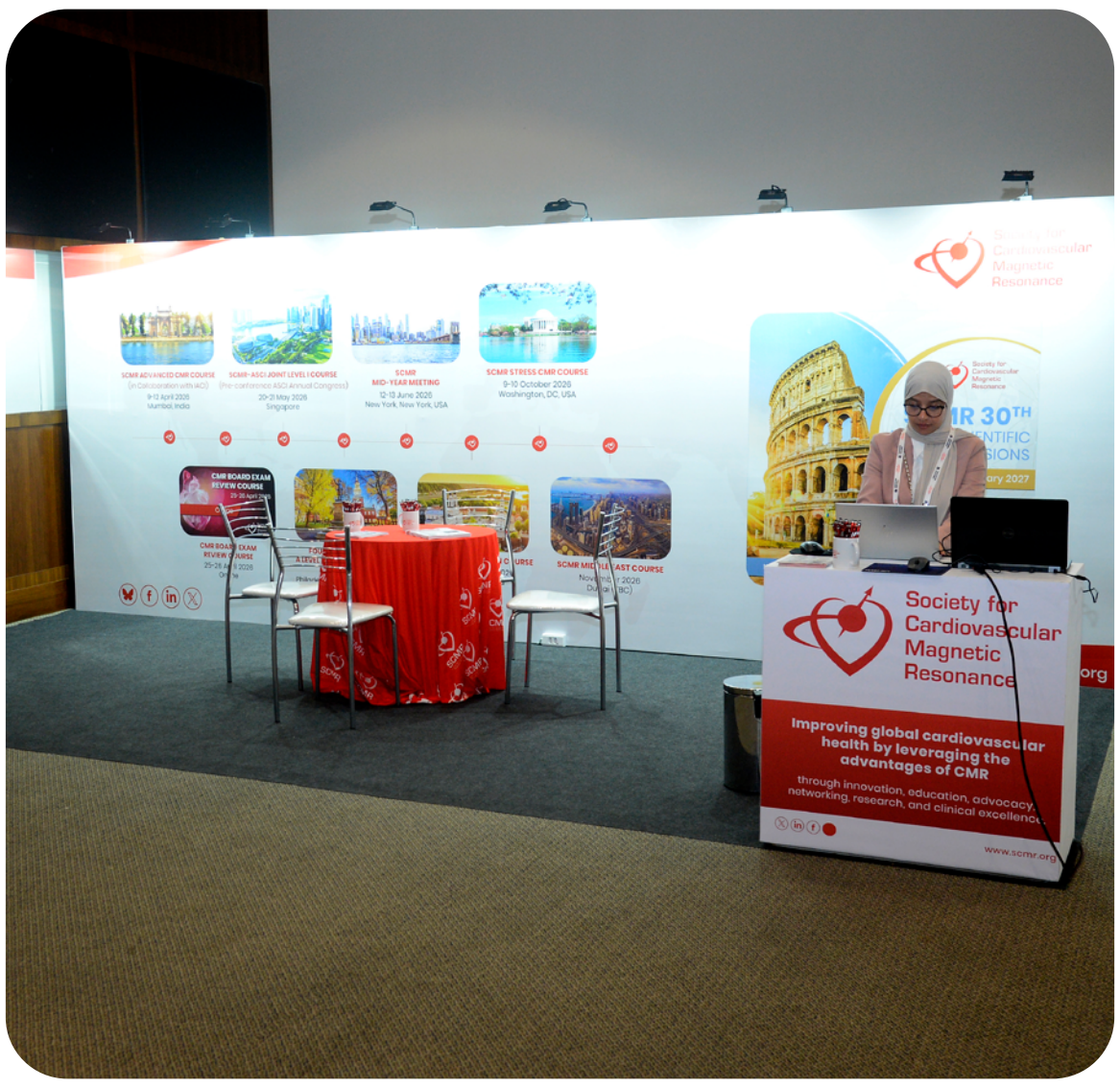

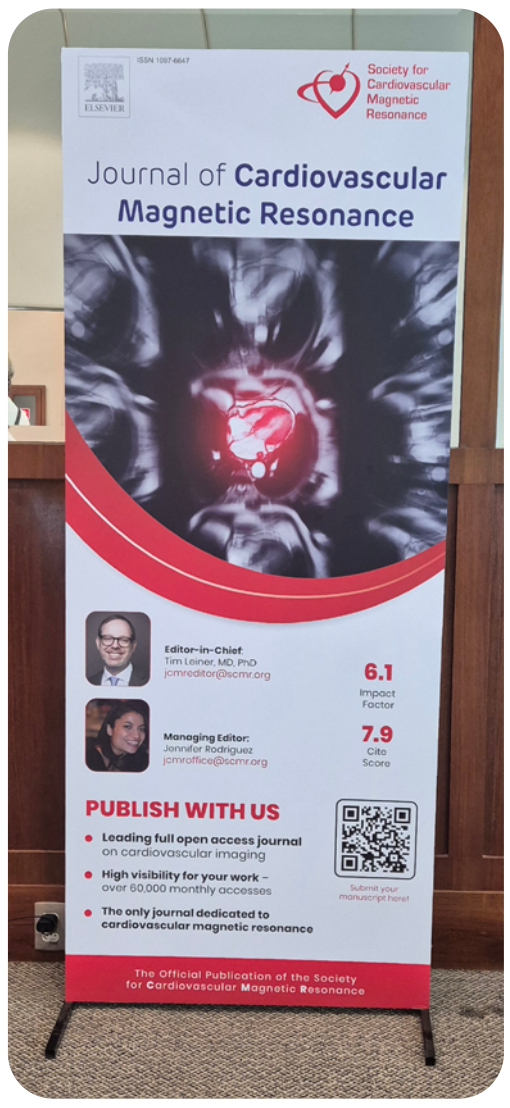

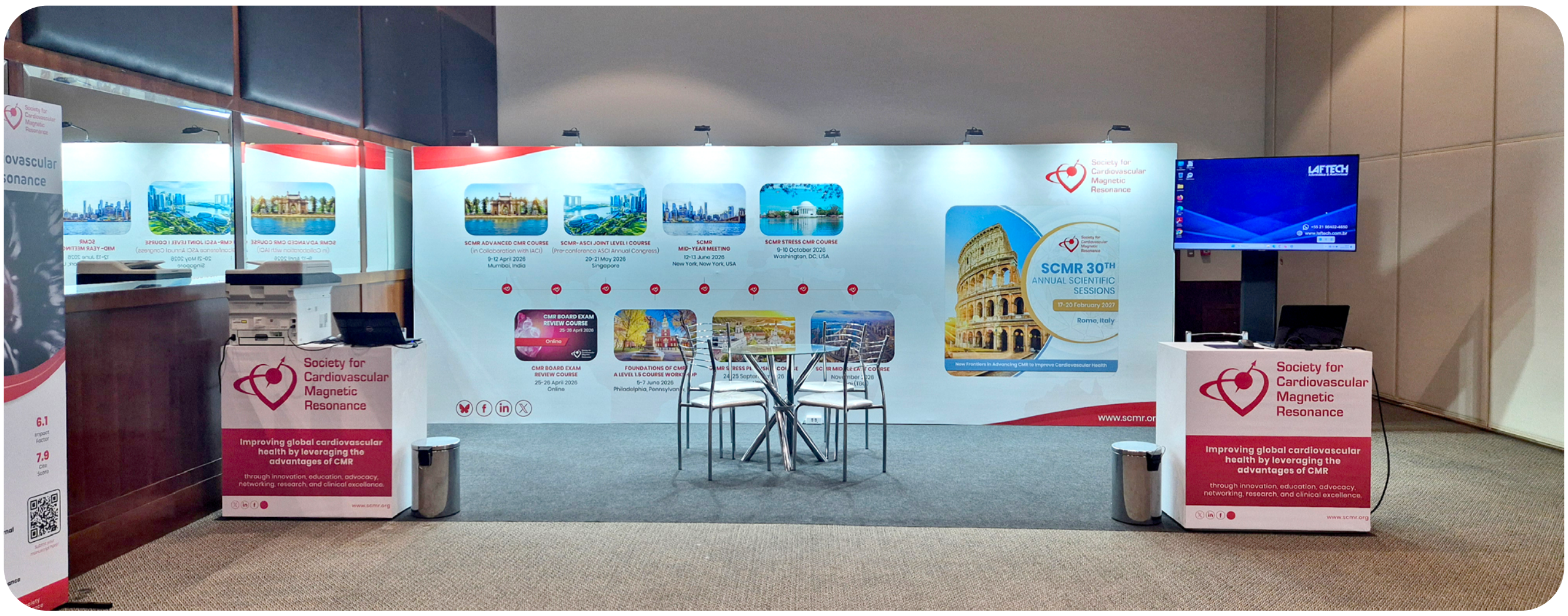

MARKETING & COMMUNICATION
ON SITE

Exhibition Hall

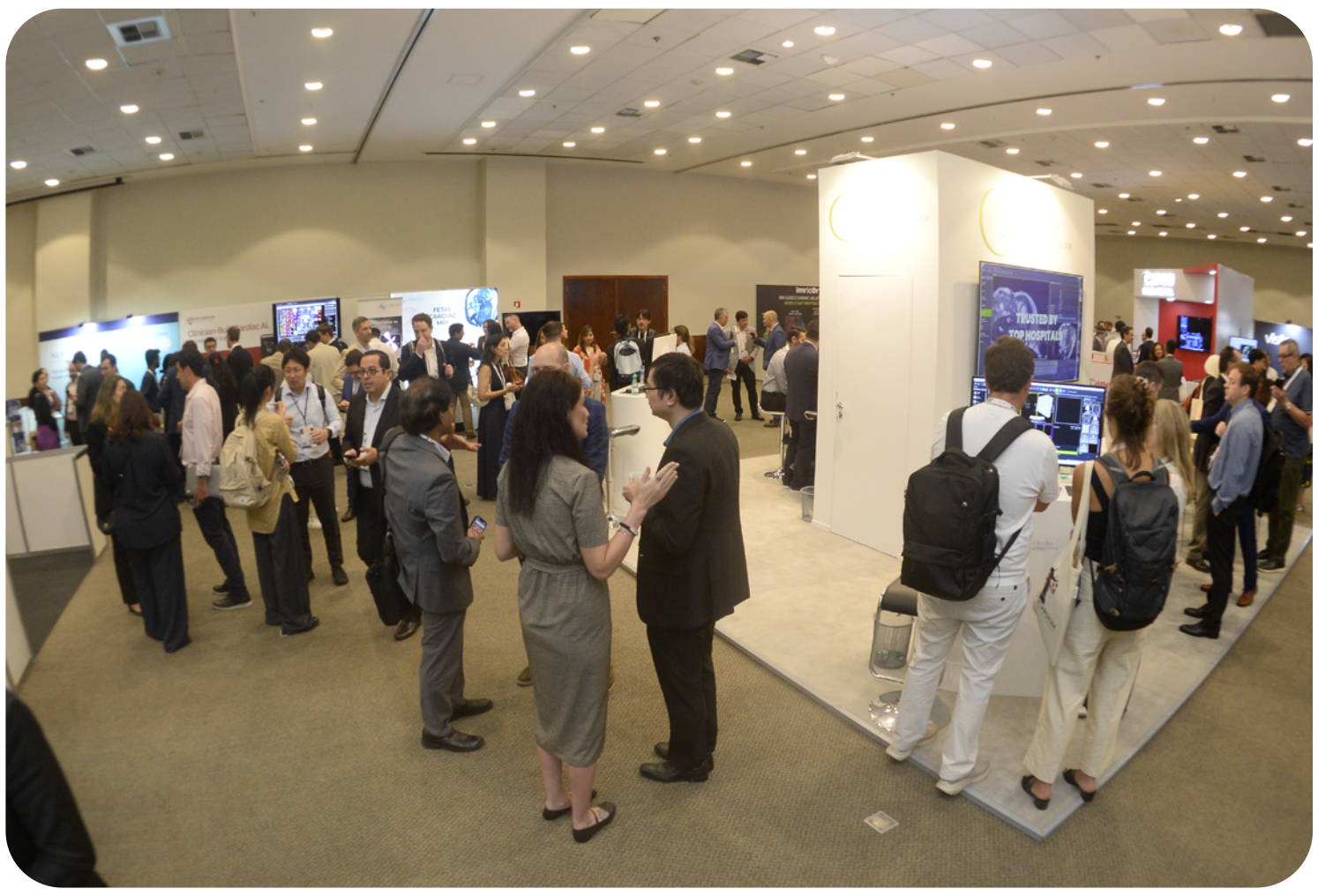

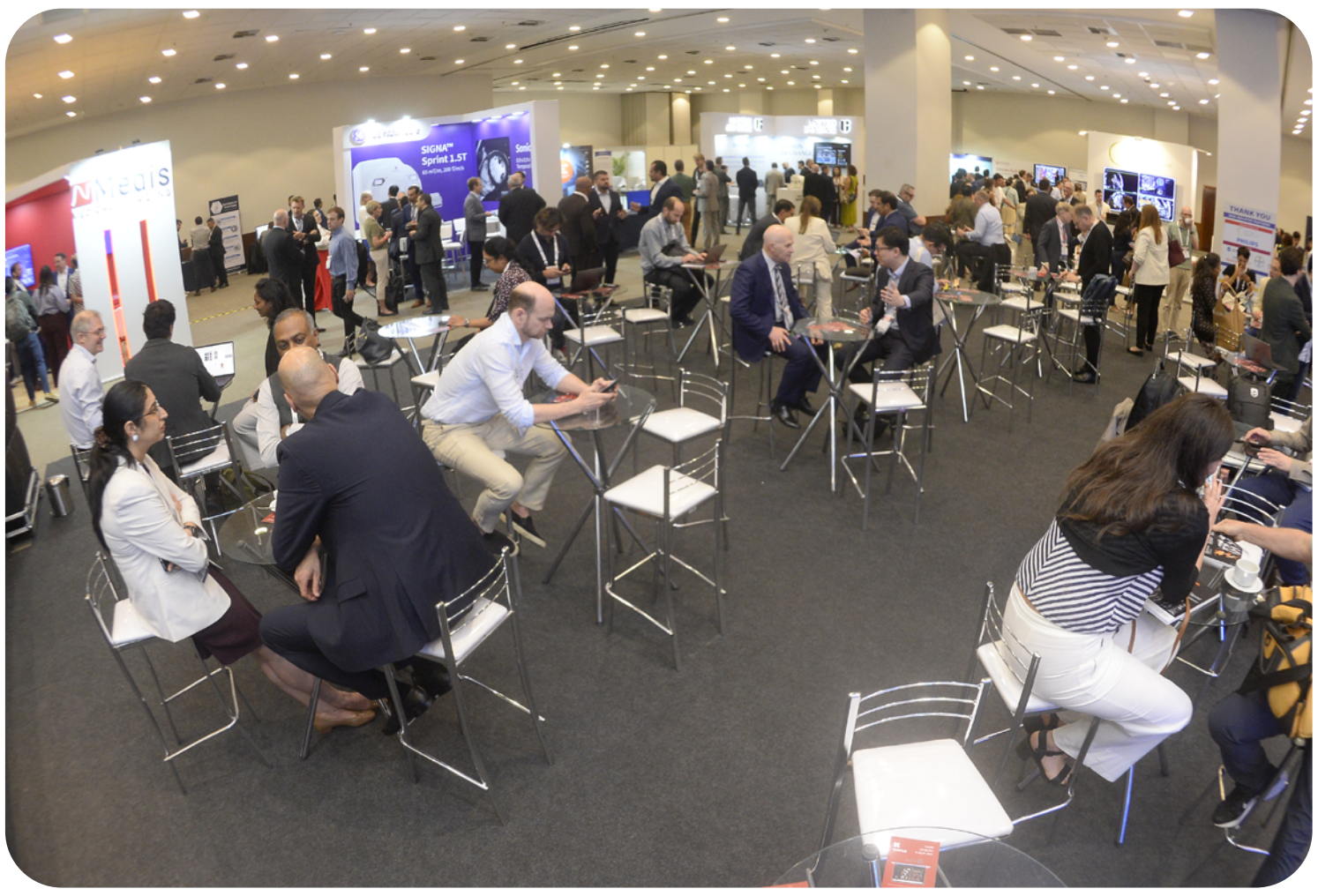

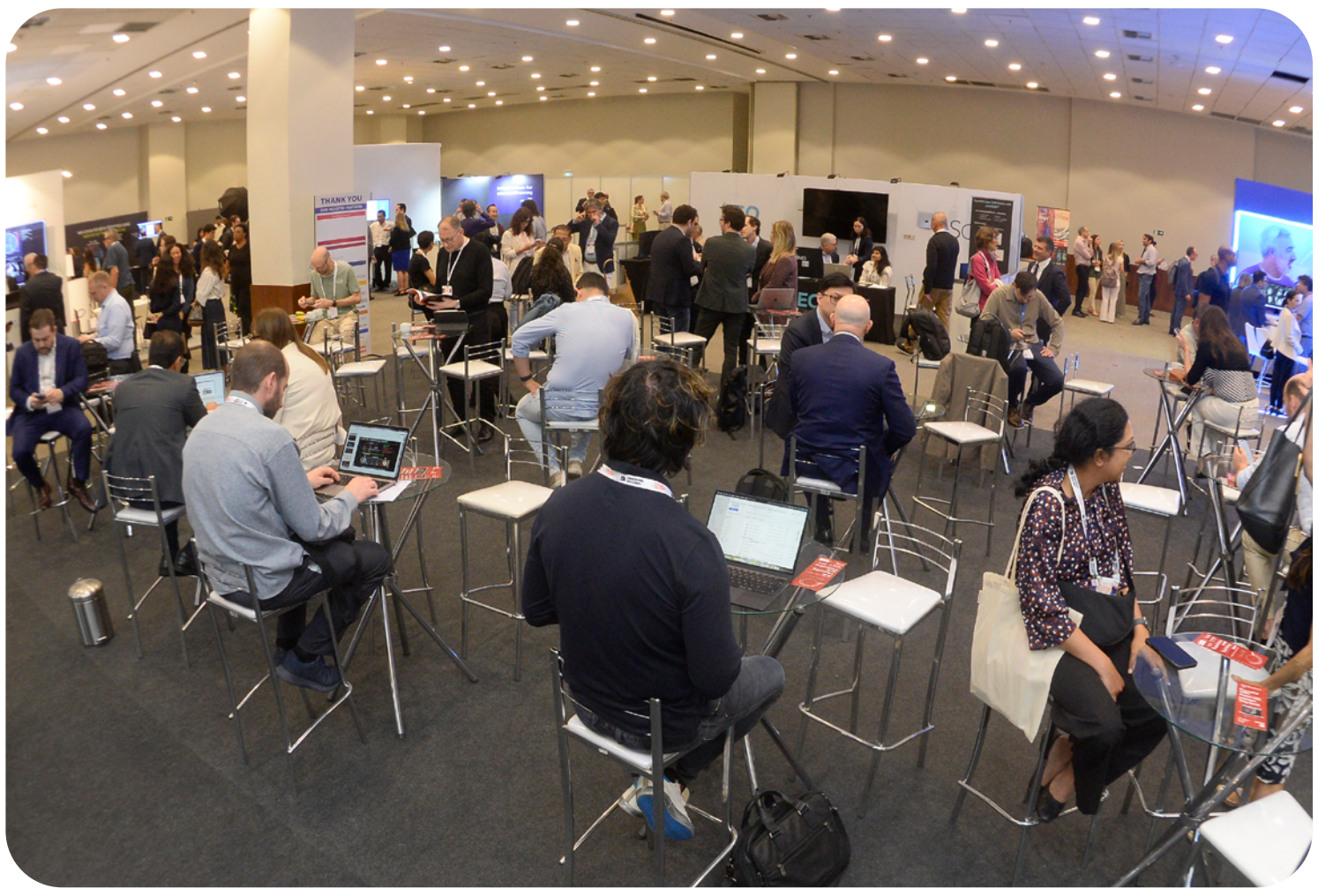

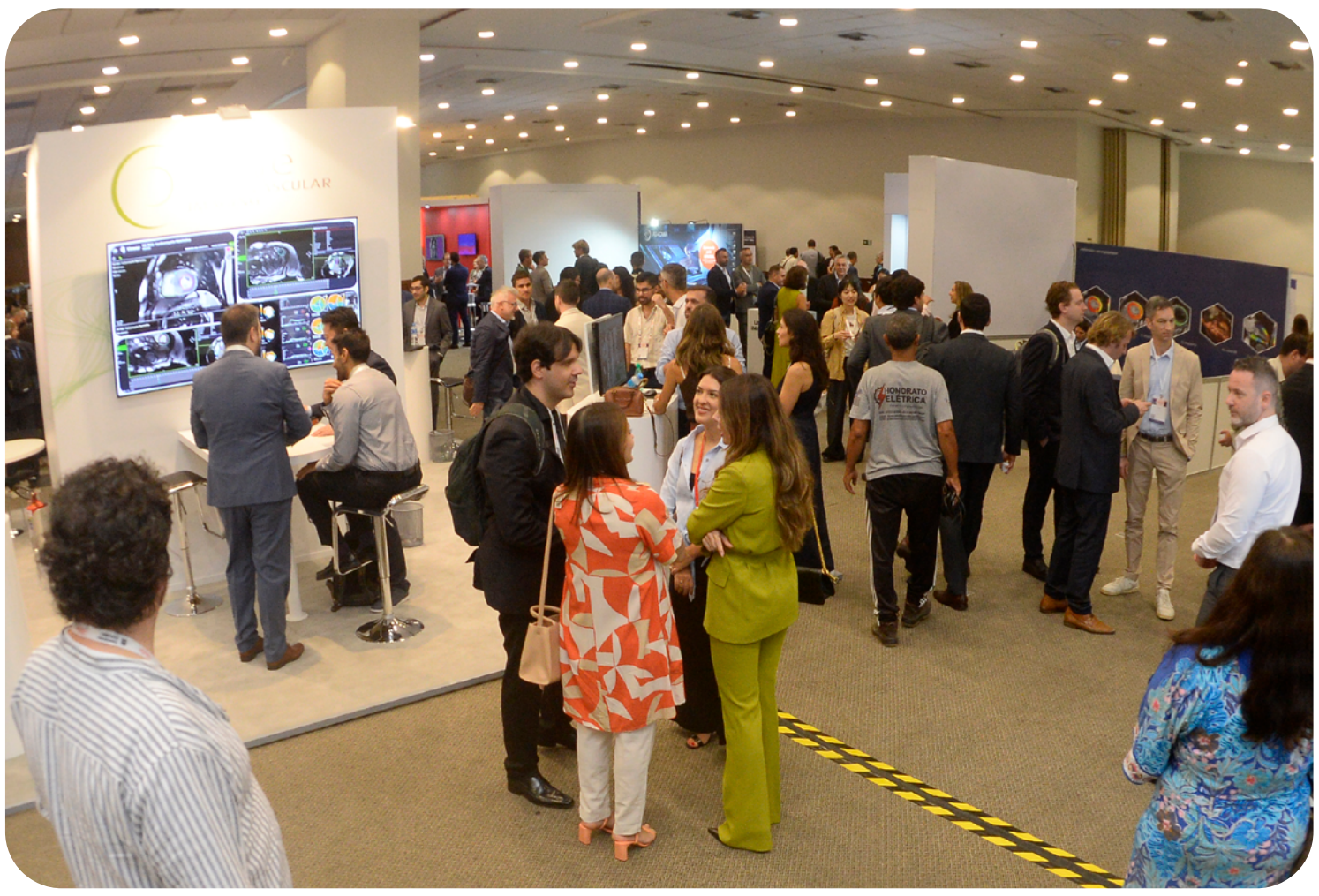

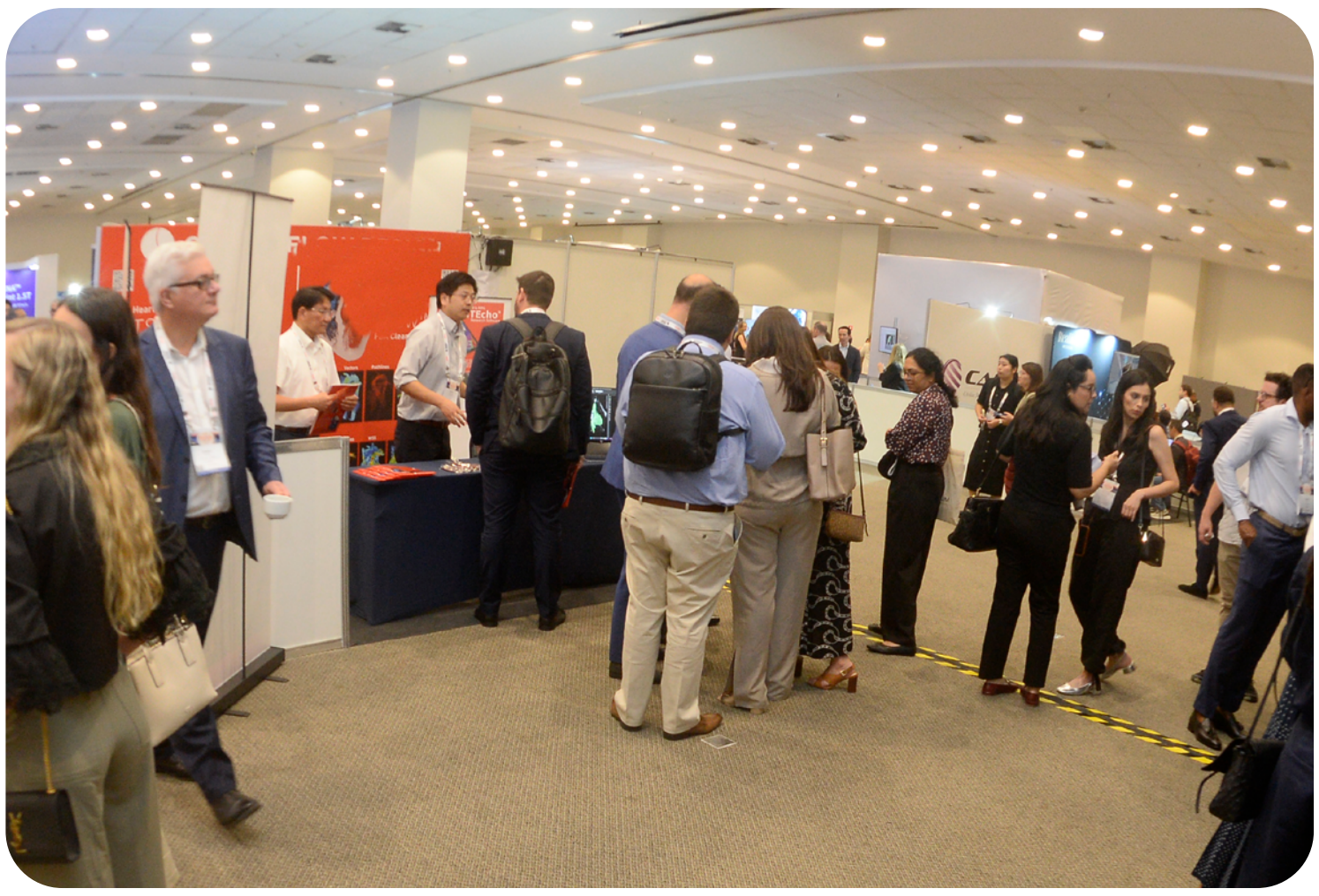

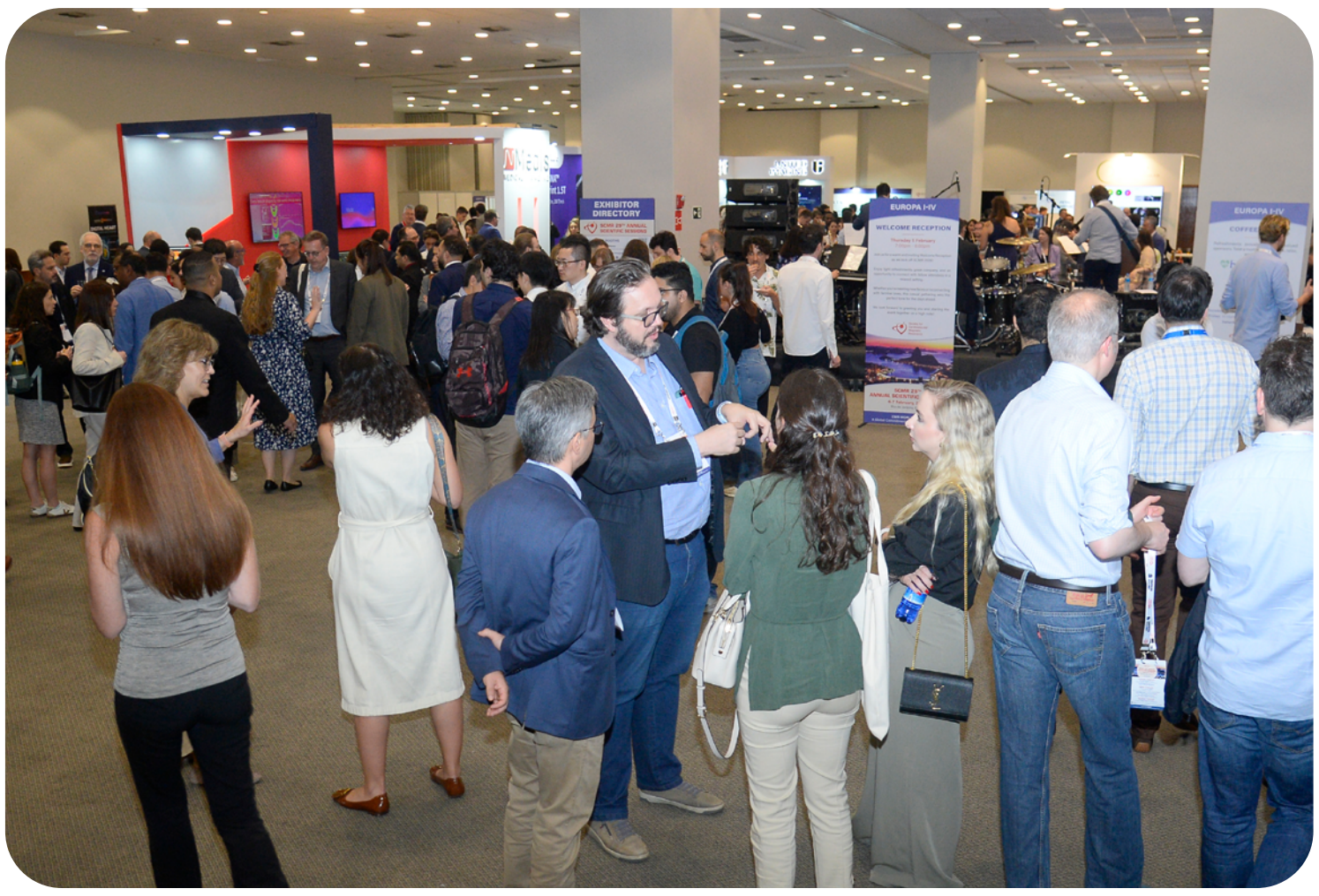

MARKETING & COMMUNICATION
ON SITE

On-Site Informational Signage

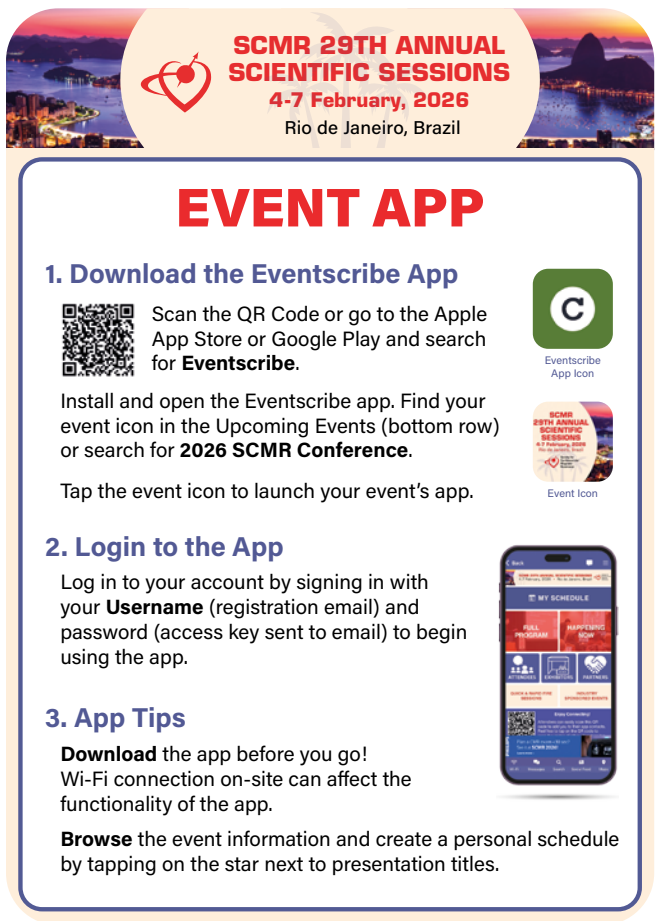

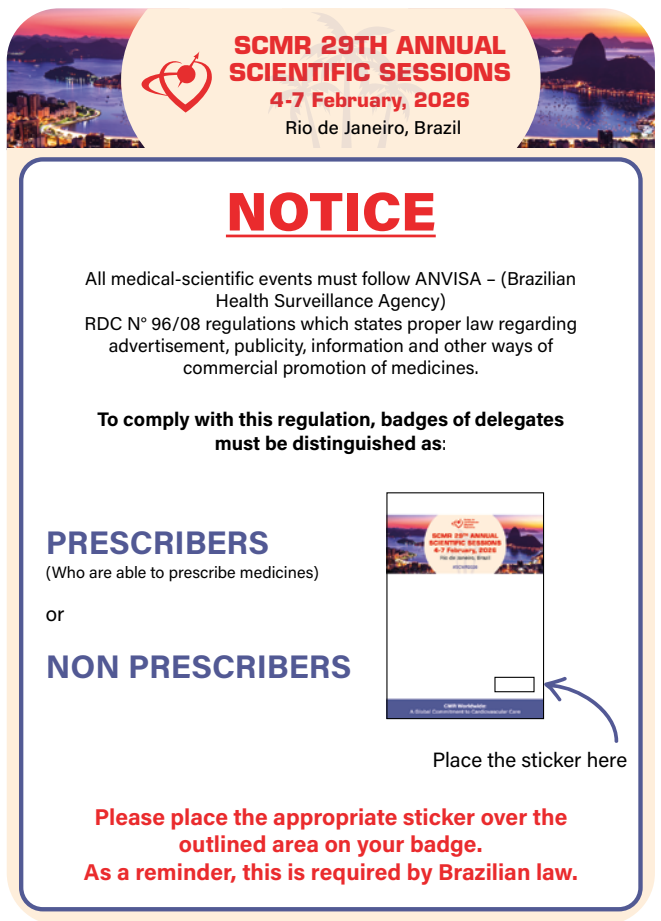

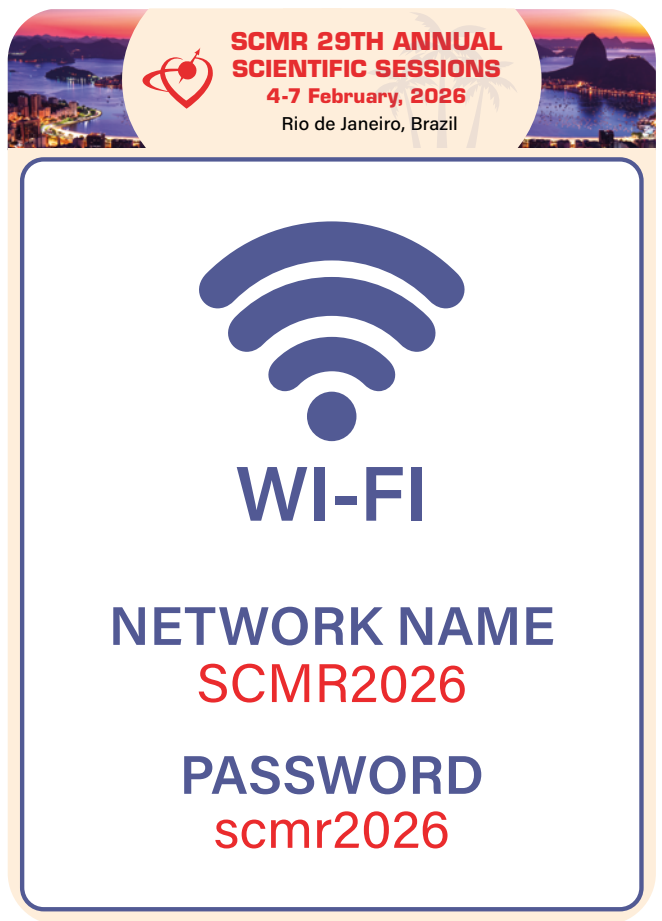

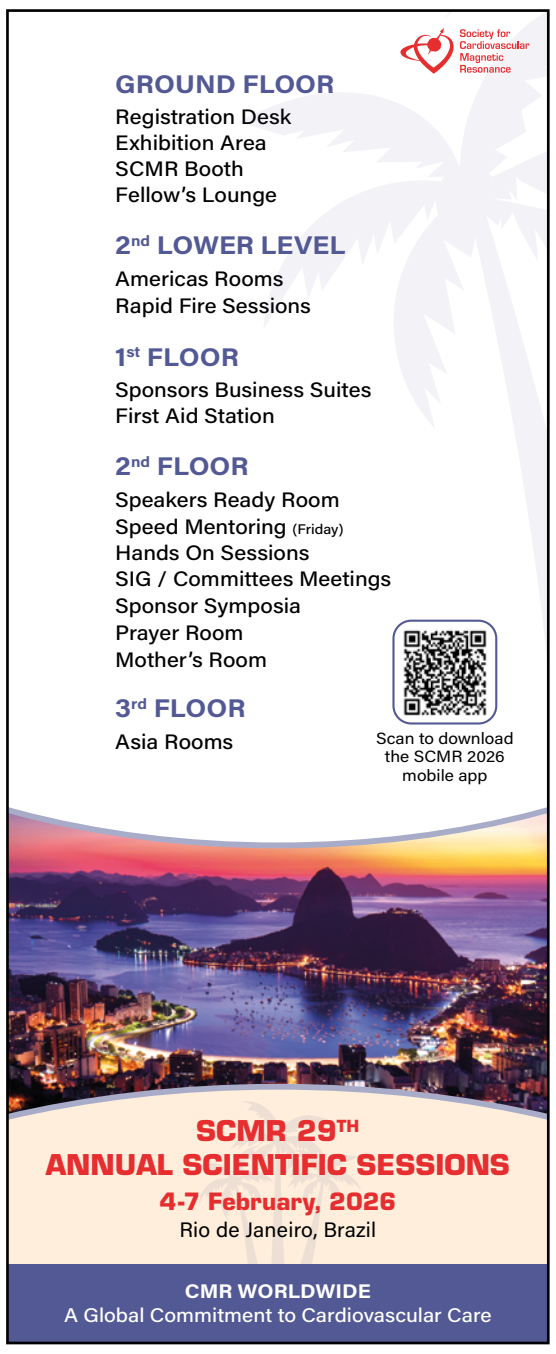

LED video wall

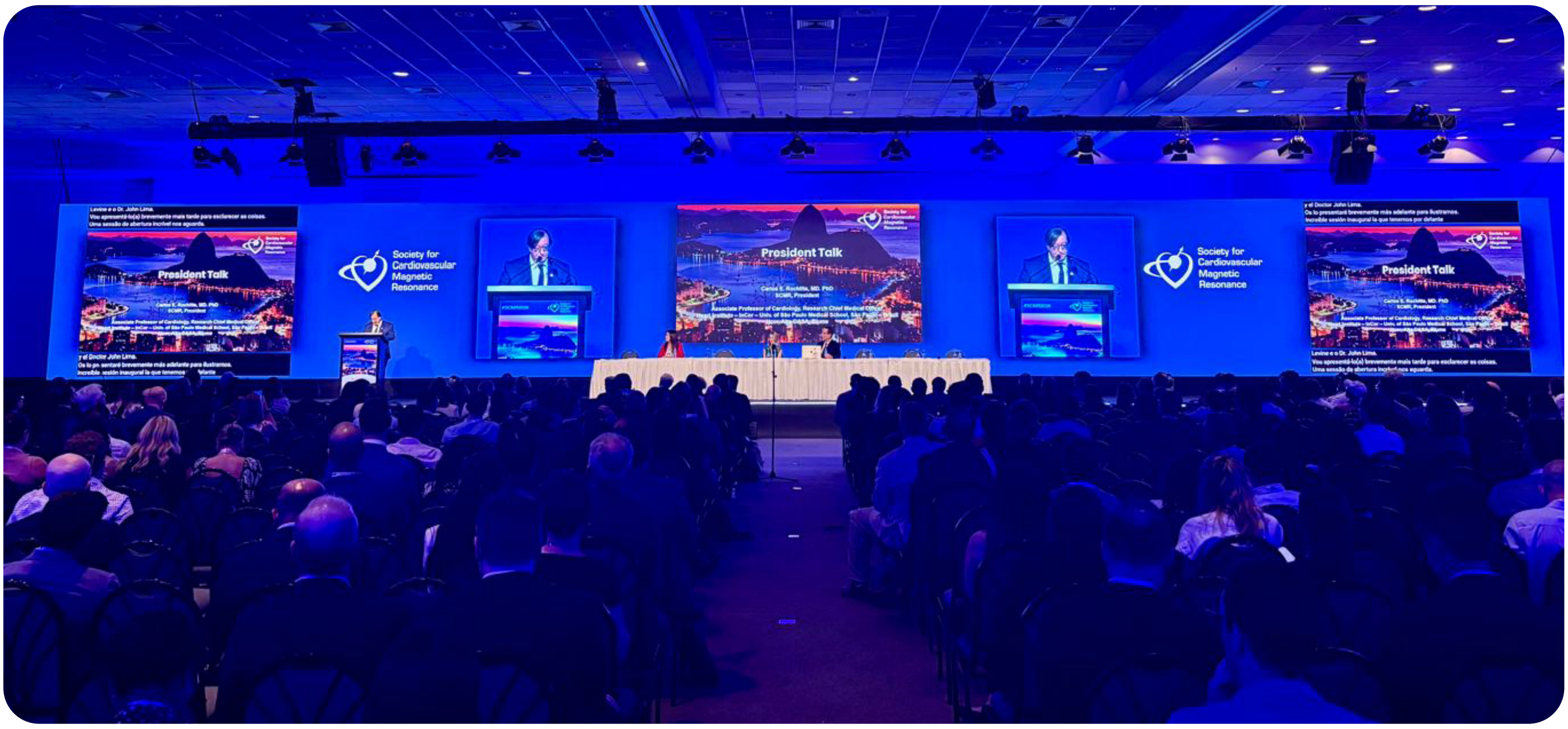

ACKNOWLEDGEMENTS

The Society for Cardiovascular Magnetic Resonance (SCMR) would like to express its sincere appreciation to all those whose dedication, collaboration, and commitment contributed to the success of the SCMR 2026 Annual Scientific Sessions.

Special thanks are extended to the SCMR staff — Chiara Bucciarelli-Ducci, Sophie Squarta, Ashley Duffy, Dale Gibbons, Halima Morchid, Stephanie Moyer, and Chelsea Smart for their professionalism, coordination, and continuous efforts in the planning, organization, and delivery of our 29th Annual Scientific Sessions. SCMR also acknowledges the support of Charlotte Riche-Martin, Marketing & Communications Consultant.

Finally, the Society extends its sincere gratitude to the entire SCMR community, including its members and attendees, whose participation and continued commitment make SCMR 2026 a dynamic and impactful forum for advancing cardiovascular magnetic resonance worldwide.

SCMR 2027

SEE YOU IN ROME NEXT YEAR!

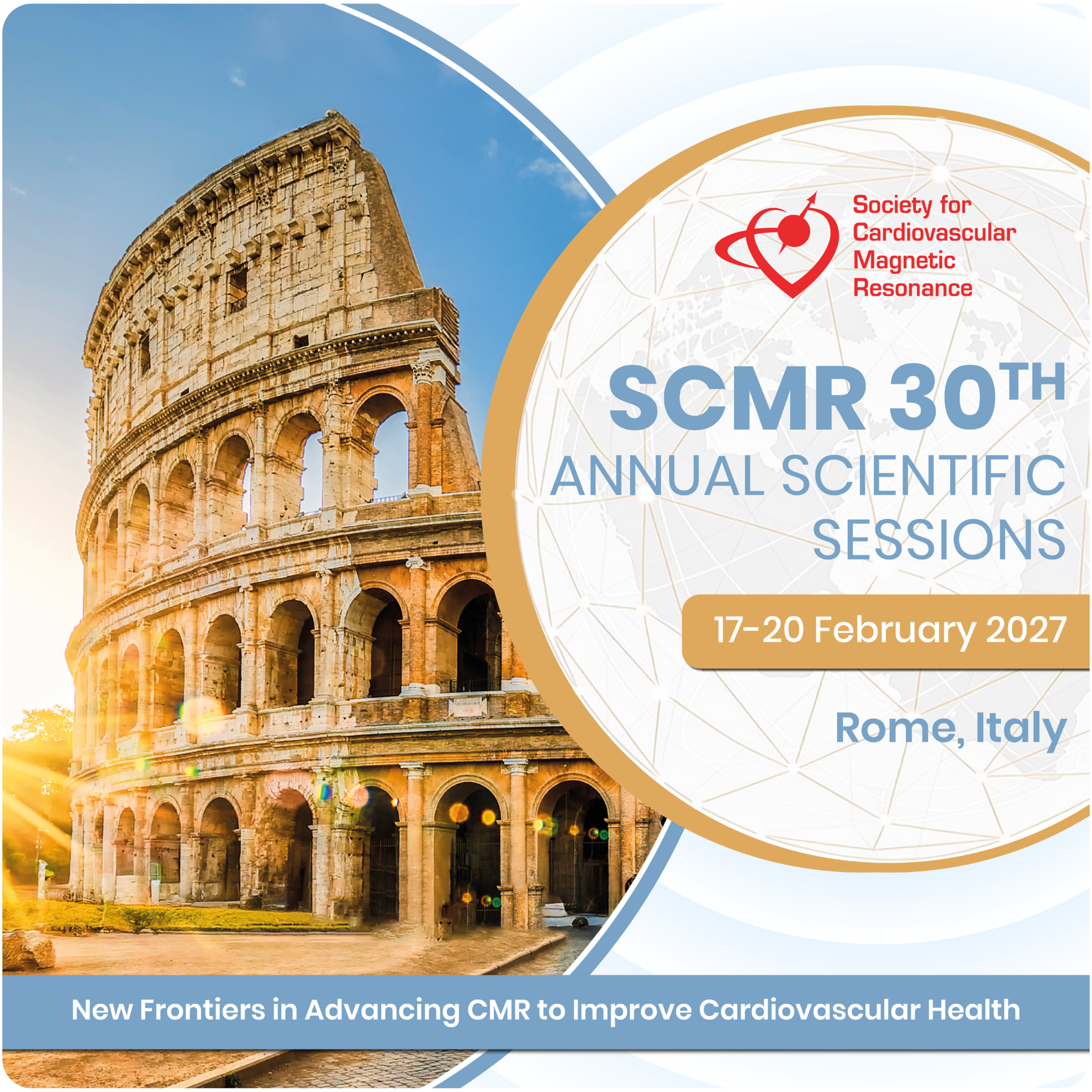

Society for Cardiovascular Magnetic Resonance

1061 East Main Street Ste. 300, East Dundee, IL 60118 USA
Telephone: +1-847-752-4657
E-mail: hq@scmr.org
www.scmr.org
