# Supplementary material for: Um Marco na Internacionalização da Ressonância Magnética Cardiovascular e um Legado para o Brasil e a América Latina
Source: Arq Bras Cardiol. 2026 Sep 21;123(9):e20260568. [Article in Portuguese] doi: 10.36660/abc.20260568 (PMC13649070; doi:10.36660/abc.20260568)
Supplement: Translation

# Translation: A Milestone in the Globalization of Cardiovascular Magnetic Resonance and a Legacy for Brazil and Latin America

**DOI:** 10.36660/abc.20260568i

**Authors:** Carlos Eduardo Rochitte, Clério Azevedo, Juliano Lara Fernandes

**Affiliations:** 1 Brazil Instituto do Coração do Hospital das Clínicas da Faculdade de Medicina da Universidade de São Paulo, São Paulo, SP – Brazil; 2 Brazil Hospital do Coração (Hcor), Associação Beneficente Síria, São Paulo, SP – Brazil; 3 Brazil Alta/DASA Medicina Diagnóstica, São Paulo, SP – Brazil; 4 Brazil Blume Medicina Diagnóstica, São Paulo, SP – Brazil; 5 USA Duke University, Durham – USA; 6 Brazil RDC Medicina Diagnóstica, Campinas, SP – Brazil

**Keywords:** Cardiology, Radiology, Congresses as Topic, International Cooperation

## Abstract

**Central Illustration:** : A Milestone in the Globalization of Cardiovascular Magnetic Resonance and a Legacy for Brazil and Latin America

There are moments in the life of a scientific society that transcend the organization of a successful meeting. They become milestones that redefine identity, expand mission, and create a lasting legacy for the field. The 29th Annual Scientific Sessions of the Society for Cardiovascular Magnetic Resonance (SCMR), held in Rio de Janeiro from February 4th to 7th, 2026, was such a moment. For the first time in its history, the leading international society dedicated to cardiovascular magnetic resonance (CMR) held its annual meeting outside North America and Europe. Its theme, CMR Worldwide: A Global Commitment to Cardiovascular Care, became more than a conference slogan; it became an institutional statement about the future of the specialty.

The meaning of SCMR Rio 2026 lies not only in the fact that it took place in Brazil. Its deeper meaning is what it demonstrated: scientific excellence, geographic inclusion, workforce development, and multidisciplinary collaboration are not competing goals. They are complementary dimensions of the same mission. By bringing the annual meeting to Latin America, SCMR did not simply move its venue. It brought education, science, mentorship, and leadership closer to a region with a high burden of cardiovascular disease, substantial scientific capacity, and persistent inequities in access to advanced cardiovascular imaging.

The final numbers tell a compelling story (Supplementary Material). SCMR 2026 brought together more than 1,600 participants from 73 countries, including approximately 600 first-time attendees. Brazil ranked second among all countries in attendance, behind only the United States, and Latin America achieved its highest representation in the history of the Society. The scientific program received 794 abstract and case submissions, of which 655 were accepted, representing 44 countries. The program included 80 scientific sessions, six preconference courses or workshops, eight innovation track sessions, eight advanced hands-on workshops delivered across 14 sessions, all of which reached full capacity, and 34 sessions with live Portuguese or Spanish translation. These are not merely administrative metrics. They show the measurable effect of reducing geographic, linguistic, and economic barriers (Supplementary Material).

The presence of world leaders at SCMR 2026 in Rio de Janeiro was one of the major highlights of the congress. Professor João Lima, from Johns Hopkins University, opened the meeting with an unforgettable lecture on how CMR and SCMR have advanced together over the years in an extraordinary journey of science, discovery, and global impact: “CMR and SCMR Journey: Science, Discovery, and Global.”

Also during the opening session, Dr. Steve Levine, one of the world’s leading experts in digital twin technology, delivered a deeply moving lecture, weaving a personal story involving his daughter with the evolution of this technology, in “The Living Heart: How Digital Twins Are Transforming Cardiovascular Medicine”.

In the closing session, Professor Raymond Kim, from Duke University, offered the audience a brilliant reflection on what the next major paradigm shift in CMR may be. Naturally, artificial intelligence played a central role, but, as always in his remarkable presentations, he also brought a broader and inspiring vision for the future of the field: “What is the Next Paradigm Shift for CMR”

Finally, Professor Christopher Kramer, then President of the American College of Cardiology, closed SCMR 2026 on a high note, addressing the meaning of CMR and SCMR for global cardiovascular health in his lecture “CMR and the Future of Global Cardiovascular Health”.

Latin American participation was particularly meaningful. Approximately 300 participants came from Latin America, the highest number ever recorded for the region at an SCMR annual meeting^1^ (Supplementary Material). Forty-five Latin American societies formally supported SCMR 2026, and a dedicated Latin America preconference brought together professionals from multiple countries. These elements show that Rio 2026 was not a temporary visit by SCMR to Latin America. It was the result of years of regional development and a collective commitment to building lasting scientific infrastructure.

Brazil occupies a distinctive place in this history. Over the past two decades, Brazilian CMR has evolved steadily: first incorporating international expertise, then training specialists, developing guidelines led by the Brazilian Society of Cardiology-SBC,^2^ participating in multicenter studies, and ultimately helping train professionals from other Latin American countries. This growth occurred in a multidisciplinary environment in which cardiologists and radiologists jointly contributed to the consolidation of the field. The Brazilian Society of Cardiology, the Brazilian College of Radiology and Diagnostic Imaging, the São Paulo Society of Radiology, and regional societies have all contributed to guidelines, educational programs, scientific meetings, and quality initiatives in cardiovascular imaging.

Recent data presented by Brazilian leaders during SCMR 2026 illustrate both the achievements and the challenges ahead.^3^ Brazil has more than 200 million inhabitants, 27 federative units, and approximately 40 centers performing CMR. In 2024, approximately 15,000 CMR examinations were performed in the public system and approximately 44,000 in the private sector. These numbers reveal both the expansion of the modality and the persistent inequity of access. Professionals and centers remain concentrated in the South and Southeast regions, where private practice and technological infrastructure are more available. The challenge for Brazil, therefore, is not only to increase the number of examinations but to improve quality, geographic distribution, and equity of access.

In this context, hosting SCMR in Rio had both symbolic and practical value. For many young cardiologists, radiologists, physicists, technologists, and researchers from Latin America, participation in a major international CMR meeting became possible for the first time. The presence of approximately 600 first-time attendees is one of the most important signals of the impact of location. Science grows not only through publications and guidelines. It grows when new professionals meet a scientific community, find mentors, present their first work, and begin to see themselves as part of an international network.

The structure of SCMR 2026 was designed with this intention. Differential registration fees according to country income level, live translation, travel grants, joint sessions with sister societies, dedicated regional programming, and hands-on workshops formed a strategy of active inclusion. Sixty-one travel grants were awarded, and 53 grant recipients attended the meeting. The program included 774 faculty, moderator, and panelist roles distributed across 28 countries, including 56 roles from Brazil. Latin America was not present only as an audience. It was present as a source of scientific leadership.

Education is another essential component of the Rio legacy. Scientific meetings inspire; training and certification sustain excellence. Since its earliest years, SCMR has recognized that safe expansion of CMR requires clear standards of competence. In 2000, SCMR published its first credentialing guidelines in CMR, defining levels of training and experience. Subsequent documents, including the 2007 and 2018 training guidelines and the 2024 competency-based curriculum, created an internationally recognized framework for developing professionals capable of acquiring, interpreting, and reporting CMR examinations with quality.

This evolution is especially relevant for developing regions. Certification should not be understood as a corporate barrier. It should be understood as a tool for democratizing quality. In countries with heterogeneous infrastructure and training, certification provides an objective reference for physicians, institutions, policymakers, and patients. By defining minimum competencies for independent practice, including clinical knowledge, acquisition protocols, cardiovascular interpretation, safety, stress pharmacology, recognition of relevant extracardiac findings, and participation in quality improvement, certification helps transform enthusiasm into sustainable practice.^4-7^

The 2024 SCMR competency curriculum is particularly important because it was designed to be globally relevant and applicable to trainees from both cardiology and radiology backgrounds.^8^ This vision is central to CMR. The field was born and matured at the intersection of disciplines. Cardiologists bring expertise in physiology, cardiovascular disease, risk stratification, and clinical decision-making. Radiologists bring expertise in imaging physics, safety, broad anatomic interpretation, and extracardiac findings. Physicists, engineers, technologists, data scientists, and translational researchers push the technical and scientific limits of the field. CMR is stronger when these competencies work together.^9-11^

This principle was recently reaffirmed in an institutional letter from SCMR to the international cardiovascular imaging community. The Society stated that it supports both cardiologists and radiologists who have achieved the required competence to independently report and sign clinical CMR studies. The essential criteria should be training, competence, experience, and clinical judgment, rather than specialty boundaries. The letter also emphasized that an ideal CMR service includes collaboration between cardiologists and radiologists, with availability for second opinions in complex cardiovascular or extracardiac findings. This vision is particularly important for Brazil and Latin America, where the expansion of CMR requires combining competencies rather than creating barriers.

Multidisciplinary collaboration should also guide the relationship among scientific societies. The Brazilian experience presented during SCMR 2026 showed that guidelines, courses, and meetings in cardiovascular imaging have often been built through interaction among cardiology and radiology societies. This experience can serve as a model for other countries. Rather than reproducing professional territorial disputes, Latin America has an opportunity to develop a collaborative model centered on quality, training, and patient access.

The impact on Brazilian cardiology must be understood in this broader context. CMR is now essential for the diagnosis and management of cardiomyopathies, myocarditis, congenital heart disease, coronary artery disease, valvular disease, cardiac masses, systemic diseases with cardiac involvement, and myocardial viability. In Latin America, its relevance is amplified by regional burdens such as Chagas cardiomyopathy and rheumatic heart disease, as well as the growing prevalence of heart failure, coronary artery disease, and cardio-oncology. Expanding access to high-quality CMR is not a technological luxury. It is a strategy to improve diagnosis, prognosis, and therapeutic decision-making for high-risk populations.

SCMR Rio 2026 also occurred during a period of transformation within the Society itself. In the same cycle, SCMR advanced toward a self-managed model, reducing dependence on an external management company and building its own operational team. This change, less visible to attendees than the historic meeting in Rio, is also highly consequential. A self-managed society can better align strategy, education, certification, advocacy, communication, and financial sustainability. Together, the first annual meeting outside North America and Europe and the transition to self-management represent complementary milestones: SCMR changed where it meets and how it organizes itself.

The legacy of Rio must therefore be measured over the coming years. It will be seen in new CMR centers, increasing numbers of certified professionals, Latin American multicenter studies, greater participation of early-career researchers in international meetings, regional guidelines, structured training programs, and stronger integration between cardiologists and radiologists. The success of a meeting lasts a few days. The success of a scientific movement lasts decades.

The *Arquivos Brasileiros de Cardiologia* has a central role in this process. As the leading cardiovascular journal in Brazil and one of the most important scientific voices in Latin America, ABC Cardiol can disseminate CMR evidence generated in the region, stimulate debate on access and quality, promote locally relevant guidelines, and connect the broader clinical community with the possibilities offered by advanced cardiovascular imaging. Publishing an editorial on SCMR Rio 2026 should not be seen as an institutional celebration, but as a record of a new chapter in Brazilian cardiology.

Rio de Janeiro demonstrated that scientific excellence is not determined by geography. When education, certification, multidisciplinary collaboration, and global inclusion are deliberately combined, science expands without losing rigor. CMR does not belong to a country, a specialty, or a professional group. It belongs to the patients who benefit from it. SCMR Rio 2026 was far more than a scientific meeting. It demonstrated that the international CMR community is ready for a truly global phase, in which Brazil and Latin America are no longer at the periphery but at the center of building the future of cardiovascular imaging.

The true legacy of SCMR Rio 2026 will not be measured only by the number of participants, sessions, or abstracts presented. It will be measured by the professionals who were inspired, the services that will improve, the centers that will be created, the research that will arise from new collaborations, and above all, the patients who will have access to better CMR because of the knowledge, confidence, and network built during those four days in Rio de Janeiro.

## Supplemental Materials

For additional information, please click here
